# LOXL2-mediated H3K4 oxidation reduces chromatin accessibility in triple-negative breast cancer cells

**DOI:** 10.1038/s41388-019-0969-1

**Published:** 2019-08-28

**Authors:** J. P. Cebrià-Costa, L. Pascual-Reguant, A. Gonzalez-Perez, G. Serra-Bardenys, J. Querol, M. Cosín, G. Verde, R. A. Cigliano, W. Sanseverino, S. Segura-Bayona, A. Iturbide, D. Andreu, P. Nuciforo, C. Bernado-Morales, V. Rodilla, J. Arribas, J. Yelamos, A. Garcia de Herreros, T. H. Stracker, S. Peiró

**Affiliations:** 1 Vall d’Hebron Institute of Oncology (VHIO), 08035 Barcelona, Spain; 2 Institute for Research in Biomedicine (IRB Barcelona), Barcelona Institute of Science and Technology, 08028 Barcelona, Spain; 3 Faculty of Medicine and Health Sciences, Universitat Internacional de Catalunya, Barcelona, Spain; 4 Sequentia Biotech SL, Comte d’Urgell, 240, Barcelona, Spain; 5 Institute of Epigenetics and Stem Cells, Helmoholtz Zentrum München, D-81377 München, Germany; 6 Departament de Ciències Experimentals i de la Salut, Universitat Pompeu Fabra, Barcelona, Spain; 7 Centro de Investigación Biomédica en Red en Oncología (CIBERONC), 08035 Barcelona, Spain; 8 Institució Catalana de Recerca I Estudis Avançats (ICREA), Barcelona, Spain; 9 Departament de Bioquímica y Biología Molecular, Universitat Autónoma de Barcelona, Bellaterra, Spain; 10 Programa de Recerca en Càncer, Institut Hospital del Mar d’Investigacions Mèdiques (IMIM), Barcelona, Spain

**Keywords:** Chromosomes, Prognostic markers

## Abstract

Oxidation of H3 at lysine 4 (H3K4ox) by lysyl oxidase-like 2 (LOXL2) generates an H3 modification with an unknown physiological function. We find that LOXL2 and H3K4ox are higher in triple-negative breast cancer (TNBC) cell lines and patient-derived xenografts (PDXs) than those from other breast cancer subtypes. ChIP-seq revealed that H3K4ox is located primarily in heterochromatin, where it is involved in chromatin compaction. Knocking down *LOXL2* reduces H3K4ox levels and causes chromatin decompaction, resulting in a sustained activation of the DNA damage response (DDR) and increased susceptibility to anticancer agents. This critical role that LOXL2 and oxidized H3 play in chromatin compaction and DDR suggests that functionally targeting LOXL2 could be a way to sensitize TNBC cells to conventional therapy.

## Introduction

Histone modifications contribute to gene regulation both by directly affecting chromatin structure and by recruiting effector proteins [1]. Deregulation of this enzymatic system can contribute to diseases, including cancer. The lysyl oxidase family of proteins are copper- and quinone-dependent amine oxidases that oxidize the amino group located in the epsilon-position in lysines, thereby generating an aldehyde group [2]. One of the members of the LOX family, lysyl oxidase-like 2 (LOXL2), deaminates unmethylated and trimethylated lysine 4 in histone H3 (H3K4me3) through an amino-oxidase reaction that uses the Cu(II) ion and the internal cofactor lysine-tyrosylquinone, releasing the amino group and converting K4 into an allysine (H3K4ox) [3, 4]. Generation of this peptidyl aldehyde likely alters the local macromolecular structure of chromatin and the nature of any protein–protein or protein–nucleic acid interactions. This is particularly relevant for gene regulation, as changes in the macromolecular status of histones can affect chromatin conformation [4–6].

LOXL2 is overexpressed in many tumors, and especially in breast cancers [7–9]. In this light, it is intriguing that some breast cancers are intrinsically resistant to chemotherapy; for these subtypes, chemotherapy induces a mesenchymal phenotype through the epithelial-to-mesenchymal transition (EMT) [10]. EMT is likely to be a critical switch for tumor cell invasiveness and cell death resistance [11–13] and to involve chromatin reorganization, as it requires dramatic changes in cellular characteristics and gene expression [6, 14]. Notably, the key transcription factor Snail1 interacts with LOXL2 [15], and LOXL2 H3K4 oxidase activity generates an H3K4ox that regulates the repression of the E-cadherin gene (*CDH1*) and heterochromatin transcription, which play roles in two essential steps of EMT [6, 16].

Double-strand breaks (DSBs) are a major form of DNA damage and cause a specific signaling response, the DNA damage response (DDR), which can activate cell cycle checkpoint arrest and cell fate decisions, such as apoptosis or senescence. One of the first steps of DDR is the accumulation of DNA repair proteins at the damaged site [17]. Importantly, DDR activation can occur in the absence of DNA damage, by stable association of the repair factors with chromatin [18, 19]. Moreover, increasing evidence suggests that higher-order chromatin structures affect DSB repair and signaling [20]. For example, DDR actively regulates decondensation of chromatin after DSBs [21], and it is amplified when chromatin is in an “open” state [20]. Similarly, DDR signaling is affected by chromatin compaction in a DNA damage-independent manner [18, 19, 22–24].

We addressed the physiological functions of H3K4ox using an in-house generated antibody specific for this modification to analyze the H3K4ox levels in distinct breast cancer subtypes. Intriguingly, mesenchymal triple-negative breast cancer (TNBC) cell lines as well as breast cancer patient-derived xenografts (PDXs) had high H3K4ox levels that correlated with high LOXL2 expression, as compared with other subtypes. Using ChIP-seq to map its genome-wide localization, we found that H3K4ox was enriched in heterochromatin in TNBC cells, which are highly metastatic and resistant to chemotherapy. Decreasing LOXL2 levels reduced the amount of H3K4ox in chromatin, resulting in chromatin decondensation and a sustained activation of DDR. Further, both *LOXL2* depletion and treatment of TNBC with chromatin-modifying drugs sensitized cancer cells to conventional treatments. Thus, targeting H3K4ox levels may open a new therapeutic window for this subtype of breast cancer.

## Results

### Generating an H3K4ox-specific antibody

**Fig. 1:**
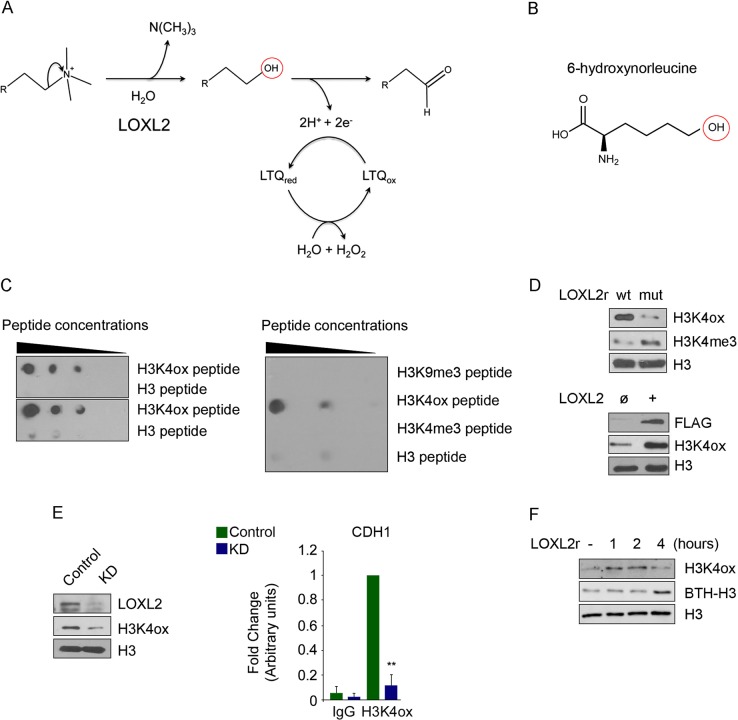
Quality control of the anti-H3K4ox antibody. **a** Schematic representation of the LOXL2 reaction. The red circle indicates the intermediate residue that is targeted by the in-house generated anti-H3K4ox antibody. **b** The artificial amino acid 6-hydroxynorleucine was used in the peptide to generate the anti-H3K4ox antibody. **c** The anti-H3K4ox antibody was found to be specific in western blot in two replicates of dot blots of dilution series of oxidized histone H3 peptide (H3K4ox) or unmodified H3 peptide (left panel), as well as in a representative dot blot of a dilution series of H3K9me3, H3K4ox, H3K4me3, or H3 peptides (right panel). **d** Nucleosomes were incubated with recombinant wild-type (wt) LOXL2 or a catalytically inactive LOXL2 (mut) purified from baculovirus to detect H3K4ox/H3K4me3 levels (upper panel). Lysates of MCF-7 cells transfected with an empty vector (ø) or with *LOXL2* were analyzed by western blotting, using the indicated antibodies (lower panel). **e** Western blot for LOXL2, H3K4ox, and total H3 from MDA-MB-231 cells infected with short hairpin RNA (shRNA) as a control, or a knockdown (KD) using a shRNA specific for LOXL2 (LOXL2 KD) (left panel). Anti-H3K4ox ChIP-PCR was used to analyze the E-cadherin gene (*CDH1*) promoter in MDA-MB-231 cells infected with shRNA for either control (green bar) or *LOXL2* KD (blue bar). Data of qPCR amplifications were normalized to the input and to total H3 for each condition. Error bars indicate the SD from at least three independent experiments. **P < 0.01. **f** Western blot of H3K4ox and biotin incorporation (BTH-H3) in nucleosomes incubated with recombinant LOXL2 purified from baculovirus, after different incubation times

We initially generated a specific antibody for the recently discovered histone modification of H3K4ox, as a prerequisite for studying its physiological function. As the aldehyde group generated by LOXL2 reaction on trimethylated lysine 4 is highly reactive, and hence unfit for immunochemical studies, we hypothesized that a primary alcohol might provide a similar oxygen-bearing functionality that is less reactive, in order to generate a modification-specific antibody that provides a readout of H3K4ox (Fig. 1a). We therefore synthesized a H3 peptide containing a 6-hydroxynorleucine residue as allysine at position 4 and used this for rabbit immunization (Fig. 1b). The resulting H3K4ox antibody was highly specific for the H3K4ox peptide, with very low cross-reactivity for unmodified H3 and no detected cross-reactivity for H3K9me3 or H3K4me3, in a wide range of experimental conditions (dot blots, western blots, and chromatin immunoprecipitation (ChIP) experiments) (Fig. 1c–f). Analysis of purified nucleosomes from 293T cells showed that H3K4ox levels increased, and H3K4me3 levels decreased, when nucleosomes were incubated with wild-type (wt) recombinant LOXL2 but not with a catalytically inactive LOXL2 (LOXL2mut) [3] (Fig. 1d, upper panel). Moreover, the levels of H3K4ox also increased in MCF-7 cells transfected with LOXL2 as compared with cells transfected with the empty vector (Fig. 1d, lower panel). Finally, MDA-MB-231 cells infected with an shRNA targeting the human *LOXL2* (*LOXL2* knockdown (KD)) showed a specific reduction in H3K4ox levels as compared with cells infected with an irrelevant shRNA (control), in both western blots and ChIP-PCR experiments using the E-cadherin gene promoter (*CDH1*), which is a well-known LOXL2-mediated H3K4 oxidation target promoter (Fig. 1e) [3, 4]. Kinetics of the reaction using recombinant LOXL2 and nucleosomes revealed that levels of intermediate alcohol were maintained for 2 h, after which they were reduced (Fig. 1f). Biotinylated hydrazide was used to detect the generated aldehyde group, which appears after 4 h of reaction (Fig. 1f). Thus, as the intermediate alcohol is relatively stable, the antibody we generated can be used as a readout of the oxidized histone H3K4, although we cannot distinguish whether it recognizes the intermediate alcohol, the aldehyde group, or both.

### H3K4ox maps to heterochromatin and controls chromatin accessibility in TNBC cells

**Fig. 2:**
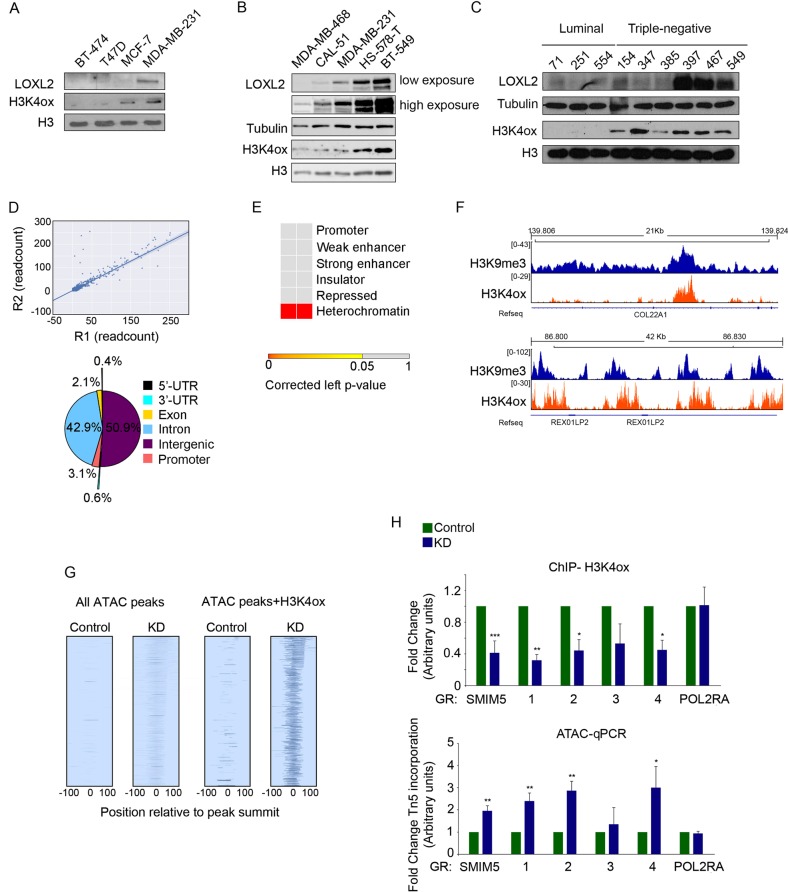
H3K4ox maps to heterochromatin and controls chromatin accessibility in TNBC cells. Western blot for the indicated antibodies in a panel of breast cancer cell lines (**a**), TNBC cell lines (**b**), or PDXs (**c**). **d** Pearson correlation between two H3K4ox sequencing replicates. Distribution of all H3K4ox ChIP-seq peaks in MDA-MB-231 cells are given, with the indicated percentages. **e** Contingency table of the Fisher's exact test showed the statistical overrepresentation of the H3K4ox peaks through different chromatin states. **f** Genome browser view of H3K4ox and H3K9me3-binding profiles at two representative heterochromatin regions. **g** Heatmaps show the ATAC signal in all peaks as well as in peaks that overlap with H3K4ox in *LOXL2* KD or control cells. **h** H3K4ox ChIP-PCR validation of the selected genomic regions from the ChIP-seq from control or *LOXL2* KD MDA-MB-231 cells. Data of qPCR amplification were normalized to the input and to total H3 (upper panel). ATAC-qPCR validation of the incorporation of the transposase Tn5 at the selected genomic regions from the ChIP-seq from control or *LOXL2* KD MDA-MB-231 cells. Data of qPCR amplification were normalized to an unchanging genomic region (the *HPRT* promoter) and expressed as the fold-change relative to data obtained from control cells, which were set to 1 (lower panel). In both experiments, the RNA polymerase II (*POL2RA*) promoter was used as a negative control. Error bars indicate the SD from at least three independent experiments. *P < 0.05, **P < 0.01, ***P < 0.001

As aberrant expression and activity of LOXL2 have been reported in various cancer types [7–9], we checked the levels of LOXL2 and H3K4ox in several breast cancer cell lines representing different subtypes: luminal A, in the T-47D and MCF-7 cell lines (ER^+^/HER2^–^/PR^+/–^); luminal B, in the BT-474 cell line (ER^+^/HER2^+^/PR^+/–^); and basal TNBC, in the human MDA-MB-231 (ER^–^/HER2^–^/PR^–^) cell line [25]. As compared with the other cell lines, MDA-MB-231 (TNBC) showed high levels of LOXL2 and a corresponding enrichment of H3K4ox (Fig. 2a). Levels of H3K4ox also paralleled increases in LOXL2 expression levels in other TNBC cell lines (e.g., MDA-MB-468, CAL-51, HS-578-T, and BT-549), although with variable LOXL2 expression levels (Fig. 2b). Finally, comparing PDXs from luminal (3 PDXs) or TNBC (6 PDXs) subtypes of breast cancer, we found that in all TNBC PDXs the levels of H3K4ox were higher compared with luminal PDXs. In addition, in four out of six TNBC PDXs H3K4ox levels also correlated with high LOXL2 expression. (Fig. 2c).

To elucidate the function of H3K4ox in breast cancer cells, we first performed a ChIP-seq experiment with the anti-H3K4ox antibody to determine the genomic distribution of H3K4ox in the MDA-MB-231 cells. Peaks called using model-based analysis for ChIP-seq (MACS) [26] showed low differences in H3K4ox between two sequencing replicates, with a genome-wide Pearson correlation coefficient of the read count of the two replicates of 0.997 (Fig. 2d, upper panel). We observed that H3K4ox peaks were distributed throughout different genomic elements (Fig. 2d, lower panel). Using the ChromHMM tool [27], we assessed the statistical overrepresentation of the H3K4ox peaks through different chromatin states (promoter, weak or strong enhancer, insulator, repressed, or heterochromatin [nonrepetitive sequences]) and found that H3K4ox peaks were significantly overrepresented in heterochromatin (Fig. 2e, f). As generating an aldehyde in H3 removes a positive charge and creates a very reactive group, we hypothesized that this reaction affects chromatin structure. To test this, we used the assay for transposase-accessible chromatin [28, 29] followed by deep sequencing (ATAC-seq), which exploits the ability of the prokaryotic transposase Tn5 to integrate preferentially into accessible (open) chromatin. ATAC-seq showed an increased ATAC signal in *LOXL2* KD cells, but not with control cells, at H3K4ox-positive sites (Fig. 2g). These results were validated in selected regions by ChIP-qPCR and ATAC-qPCR in control and *LOXL2* KD cells: H3K4ox enrichment decreased in *LOXL2* KD cells, with a correlating increase of ATAC signal, in these regions (Fig. 2h). No changes were observed in a control promoter, POL2RA (Fig. 2h). These data demonstrated that, in the absence of LOXL2, H3K4ox levels decrease and chromatin adopts a more open conformation (Fig. 2g). Thus, our results showed that H3K4ox is enriched in heterochromatin and is directly linked with chromatin accessibility in those regions.

### Chromatin structure alterations activate DDR in a LOXL2-dependent manner

**Fig. 3:**
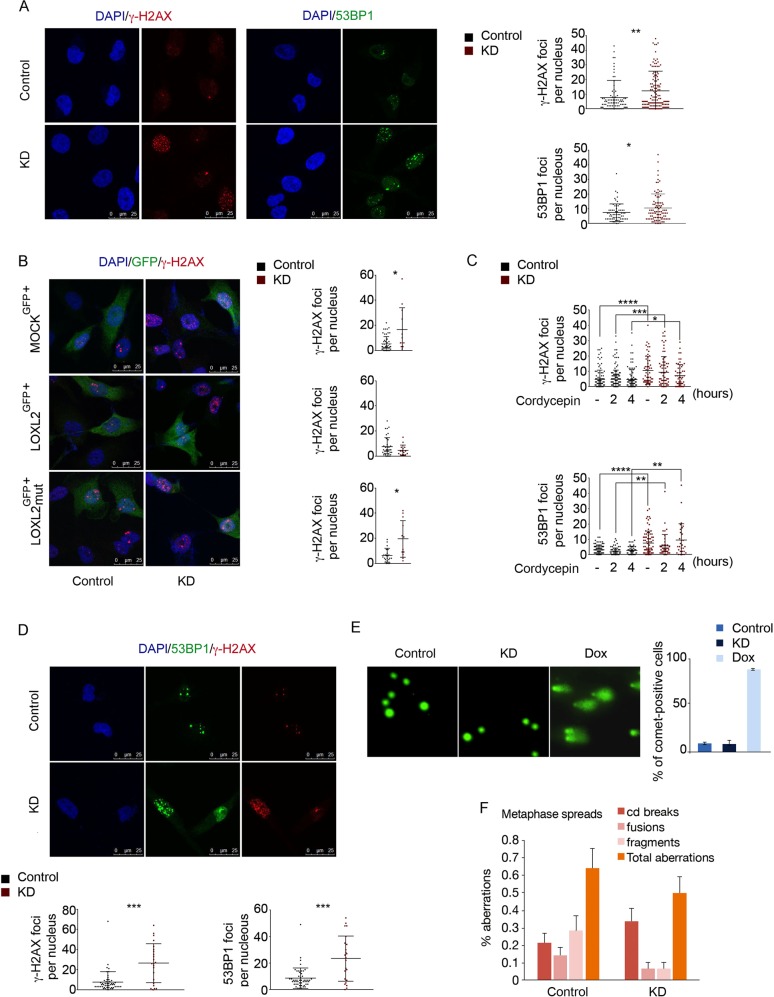
Chromatin opening activates DDR via a mechanism that requires catalytically functional LOXL2 but can be independent of DNA damage. **a** γ-H2AX and 53BP1 staining and foci quantification are shown by immunofluorescence with a specific antibody for γ-H2AX (left image) or for 53BP1 (right image). Dot graphs indicate the number of foci for γ-H2AX (upper graph) and 53BP1 (lower graph) per cell in control and *LOXL2* KD conditions. **b** γ-H2AX staining and foci quantification are shown by immunofluorescence with the indicated antibody after *LOXL2* reinfection. MDA-MB-231 cells were first infected with control or *LOXL2* KD lentivirus and then, after puromycin selection, again with GFP (MOCK^GFP+^), wild-type *LOXL2*-IRES-GFP (LOXL2^GFP+^), or *LOXL2*mut-IRES-GFP (LOXL2mut^GFP+^). Cells were fixed after 24 h. Dot graphs indicate the number of γ-H2AX foci per GFP-positive cells containing MOCK^GFP+^ (upper graph), *LOXL2*^GFP+^ (middle graph), or *LOXL2*mut^GFP+^ (lower graph). **c** Dot graphs indicate the number of γ-H2AX (upper graph) and 53BP1 (lower graph) foci per cell in control and *LOXL2* KD cells after treatment with 200 μM cordycepin for the indicated timepoints. **d** γ-H2AX and 53BP1 staining and foci quantification are shown after immunofluorescence with the indicated antibodies in non-replicative conditions. Dot graphs indicate the number of the γ-H2AX (left graph) and 53BP1 (right graph) foci in control and *LOXL2* KD cells. **e** Representative image showing DNA damage in control or *LOXL2* KD MDA-MB-231 cells, visualized by the alkaline comet assay. Cells were treated with 0.3 μM doxorubicin for 24 h. The graph shows the percentage of MDA-MB-231 positive cells. **f** Chromosome alterations in control and *LOXL2* KD MDA-MB-231 cells. Error bars indicate the SD from at least three independent experiments. *P < 0.05, **P < 0.01, and ***P < 0.001, ****P < 0.0001

As the chromatin state can influence many aspects of DDR [30], we hypothesized that disruption of *LOXL2* expression and impairment of H3K4ox generation might influence DDR by affecting chromatin accessibility. To test this, we analyzed by immunofluorescence MDA-MB-231 cells that had been infected with either *LOXL2* KD or control lentiviruses, using two well-established markers of DDR: phosphorylated H2AX (γ-H2AX) and TP53-binding protein 1 (53BP1). Depletion of LOXL2 (using *LOXL2* KD) led to more foci of both γ-H2AX and 53BP1 than in control cells, suggesting that *LOXL2* KD cells may accumulate DNA breaks and/or activate DDR (Fig. 3a). To determine if the LOXL2 catalytic activity was involved in the observed phenotype, *LOXL2* KD cells were complemented by reinfection with ectopic vector expressing either the wt *LOXL2*-IRES-GFP or a catalytically inactive *LOXL2* (*LOXL2*mut-IRES-GFP), both of which were expressed at similar levels (Fig. S1). Fewer γ-H2AX and 53BP1 foci were observed in *LOXL2* KD cells after reintroduction of the wt (but not of the catalytically inactive) LOXL2 (Fig. 3b), establishing that suppressing DDR activation requires both the activity of LOXL2 and H3K4ox generation.

### *LOXL2* KD activates DDR independently of DNA damage

Increased DDR activation in LOXL2-depleted cells could be a consequence of more DSBs due to reduced H3K4ox levels and/or chromatin decondensation. Notably, aberrant silencing and conflicts between replication forks and transcription, as well as the presence of R-loops (a transcriptional intermediate), can result in DNA damage and are influenced by chromatin state, for example in cells lacking the linker histone H1 [31]. However, our analysis of RNA-seq data revealed that *LOXL2* KD cells did not have altered expression of repetitive elements (Table 1), and DDR activation in *LOXL2* KD cells was not affected by cordycepin, an inhibitor of RNA synthesis that abolishes R-loop formation [32, 33] (Fig. 3c). As R-loops can generate DNA damage during replication due to fork stalling and collapse [32, 34], we next analyzed γ-H2AX and 53BP1 foci in noncycling *LOXL2* KD cells and found DDR activation to also be increased (Fig. 3d). Overall, these data suggested that overexpression of repetitive elements, R-loop formation, and replication fork stalling were not responsible for activating DDR following LOXL2 depletion, when heterochromatin adopts a more open state. As chromatin structure alterations can trigger the DDR, even in the absence of DNA damage [22, 35, 36], we checked for the presence of DNA damage following LOXL2 depletion more directly, using the comet assay, in *LOXL2* KD or control cells under alkaline conditions. No increases in DNA damage (due to either single-strand or double-strand DNA breaks) were observed in *LOXL2* KD cells (Fig. 3e). Moreover, we did not observe any significant differences in chromosomal lesions between *LOXL2* KD or control cells in metaphase spreads (Fig. 3f). Finally, analyzing for mitotic aberrations (anaphase bridges and micronuclei), which can be indicative of replication stress or DNA repair defects, we observed only a mild increase in anaphase bridges in the absence of LOXL2 (Fig. S2). Taken together, our data suggested that the combination of loss of LOXL2 and reduced H3K4ox levels in TNBC cells was sufficient to activate the DDR in the absence of detectable DNA lesions.

**Table 1:** Differential expression analysis between control and *LOXL2* knockdown of repetitive elements

Locus	Ctrl_mean	Loxl2_mean	Prob	log2FC
Alu	3,601,718.96	3,714,313.36	1.00	0.04
RNA	2,918,334.55	3,045,475.41	0.99	0.06
L1	2,837,069.25	2,730,921.52	0.97	−0.06
ERV1	370,405.10	353,869.12	0.95	−0.07
TcMar-Tigger	239,662.63	252,421.97	0.87	0.07
UCON19	0.51	1.23	0.76	1.28
UCON18	0.51	1.23	0.76	1.28
hAT-Tip100	34,122.72	38,990.93	0.76	0.19
ERVK	8,958,641.10	9,448,789.20	0.76	0.08
MIR	673,530.34	692,895.61	0.76	0.04
hAT	12,908.30	16,757.15	0.76	0.38
TcMar-Mariner	33,897.47	38,475.09	0.75	0.18
UCON4	234.62	360.51	0.74	0.62
hAT-Charlie	374,265.61	380,188.93	0.72	0.02
UCON16	6.58	10.85	0.72	0.72
Penelope	7.08	11.83	0.70	0.74
ERVL-MaLR	399,009.16	406,080.51	0.70	0.03
UCON31	61.20	87.49	0.70	0.52
MER130	15.18	21.75	0.69	0.52
UCON2	320.19	434.63	0.69	0.44
UCON10	7.09	10.93	0.67	0.63
UCON28c	10.12	14.81	0.63	0.55
Eulor4	1.26	1.98	0.55	0.65
UCON12	14.14	4.94	0.52	−1.52
TcMar-Tc2	12,524.35	13,658.11	0.51	0.13
UCON24	1.27	0.49	0.47	−1.36
UCON17	3.03	1.23	0.46	−1.30
ERVL	174,857.94	177,852.71	0.44	0.02
MamRep605	7848.87	8610.92	0.41	0.13
UCON28a	201.88	253.61	0.39	0.33
Satellite	28,748.18	25,760.18	0.37	−0.16
UCON26	532.62	618.38	0.35	0.22
SVA_B	2696.77	3025.33	0.31	0.17
acro	14.16	10.88	0.30	−0.38
UCON11	6.08	3.93	0.29	−0.63
UCON15	4.29	2.25	0.29	−0.93
MuDR	1610.89	1799.90	0.28	0.16
SVA_F	3968.24	4322.50	0.26	0.12
Gypsy	18,034.32	16,836.16	0.26	−0.10
DNA	5850.42	6284.72	0.25	0.10
Dong-R4	3696.41	3198.15	0.25	−0.21
UCON9	18.69	12.78	0.24	−0.55
Centre	11,923.53	12,544.21	0.22	0.07
Merlin	297.42	327.87	0.19	0.14
SVA_D	11,475.76	11,978.90	0.19	0.06
UCON28b	26.33	30.80	0.18	0.23
LTR	1842.20	1987.87	0.18	0.11
UCON8	75.37	65.33	0.15	−0.21
TcMar	8328.59	8597.66	0.11	0.05
SVA_A	1741.04	1827.45	0.10	0.07
CR1	85,288.67	84,328.51	0.09	−0.02
Helitron	5017.41	5157.97	0.06	0.04
UCON6	1,277.83	1,325.68	0.06	0.05
RTE	30,497.19	29,901.20	0.06	−0.03
PiggyBac	4590.52	4349.34	0.06	−0.08
UCON22	12.14	10.47	0.06	−0.21
UCON5	11.14	9.87	0.06	−0.17
telo	489.07	507.40	0.05	0.05
L2	614,402.54	610,776.39	0.05	−0.01
Deu	2157.42	2220.57	0.05	0.04
hAT-Blackjack	11,106.77	11,287.96	0.05	0.02
UCON1	3.04	3.25	0.04	0.10
UCON27	72.82	74.65	0.03	0.04
UCON20	12.13	10.95	0.02	−0.15
MamRep564	756.04	712.80	0.02	−0.08
RTE-BovB	1064.66	1011.37	0.02	−0.07
ERV	440.91	422.61	0.01	−0.06
SINE	4912.94	4894.14	0.00	−0.01
UCON25	2.27	2.20	0.00	−0.04
SVA_E	1087.33	1076.64	0.00	−0.01

### Alterations in chromatin compaction activate the DDR

**Fig. 4:**
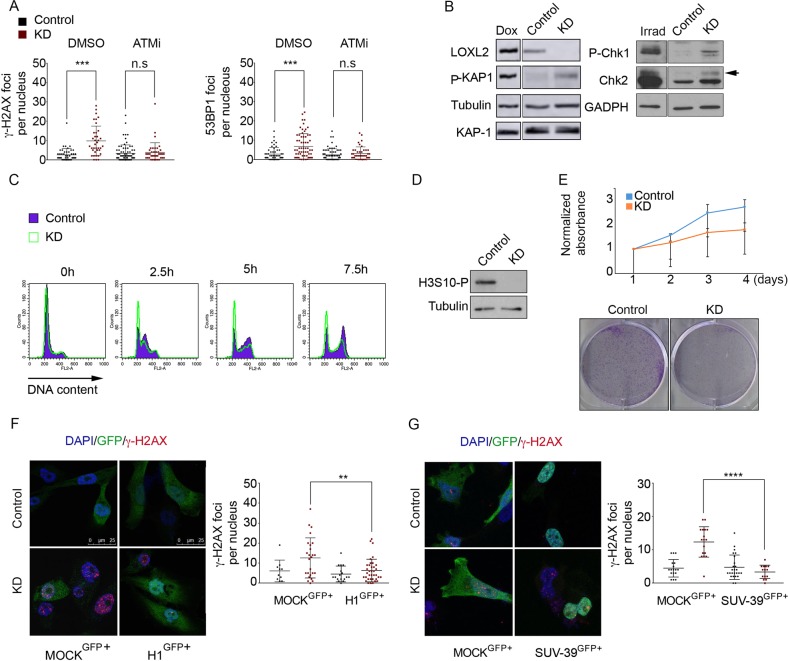
DDR activation is linked to chromatin decondensation in the absence of LOXL2. **a** Dot graphs indicate the number of foci with γ-H2AX (left graph) and 53BP1 (right graph) per cell from control and *LOXL2* KD cells treated with DMSO or the ATM inhibitor KU55933. **b** KAP-1 phosphorylation in control or *LOXL2* KD cells was analyzed by western blot. Tubulin and total KAP-1 were used as loading controls. As a positive control, MDA-MB-231 cells treated with 0.1 µM doxorubicin for 8 h (to generate DSBs) were used. Intervening lanes were removed as indicated (left panel). Chk1 and Chk2 phosphorylation in control and *LOXL2* KD cells were analyzed by western blot. Chk1 phosphorylation was detected using the anti-P(S317) Chk1 antibody. For phosphorylated Chk2, a shift was detected using an anti-total Chk2 antibody. GADPH was used as a loading control. All samples were obtained under the same experimental conditions; in addition, positive control samples (irradiated fibroblasts) were run on the same gel as their corresponding control and *LOXL2* KD samples. Intervening lanes in the Chk1/2 and GADPH blots were removed as indicated (right panel). **c** Cell cycle profile of control and *LOXL2* KD cells at 0, 2.5, 5, or 7.5 h after release from a double-thymidine block. Cells were analyzed by fluorescence activated cell sorting (FACS) after propidium iodide staining. **d** H3S10 phosphorylation levels in control and *LOXL2* KD MDA-MB-231 cells were analyzed by western blot. Tubulin was used as a loading control. **e** Upper panel, MTT assay in control and *LOXL2* KD MDA-MB-231 cells; lower panel, colony-survival assay in control and *LOXL2* KD MDA-MB-231 cells. γ-H2AX staining and foci quantification are shown by immunofluorescence with the indicated antibody after H1 (**f**) or SUV-39H1 reinfection (**g**). MDA-MB-231 cells were infected with control or *LOXL2* KD lentivirus, selected with puromycin, and reinfected with GFP (MOCK^GFP+^), histone 1-GFP (H1^GFP+^) (**f**) or SUV-39H1-GFP (SUV-39^GFP+^) (**g**). Cells were fixed after 24 h. Dot graphs indicate the number of γ-H2AX foci per GFP-positive cell in each condition. Error bars indicate the SD from at least three independent experiments. **P < 0.01, ***P < 0.001, ****P < 0.0001

To further address the origin of DDR signaling in LOXL2-depleted cells, we analyzed the behavior of additional DDR signaling components. As both γ-H2AX and 53BP1 foci formation require the ATM kinase in some settings, we treated *LOXL2* KD and control cells with the ATM inhibitor KU55933 and analyzed foci formation (Fig. 4a). Decreased foci of both markers were observed upon ATM inhibition, indicating that LOXL2-induced DDR was largely ATM-dependent. Consistent with this, *LOXL2* KD cells had increased phosphorylation of several ATM substrates, including KAP-1, CHK1, and CHK2 (Fig. 4b). However, we ruled out that increased DDR signaling was due to apoptosis in *LOXL2* KD cells, as no cleaved caspase-3 signal was observed in either *LOXL2* KD or control cells (Fig. S3a). As these data suggested that the LOXL2 KD cells activated a checkpoint response, we analyzed cell cycle progression following LOXL2 depletion. After synchronization with a double thymidine block, LOXL2 KD cells were not able to efficiently progress through the cell cycle (Fig. 4c), and western blotting for H3S10-P showed that this histone mark was undetectable in *LOXL2* KD cells as compared with control cells. (Fig. 4d). These data strongly suggested that *LOXL2* KD cells arrested primarily in G1. Consistent with this possibility, cell proliferation capacity of *LOXL2* KD cells was blocked (Fig. 4e, upper panel), and their colony-formation capacity was strongly reduced after only a few passages (Fig. 4e, lower panel). This effect on proliferation was further confirm by gene ontology analysis of the gene expression pattern of *LOXL2* KD cells (Fig. S3b; Tables 2 and 3).

**Table 2:** Genes upregulated in *LOXL2* knockdown

Locus	logFC	*P*-Value	FDR	Gene name
ENSG00000106541	2.557531982	0		AGR2
ENSG00000185567	2.176849204	5.15E−299	2.25E−295	AHNAK2
ENSG00000132821	2.080483757	2.77E−280	9.09E−277	VSTM2L
ENSG00000117984	1.586454466	3.61E−273	9.49E−270	CTSD
ENSG00000039139	1.913971451	1.13E−234	1.49E−231	DNAH5
ENSG00000163083	1.827194971	3.38E−216	4.03E−213	INHBB
ENSG00000184371	1.564908962	9.87E−215	1.08E−211	CSF1
ENSG00000170373	1.445267367	7.43E−214	7.51E−211	CST1
ENSG00000183018	2.687264202	2.37E−207	2.22E−204	SPNS2
ENSG00000148180	1.25021061	1.35E−204	1.18E−201	GSN
ENSG00000187678	1.29153331	2.72E−191	2.23E−188	SPRY4
ENSG00000109062	1.468364079	5.45E−183	3.97E−180	SLC9A3R1
ENSG00000124766	1.897033792	1.36E−178	9.39E−176	SOX4
ENSG00000100994	1.081533166	2.15E−168	1.35E−165	PYGB
ENSG00000181634	1.433165343	1.64E−154	8.26E−152	TNFSF15
ENSG00000147676	1.792602889	2.15E−153	1.05E−150	MAL2
ENSG00000107819	1.097217673	1.35E−152	6.33E−150	SFXN3
ENSG00000167644	1.245454774	2.18E−150	9.88E−148	C19orf33
ENSG00000172794	2.37902321	3.41E−147	1.49E−144	RAB37
ENSG00000058085	1.09085532	1.15E−145	4.88E−143	LAMC2
ENSG00000175130	1.074875932	2.20E−139	9.03E−137	MARCKSL1
ENSG00000171345	1.019257679	8.08E−137	3.12E−134	KRT19
ENSG00000167552	1.388799485	1.60E−136	5.82E−134	TUBA1A
ENSG00000132470	1.437708599	1.07E−135	3.79E−133	ITGB4
ENSG00000008513	1.019331762	3.84E−133	1.29E−130	ST3GAL1
ENSG00000167642	0.963304201	1.10E−126	3.43E−124	SPINT2
ENSG00000196154	0.94711039	2.71E−126	8.28E−124	S100A4
ENSG00000161013	1.17382212	9.23E−123	2.64E−120	MGAT4B
ENSG00000144136	0.959244783	4.64E−122	1.30E−119	SLC20A1
ENSG00000124145	0.935893618	1.39E−119	3.73E−117	SDC4
ENSG00000124664	1.067904809	1.15E−118	3.03E−116	SPDEF
ENSG00000108639	0.963584447	1.29E−117	3.32E−115	SYNGR2
ENSG00000099812	2.361113786	2.95E−117	7.18E−115	MISP
ENSG00000153292	1.189984144	2.35E−112	5.32E−110	ADGRF1
ENSG00000071054	0.884375284	1.55E−110	3.39E−108	MAP4K4
ENSG00000163683	1.090539229	6.92E−109	1.49E−106	SMIM14
ENSG00000069122	0.8914899	1.23E−107	2.48E−105	ADGRF5
ENSG00000163346	1.002881721	4.33E−107	8.61E−105	PBXIP1
ENSG00000168032	1.546762635	6.76E−107	1.32E−104	ENTPD3
ENSG00000010404	1.093704162	1.94E−106	3.74E−104	IDS
ENSG00000026751	1.232149717	1.10E−100	1.95E−98	SLAMF7
ENSG00000149177	0.896255656	1.20E−98	2.04E−96	PTPRJ
ENSG00000169213	2.790765942	6.07E−98	1.02E−95	RAB3B
ENSG00000148344	1.010954162	2.71E−97	4.51E−95	PTGES
ENSG00000167232	1.137723821	6.17E−97	9.99E−95	ZNF91
ENSG00000162337	0.931691275	4.04E−96	6.40E−94	LRP5
ENSG00000013364	0.847555049	1.19E−94	1.87E−92	MVP
ENSG00000143061	1.006284582	2.26E−94	3.49E−92	IGSF3
ENSG00000119630	1.384163447	1.38E−93	2.11E−91	PGF
ENSG00000138678	0.902786259	1.84E−90	2.65E−88	GPAT3
ENSG00000125731	1.560012238	2.27E−90	3.24E−88	SH2D3A
ENSG00000101441	0.900355704	3.67E−90	5.18E−88	CST4
ENSG00000174804	1.138994169	1.66E−89	2.32E−87	FZD4
ENSG00000135480	0.885290783	1.96E−89	2.71E−87	KRT7
ENSG00000114554	0.782941224	6.05E−89	8.19E−87	PLXNA1
ENSG00000131981	0.825525944	3.76E−88	5.04E−86	LGALS3
ENSG00000205542	0.843455136	7.86E−87	1.02E−84	TMSB4X
ENSG00000118898	2.469371901	1.48E−86	1.91E−84	PPL
ENSG00000114739	0.98377701	1.49E−86	1.91E−84	ACVR2B
ENSG00000172296	0.928175781	1.83E−86	2.31E−84	SPTLC3
ENSG00000173801	0.93691533	2.54E−86	3.18E−84	JUP
ENSG00000136205	0.893027849	5.42E−85	6.59E−83	TNS3
ENSG00000078246	1.095298427	2.01E−84	2.40E−82	TULP3
ENSG00000165757	1.886253371	7.17E−84	8.48E−82	KIAA1462
ENSG00000158109	0.91823575	1.48E−82	1.69E−80	TPRG1L
ENSG00000115295	0.781412289	7.32E−82	8.29E−80	CLIP4
ENSG00000151651	0.773631649	1.07E−81	1.20E−79	ADAM8
ENSG00000183087	0.797199229	1.18E−80	1.32E−78	GAS6
ENSG00000134531	0.763620701	1.89E−80	2.08E−78	EMP1
ENSG00000071242	1.290957558	2.94E−80	3.20E−78	RPS6KA2
ENSG00000074527	1.161231478	5.89E−80	6.29E−78	NTN4
ENSG00000122359	0.732215153	8.78E−78	9.15E−76	ANXA11
ENSG00000116478	0.727866925	1.16E−77	1.19E−75	HDAC1
ENSG00000065613	0.732219022	1.56E−77	1.58E−75	SLK
ENSG00000108846	1.870849664	1.05E−76	1.06E−74	ABCC3
ENSG00000196975	0.868874265	5.81E−76	5.73E−74	ANXA4
ENSG00000165476	0.732395071	1.31E−75	1.27E−73	REEP3
ENSG00000147251	0.950209054	2.84E−74	2.68E−72	DOCK11
ENSG00000104081	1.619563862	1.16E−73	1.09E−71	BMF
ENSG00000115107	0.833533503	1.66E−73	1.55E−71	STEAP3
ENSG00000146072	0.740689199	2.97E−73	2.71E−71	TNFRSF21
ENSG00000118640	0.8568247	7.92E−73	7.18E−71	VAMP8
ENSG00000198756	1.324358789	1.14E−72	1.02E−70	COLGALT2
ENSG00000161714	0.795194647	1.36E−72	1.22E−70	PLCD3
ENSG00000178726	0.836487297	3.26E−72	2.88E−70	THBD
ENSG00000160271	0.831966049	3.75E−72	3.29E−70	RALGDS
ENSG00000102271	0.886072448	7.99E−72	6.95E−70	KLHL4
ENSG00000112367	0.989558706	2.33E−71	2.01E−69	FIG4
ENSG00000073849	1.156064112	7.50E−71	6.35E−69	ST6GAL1
ENSG00000001461	0.759211956	2.61E−70	2.19E−68	NIPAL3
ENSG00000105778	0.716804361	7.67E−70	6.41E−68	AVL9
ENSG00000180861	2.544226107	5.43E−26	8.20E−25	LINC01559
ENSG00000138764	0.853001536	4.67E−69	3.76E−67	CCNG2
ENSG00000168461	0.739918812	6.17E−69	4.94E−67	RAB31
ENSG00000165474	1.287094496	7.37E−69	5.87E−67	GJB2
ENSG00000117472	1.402524019	3.06E−68	2.41E−66	TSPAN1
ENSG00000124496	0.733809105	7.91E−68	6.04E−66	TRERF1
ENSG00000171877	0.944409394	3.72E−67	2.73E−65	FRMD5
ENSG00000126759	1.579268489	7.91E−67	5.74E−65	CFP
ENSG00000124882	1.027808793	1.06E−66	7.64E−65	EREG
ENSG00000102316	0.67559959	1.29E−66	9.23E−65	MAGED2
ENSG00000075711	0.664598245	4.29E−66	2.99E−64	DLG1
ENSG00000163435	2.261552237	1.24E−64	8.43E−63	ELF3
ENSG00000135048	0.842294959	2.39E−64	1.62E−62	TMEM2
ENSG00000141279	0.798544903	3.43E−64	2.31E−62	NPEPPS
ENSG00000124491	1.928383505	3.87E−64	2.58E−62	F13A1
ENSG00000136068	0.799841098	1.04E−63	6.88E−62	FLNB
ENSG00000170786	1.469743357	2.22E−63	1.45E−61	SDR16C5
ENSG00000069702	0.968766875	2.52E−63	1.64E−61	TGFBR3
ENSG00000069956	0.687274901	3.83E−63	2.48E−61	MAPK6
ENSG00000183826	0.802508399	7.36E−63	4.72E−61	BTBD9
ENSG00000137449	0.953988726	9.13E−63	5.82E−61	CPEB2
ENSG00000160789	0.769443927	1.58E−62	9.90E−61	LMNA
ENSG00000115756	0.635371525	1.24E−60	7.58E−59	HPCAL1
ENSG00000169439	0.774541548	2.36E−60	1.42E−58	SDC2
ENSG00000184564	2.470980644	2.81E−60	1.68E−58	SLITRK6
ENSG00000143816	1.634586486	5.68E−60	3.36E−58	WNT9A
ENSG00000182093	0.87376722	7.53E−60	4.44E−58	WRB
ENSG00000065054	0.668246405	9.12E−60	5.32E−58	SLC9A3R2
ENSG00000116701	1.117244095	1.12E−59	6.48E−58	NCF2
ENSG00000100439	0.801911634	1.37E−59	7.90E−58	ABHD4
ENSG00000101439	0.771824808	1.52E−59	8.73E−58	CST3
ENSG00000167779	0.981583691	1.61E−59	9.18E−58	IGFBP6
ENSG00000075618	0.742779504	1.63E−59	9.25E−58	FSCN1
ENSG00000178585	0.715678973	1.83E−59	1.03E−57	CTNNBIP1
ENSG00000088836	1.218822163	4.54E−59	2.53E−57	SLC4A11
ENSG00000006118	0.63601116	5.56E−59	3.08E−57	TMEM132A
ENSG00000140950	0.734048999	9.06E−59	4.98E−57	TLDC1
ENSG00000114353	0.624135935	1.44E−58	7.86E−57	GNAI2
ENSG00000189143	0.778650604	3.07E−58	1.65E−56	CLDN4
ENSG00000166925	0.750896396	4.93E−58	2.63E−56	TSC22D4
ENSG00000169991	0.777045163	5.47E−58	2.90E−56	IFFO2
ENSG00000198910	0.697815856	1.08E−57	5.67E−56	L1CAM
ENSG00000142627	0.64537199	8.67E−57	4.47E−55	EPHA2
ENSG00000119899	0.702317857	1.04E−56	5.35E−55	SLC17A5
ENSG00000111846	0.636366397	1.06E−56	5.42E−55	GCNT2
ENSG00000100234	0.679728149	2.30E−56	1.15E−54	TIMP3
ENSG00000095203	0.700479323	3.83E−56	1.91E−54	EPB41L4B
ENSG00000151276	0.736006307	6.05E−56	3.00E−54	MAGI1
ENSG00000171310	0.607237912	1.24E−55	6.09E−54	CHST11
ENSG00000165434	0.816777806	2.01E−55	9.82E−54	PGM2L1
ENSG00000154930	1.233947636	5.25E−55	2.55E−53	ACSS1
ENSG00000116285	0.720021425	7.57E−55	3.67E−53	ERRFI1
ENSG00000106799	0.601363577	1.04E−54	5.00E−53	TGFBR1
ENSG00000100055	1.153795642	1.14E−54	5.50E−53	CYTH4
ENSG00000204634	0.78091263	1.43E−54	6.80E−53	TBC1D8
ENSG00000187764	0.934447405	3.48E−54	1.64E−52	SEMA4D
ENSG00000196611	0.697333766	6.80E−54	3.18E−52	MMP1
ENSG00000072210	0.677662089	7.95E−54	3.70E−52	ALDH3A2
ENSG00000166750	0.934437232	1.24E−53	5.75E−52	SLFN5
ENSG00000160213	0.660113084	2.58E−53	1.19E−51	CSTB
ENSG00000154175	1.422923707	3.12E−53	1.43E−51	ABI3BP
ENSG00000105048	0.648949367	3.73E−53	1.71E−51	TNNT1
ENSG00000029534	0.948107565	3.84E−53	1.75E−51	ANK1
ENSG00000163874	0.734961316	1.42E−52	6.44E−51	ZC3H12A
ENSG00000003249	1.44442704	3.21E−52	1.44E−50	DBNDD1
ENSG00000059804	0.834124252	4.53E−52	2.00E−50	SLC2A3
ENSG00000120337	1.718937071	4.63E−52	2.04E−50	TNFSF18
ENSG00000182022	0.664899083	5.48E−52	2.41E−50	CHST15
ENSG00000188191	0.90765776	6.61E−52	2.88E−50	PRKAR1B
ENSG00000003147	1.021914278	1.02E−51	4.40E−50	ICA1
ENSG00000018408	0.61449093	1.18E−51	5.09E−50	WWTR1
ENSG00000188643	0.612623576	1.72E−51	7.37E−50	S100A16
ENSG00000106070	0.622574635	2.67E−51	1.14E−49	GRB10
ENSG00000128342	0.665706893	3.64E−51	1.55E−49	LIF
ENSG00000091409	0.586574997	1.51E−50	6.36E−49	ITGA6
ENSG00000130477	1.980813997	2.31E−50	9.70E−49	UNC13A
ENSG00000059377	1.224196279	4.24E−50	1.76E−48	TBXAS1
ENSG00000167106	0.797725209	5.46E−50	2.26E−48	FAM102A
ENSG00000011009	0.708291693	5.65E−50	2.33E−48	LYPLA2
ENSG00000188042	0.587843927	1.01E−49	4.16E−48	ARL4C
ENSG00000106258	3.99646604	1.07E−49	4.39E−48	CYP3A5
ENSG00000253719	0.641918884	1.98E−49	7.95E−48	ATXN7L3B
ENSG00000198561	0.6435595	2.03E−49	8.11E−48	CTNND1
ENSG00000196642	0.629620891	2.44E−49	9.74E−48	RABL6
ENSG00000162909	0.627093674	4.17E−49	1.66E−47	CAPN2
ENSG00000166046	1.014081966	1.05E−48	4.14E−47	TCP11L2
ENSG00000106537	1.142647315	1.06E−48	4.16E−47	TSPAN13
ENSG00000153179	0.654407199	1.21E−48	4.77E−47	RASSF3
ENSG00000197442	0.704516379	1.30E−48	5.09E−47	MAP3K5
ENSG00000138119	0.641567321	6.81E−48	2.62E−46	MYOF
ENSG00000185483	0.88479338	9.18E−48	3.51E−46	ROR1
ENSG00000184363	0.800404785	1.32E−47	5.02E−46	PKP3
ENSG00000008083	0.690696827	1.62E−47	6.16E−46	JARID2
ENSG00000179913	1.195221395	1.63E−47	6.18E−46	B3GNT3
ENSG00000185127	0.709916588	1.79E−47	6.75E−46	C6orf120
ENSG00000177106	0.686183661	4.22E−47	1.58E−45	EPS8L2
ENSG00000188015	0.884405539	4.58E−47	1.71E−45	S100A3
ENSG00000147400	0.694830612	1.06E−46	3.90E−45	CETN2
ENSG00000143786	0.939975104	2.19E−46	8.05E−45	CNIH3
ENSG00000133805	0.873822055	7.29E−46	2.63E−44	AMPD3
ENSG00000106780	0.689300805	7.57E−46	2.72E−44	MEGF9
ENSG00000272398	1.043194872	2.99E−45	1.06E−43	CD24
ENSG00000099998	1.065481895	6.33E−45	2.23E−43	GGT5
ENSG00000095383	0.635665876	1.03E−44	3.62E−43	TBC1D2
ENSG00000109436	0.653570925	1.05E−44	3.65E−43	TBC1D9
ENSG00000198624	0.606978398	4.62E−44	1.58E−42	CCDC69
ENSG00000153774	0.642071127	5.74E−44	1.96E−42	CFDP1
ENSG00000130958	0.846711873	6.23E−44	2.11E−42	SLC35D2
ENSG00000164938	0.883485618	8.44E−44	2.86E−42	TP53INP1
ENSG00000163947	0.753394922	9.40E−44	3.17E−42	ARHGEF3
ENSG00000182809	1.082621723	2.23E−43	7.43E−42	CRIP2
ENSG00000101842	2.125670418	3.17E−43	1.05E−41	VSIG1
ENSG00000010278	0.692915131	3.75E−43	1.24E−41	CD9
ENSG00000141753	0.652587531	4.03E−43	1.33E−41	IGFBP4
ENSG00000071575	0.809537681	4.69E−43	1.53E−41	TRIB2
ENSG00000041353	0.600506668	6.85E−43	2.23E−41	RAB27B
ENSG00000124942	0.878776788	8.14E−43	2.64E−41	AHNAK
ENSG00000130702	0.830408106	1.66E−42	5.34E−41	LAMA5
ENSG00000106605	0.707072414	1.69E−42	5.40E−41	BLVRA
ENSG00000160211	0.671328183	1.71E−42	5.47E−41	G6PD
ENSG00000153395	0.667145468	1.73E−42	5.50E−41	LPCAT1
ENSG00000183778	1.193744584	3.32E−42	1.05E−40	B3GALT5
ENSG00000131389	0.90704002	2.13E−41	6.66E−40	SLC6A6
ENSG00000184117	0.709961941	2.66E−41	8.24E−40	NIPSNAP1
ENSG00000258947	1.044442933	2.99E−41	9.27E−40	TUBB3
ENSG00000180667	0.586841506	3.12E−41	9.64E−40	YOD1
ENSG00000157227	0.607608231	3.40E−41	1.05E−39	MMP14
ENSG00000184557	0.61191017	1.37E−40	4.18E−39	SOCS3
ENSG00000100106	0.606368936	2.00E−40	6.06E−39	TRIOBP
ENSG00000165233	0.647236635	2.07E−40	6.24E−39	CARD19
ENSG00000126458	0.806490749	2.10E−40	6.33E−39	RRAS
ENSG00000152217	1.432604587	3.42E−40	1.01E−38	SETBP1
ENSG00000157796	0.64712781	4.11E−40	1.22E−38	WDR19
ENSG00000118960	0.794746258	4.94E−40	1.46E−38	HS1BP3
ENSG00000078269	0.598064657	1.32E−39	3.86E−38	SYNJ2
ENSG00000133138	1.209003045	1.35E−39	3.94E−38	TBC1D8B
ENSG00000088387	0.585401778	1.40E−39	4.07E−38	DOCK9
ENSG00000251191	2.434425725	2.57E−38	6.95E−37	LINC00589
ENSG00000203697	4.744840946	1.53E−39	4.43E−38	CAPN8
ENSG00000158769	0.653902818	1.73E−39	5.00E−38	F11R
ENSG00000062282	0.689520114	2.55E−39	7.25E−38	DGAT2
ENSG00000184497	0.978103665	2.65E−39	7.52E−38	TMEM255B
ENSG00000149948	0.723373492	2.80E−39	7.93E−38	HMGA2
ENSG00000148730	0.588607271	3.00E−39	8.47E−38	EIF4EBP2
ENSG00000117983	2.62243558	9.23E−39	2.56E−37	MUC5B
ENSG00000105514	0.898143653	1.15E−38	3.16E−37	RAB3D
ENSG00000169432	1.168069383	1.36E−38	3.72E−37	SCN9A
ENSG00000006534	0.83694272	1.56E−38	4.27E−37	ALDH3B1
ENSG00000074219	0.725674533	1.92E−38	5.24E−37	TEAD2
ENSG00000255471	1.5585602	4.89E−14	3.52E−13	AP001528.2
ENSG00000005238	0.737875746	2.60E−38	7.02E−37	FAM214B
ENSG00000205426	0.859387072	6.01E−38	1.59E−36	KRT81
ENSG00000258088	1.553544167	6.65E−19	6.78E−18	AC078820.1
ENSG00000138162	0.62551312	7.91E−38	2.08E−36	TACC2
ENSG00000141524	0.709114969	8.26E−38	2.17E−36	TMC6
ENSG00000170271	2.264074711	8.41E−38	2.20E−36	FAXDC2
ENSG00000136720	0.700335625	8.54E−38	2.23E−36	HS6ST1
ENSG00000162591	1.561892509	9.75E−38	2.53E−36	MEGF6
ENSG00000173334	0.641809108	1.16E−37	3.02E−36	TRIB1
ENSG00000110871	0.728862751	2.35E−37	6.03E−36	COQ5
ENSG00000150471	1.085455968	2.61E−37	6.68E−36	ADGRL3
ENSG00000133985	1.071387544	2.67E−37	6.82E−36	TTC9
ENSG00000165806	0.615818489	3.03E−37	7.70E−36	CASP7
ENSG00000159733	0.690251958	3.10E−37	7.86E−36	ZFYVE28
ENSG00000125798	0.586210217	3.58E−37	9.07E−36	FOXA2
ENSG00000146278	0.580483004	5.25E−37	1.32E−35	PNRC1
ENSG00000131746	2.387958603	3.96E−36	9.78E−35	TNS4
ENSG00000137767	0.667955094	5.74E−36	1.40E−34	SQOR
ENSG00000054277	1.0616567	6.32E−36	1.54E−34	OPN3
ENSG00000116016	0.598281377	1.05E−35	2.53E−34	EPAS1
ENSG00000151690	0.658340738	1.15E−35	2.77E−34	MFSD6
ENSG00000168487	0.664252835	1.26E−35	3.02E−34	BMP1
ENSG00000144642	0.724358105	2.62E−35	6.20E−34	RBMS3
ENSG00000146386	0.582324614	3.06E−35	7.20E−34	ABRACL
ENSG00000109321	0.85796308	6.09E−35	1.42E−33	AREG
ENSG00000185033	0.613573094	7.11E−35	1.65E−33	SEMA4B
ENSG00000124143	1.002818698	1.31E−34	3.02E−33	ARHGAP40
ENSG00000188766	1.007070905	1.37E−34	3.15E−33	SPRED3
ENSG00000143198	0.665742779	1.45E−34	3.31E−33	MGST3
ENSG00000138772	0.618561607	1.92E−34	4.38E−33	ANXA3
ENSG00000153208	0.65916003	2.75E−34	6.24E−33	MERTK
ENSG00000105379	0.650414245	3.72E−34	8.40E−33	ETFB
ENSG00000174600	1.152413998	4.99E−34	1.12E−32	CMKLR1
ENSG00000143878	0.677873378	6.11E−34	1.36E−32	RHOB
ENSG00000129636	0.586007283	7.21E−34	1.60E−32	ITFG1
ENSG00000196358	1.157286665	7.31E−34	1.62E−32	NTNG2
ENSG00000074855	0.727005968	1.29E−33	2.85E−32	ANO8
ENSG00000179869	1.478231849	1.76E−33	3.84E−32	ABCA13
ENSG00000164379	0.688194901	2.27E−33	4.92E−32	FOXQ1
ENSG00000082458	0.603787281	4.54E−33	9.68E−32	DLG3
ENSG00000186106	0.614744197	1.44E−32	3.01E−31	ANKRD46
ENSG00000127863	0.807228706	1.48E−32	3.09E−31	TNFRSF19
ENSG00000007384	0.675083802	1.79E−32	3.71E−31	RHBDF1
ENSG00000175938	0.991028089	1.90E−32	3.92E−31	ORAI3
ENSG00000258245	1.54291117	1.93E−09	9.27E−09	RPL10P13
ENSG00000032742	0.85419356	4.05E−32	8.32E−31	IFT88
ENSG00000229512	1.425346349	8.83E−13	5.81E−12	AC068580.1
ENSG00000187164	0.600256967	5.52E−32	1.12E−30	SHTN1
ENSG00000154027	1.341272323	9.88E−32	1.99E−30	AK5
ENSG00000101846	0.734435125	1.11E−31	2.23E−30	STS
ENSG00000206190	0.787741284	1.70E−31	3.36E−30	ATP10A
ENSG00000160255	0.651457256	2.24E−31	4.40E−30	ITGB2
ENSG00000101213	0.990447452	4.59E−31	8.91E−30	PTK6
ENSG00000198286	0.883579653	5.70E−31	1.10E−29	CARD11
ENSG00000106066	0.619864757	7.17E−31	1.38E−29	CPVL
ENSG00000115919	0.677292783	8.14E−31	1.56E−29	KYNU
ENSG00000064787	4.909061593	9.51E−31	1.81E−29	BCAS1
ENSG00000144218	0.616205371	1.12E−30	2.13E−29	AFF3
ENSG00000123095	0.933963389	1.93E−30	3.62E−29	BHLHE41
ENSG00000148671	1.02789778	2.52E−30	4.69E−29	ADIRF
ENSG00000258077	1.387892381	1.19E−21	1.42E−20	AC078923.1
ENSG00000125534	0.590391835	7.90E−30	1.44E−28	PPDPF
ENSG00000137501	0.668063928	1.04E−29	1.88E−28	SYTL2
ENSG00000106351	0.728533997	1.04E−29	1.88E−28	AGFG2
ENSG00000185585	0.755238419	1.08E−29	1.95E−28	OLFML2A
ENSG00000178951	0.587643267	1.31E−29	2.35E−28	ZBTB7A
ENSG00000111110	0.952754467	1.54E−29	2.76E−28	PPM1H
ENSG00000047056	0.582933075	2.95E−29	5.19E−28	WDR37
ENSG00000227039	1.384680791	6.64E−17	5.90E−16	ITGB2-AS1
ENSG00000102096	0.607774377	7.04E−29	1.21E−27	PIM2
ENSG00000158106	0.851822142	1.01E−28	1.74E−27	RHPN1
ENSG00000133943	0.621897984	1.44E−28	2.45E−27	C14orf159
ENSG00000132274	0.927124554	1.44E−28	2.46E−27	TRIM22
ENSG00000167565	0.59119894	2.89E−28	4.87E−27	SERTAD3
ENSG00000146416	0.722563478	9.98E−28	1.64E−26	AIG1
ENSG00000102934	1.156498397	3.17E−27	5.08E−26	PLLP
ENSG00000157613	0.66005375	9.58E−27	1.50E−25	CREB3L1
ENSG00000139636	0.759504331	1.05E−26	1.64E−25	LMBR1L
ENSG00000124313	0.956039086	1.06E−26	1.65E−25	IQSEC2
ENSG00000167778	0.584757438	1.37E−26	2.12E−25	SPRYD3
ENSG00000117305	0.692063114	2.15E−26	3.30E−25	HMGCL
ENSG00000177854	1.052435249	3.25E−26	4.95E−25	TMEM187
ENSG00000175505	0.659053782	3.46E−26	5.27E−25	CLCF1
ENSG00000135596	0.588183448	4.27E−26	6.47E−25	MICAL1
ENSG00000259974	1.265560114	1.32E−08	5.80E−08	LINC00261
ENSG00000039068	1.784191229	5.61E−26	8.46E−25	CDH1
ENSG00000139044	2.17079755	6.20E−26	9.34E−25	B4GALNT3
ENSG00000160446	0.580686241	8.53E−26	1.28E−24	ZDHHC12
ENSG00000112679	0.651511201	1.02E−25	1.53E−24	DUSP22
ENSG00000158292	0.659371473	1.14E−25	1.69E−24	GPR153
ENSG00000167470	0.746743002	1.27E−25	1.88E−24	MIDN
ENSG00000100246	0.975110479	4.43E−25	6.45E−24	DNAL4
ENSG00000116117	0.702904005	5.86E−25	8.50E−24	PARD3B
ENSG00000100290	1.23971931	7.52E−25	1.08E−23	BIK
ENSG00000165949	0.802081907	1.33E−24	1.89E−23	IFI27
ENSG00000169083	0.656064782	1.91E−24	2.68E−23	AR
ENSG00000109610	0.77544685	2.24E−24	3.13E−23	SOD3
ENSG00000140931	0.704129083	2.81E−24	3.90E−23	CMTM3
ENSG00000152128	0.697760811	3.49E−24	4.83E−23	TMEM163
ENSG00000124406	0.610126555	4.99E−24	6.83E−23	ATP8A1
ENSG00000106069	1.002738235	6.13E−24	8.36E−23	CHN2
ENSG00000144455	0.66426688	6.74E−24	9.15E−23	SUMF1
ENSG00000163898	0.637729579	7.68E−24	1.04E−22	LIPH
ENSG00000120306	0.686361467	7.96E−24	1.07E−22	CYSTM1
ENSG00000171992	0.821352578	8.82E−24	1.19E−22	SYNPO
ENSG00000275342	0.707225979	8.83E−24	1.19E−22	PRAG1
ENSG00000140022	1.275607654	1.75E−23	2.33E−22	STON2
ENSG00000006453	0.608949037	3.37E−23	4.41E−22	BAIAP2L1
ENSG00000102547	0.7411813	3.62E−23	4.72E−22	CAB39L
ENSG00000162105	1.018126629	3.82E−23	4.98E−22	SHANK2
ENSG00000150782	0.667724476	4.97E−23	6.41E−22	IL18
ENSG00000063660	0.62014441	5.38E−23	6.92E−22	GPC1
ENSG00000162869	0.633902575	6.97E−23	8.91E−22	PPP1R21
ENSG00000065361	0.802225109	7.32E−23	9.35E−22	ERBB3
ENSG00000081189	0.666188328	7.32E−23	9.35E−22	MEF2C
ENSG00000122176	1.592831251	2.05E−22	2.55E−21	FMOD
ENSG00000069812	1.132970411	2.34E−22	2.90E−21	HES2
ENSG00000146530	0.946186656	2.74E−22	3.39E−21	VWDE
ENSG00000249279	1.258589847	3.01E−07	1.13E−06	LINC02057
ENSG00000151715	1.313755414	4.48E−22	5.44E−21	TMEM45B
ENSG00000100031	0.782239579	6.38E−22	7.72E−21	GGT1
ENSG00000225339	1.250563168	1.16E−21	1.38E−20	AL354740.1
ENSG00000230439	1.230428653	3.86E−30	7.17E−29	AL512488.1
ENSG00000105429	0.589326615	1.81E−21	2.15E−20	MEGF8
ENSG00000143344	0.598376949	1.82E−21	2.16E−20	RGL1
ENSG00000114805	0.907966339	2.84E−21	3.32E−20	PLCH1
ENSG00000105856	0.586467521	3.20E−21	3.71E−20	HBP1
ENSG00000188153	0.668030888	3.63E−21	4.19E−20	COL4A5
ENSG00000198513	0.787106569	3.85E−21	4.44E−20	ATL1
ENSG00000113721	1.215644215	4.56E−21	5.25E−20	PDGFRB
ENSG00000183828	0.751113819	4.82E−21	5.54E−20	NUDT14
ENSG00000181722	0.807166236	5.79E−21	6.63E−20	ZBTB20
ENSG00000164976	0.825285155	6.78E−21	7.69E−20	KIAA1161
ENSG00000148737	0.823410224	7.52E−21	8.51E−20	TCF7L2
ENSG00000167861	0.647865569	8.61E−21	9.72E−20	HID1
ENSG00000188747	1.012209238	1.04E−20	1.17E−19	NOXA1
ENSG00000159314	0.882984133	1.11E−20	1.25E−19	ARHGAP27
ENSG00000089723	0.80542898	1.35E−20	1.51E−19	OTUB2
ENSG00000088899	0.649788574	1.38E−20	1.55E−19	LZTS3
ENSG00000154864	0.783916945	2.24E−20	2.49E−19	PIEZO2
ENSG00000122515	0.582145115	2.89E−20	3.19E−19	ZMIZ2
ENSG00000260461	1.216719543	3.67E−09	1.72E−08	AL133355.1
ENSG00000167105	0.61082311	3.72E−20	4.07E−19	TMEM92
ENSG00000198429	0.697252261	3.90E−20	4.26E−19	ZNF69
ENSG00000102452	0.765799284	4.92E−20	5.35E−19	NALCN
ENSG00000108375	1.363173418	7.55E−20	8.14E−19	RNF43
ENSG00000218014	1.189647936	1.64E−08	7.14E−08	KRT19P1
ENSG00000133056	1.110173291	1.42E−19	1.51E−18	PIK3C2B
ENSG00000142347	1.005039505	1.63E−19	1.74E−18	MYO1F
ENSG00000267284	1.148059389	1.95E−09	9.39E−09	AC022031.2
ENSG00000119943	0.668952392	1.86E−19	1.96E−18	PYROXD2
ENSG00000106003	0.591285678	1.90E−19	2.00E−18	LFNG
ENSG00000240583	1.981897469	2.32E−19	2.43E−18	AQP1
ENSG00000280303	1.116972088	5.78E−09	2.65E−08	AC067931.2
ENSG00000125772	0.588258566	3.68E−19	3.83E−18	GPCPD1
ENSG00000185189	0.713266249	6.53E−19	6.67E−18	NRBP2
ENSG00000243629	1.089891447	5.78E−11	3.26E−10	LINC00880
ENSG00000136859	0.625902802	1.32E−18	1.33E−17	ANGPTL2
ENSG00000170412	1.064761214	1.47E−18	1.48E−17	GPRC5C
ENSG00000115525	0.786519457	1.70E−18	1.70E−17	ST3GAL5
ENSG00000108551	0.634579806	2.01E−18	1.99E−17	RASD1
ENSG00000260877	1.040510062	3.58E−10	1.85E−09	AP005233.2
ENSG00000111452	0.797580152	3.43E−18	3.34E−17	ADGRD1
ENSG00000164010	0.671003676	4.05E−18	3.92E−17	ERMAP
ENSG00000267013	1.031866824	1.81E−19	1.91E−18	LINC01929
ENSG00000196517	0.601022218	7.40E−18	7.02E−17	SLC6A9
ENSG00000184985	0.953793323	8.27E−18	7.82E−17	SORCS2
ENSG00000073350	1.015768255	9.33E−18	8.78E−17	LLGL2
ENSG00000160181	0.979528456	9.78E−18	9.19E−17	TFF2
ENSG00000170214	0.985557139	1.03E−17	9.71E−17	ADRA1B
ENSG00000164050	1.092459663	1.60E−17	1.48E−16	PLXNB1
ENSG00000232611	1.014419347	2.24E−07	8.55E−07	AL683813.1
ENSG00000138286	1.012503764	2.56E−17	2.35E−16	FAM149B1
ENSG00000204852	0.586136292	2.61E−17	2.39E−16	TCTN1
ENSG00000107968	0.672186642	2.73E−17	2.50E−16	MAP3K8
ENSG00000103196	0.669497228	3.21E−17	2.93E−16	CRISPLD2
ENSG00000163694	0.664725312	3.76E−17	3.41E−16	RBM47
ENSG00000118777	0.63684278	4.00E−17	3.62E−16	ABCG2
ENSG00000165272	0.737450886	5.14E−17	4.61E−16	AQP3
ENSG00000234405	1.012989861	1.17E−05	3.61E−05	Z69733.1
ENSG00000204282	0.999042357	2.05E−16	1.76E−15	TNRC6C-AS1
ENSG00000160180	1.828562389	1.07E−16	9.33E−16	TFF3
ENSG00000091490	1.064775	1.12E−16	9.71E−16	SEL1L3
ENSG00000174456	0.697982291	1.56E−16	1.35E−15	C12orf76
ENSG00000224184	0.998094957	1.45E−12	9.33E−12	MIR3681HG
ENSG00000139182	0.612905821	2.09E−16	1.79E−15	CLSTN3
ENSG00000021762	0.664520536	2.82E−16	2.40E−15	OSBPL5
ENSG00000024422	0.631697665	2.90E−16	2.47E−15	EHD2
ENSG00000178209	0.59391065	4.21E−16	3.53E−15	PLEC
ENSG00000213626	0.748895824	4.77E−16	3.99E−15	LBH
ENSG00000203709	0.989260567	3.38E−19	3.53E−18	C1orf132
ENSG00000120899	0.809869697	6.04E−16	5.03E−15	PTK2B
ENSG00000166171	0.634006308	6.79E−16	5.63E−15	DPCD
ENSG00000119514	1.109015536	8.36E−16	6.87E−15	GALNT12
ENSG00000166145	1.012449601	9.25E−16	7.55E−15	SPINT1
ENSG00000139625	0.597222522	9.56E−16	7.80E−15	MAP3K12
ENSG00000130529	1.072374016	1.03E−15	8.41E−15	TRPM4
ENSG00000089639	0.583026836	1.04E−15	8.48E−15	GMIP
ENSG00000164362	0.96421019	1.42E−15	1.15E−14	TERT
ENSG00000260265	0.980352322	6.53E−06	2.08E−05	AC110760.4
ENSG00000260832	0.972908743	2.37E−05	7.03E−05	AC125793.1
ENSG00000143224	0.603143848	1.54E−15	1.24E−14	PPOX
ENSG00000166387	0.768430513	1.83E−15	1.47E−14	PPFIBP2
ENSG00000012124	0.751559289	2.74E−15	2.17E−14	CD22
ENSG00000073282	0.941931414	2.98E−15	2.35E−14	TP63
ENSG00000234424	0.96208621	1.12E−05	3.44E−05	AL353743.4
ENSG00000162078	1.302273759	3.84E−15	3.01E−14	ZG16B
ENSG00000170190	0.646867941	5.64E−15	4.38E−14	SLC16A5
ENSG00000277879	0.946436382	1.64E−06	5.64E−06	AL391988.1
ENSG00000182578	0.871074337	7.57E−15	5.81E−14	CSF1R
ENSG00000204442	0.613852863	8.56E−15	6.55E−14	FAM155A
ENSG00000084764	1.238040706	9.29E−15	7.09E−14	MAPRE3
ENSG00000187653	0.90344409	7.66E−38	2.02E−36	TMSB4XP8
ENSG00000125864	0.605676615	1.40E−14	1.06E−13	BFSP1
ENSG00000004799	0.72648243	1.43E−14	1.07E−13	PDK4
ENSG00000164690	1.021512855	1.43E−14	1.08E−13	SHH
ENSG00000158006	0.675401351	1.52E−14	1.14E−13	PAFAH2
ENSG00000102466	1.99530516	1.53E−14	1.14E−13	FGF14
ENSG00000107902	0.928721406	2.36E−14	1.74E−13	LHPP
ENSG00000163219	1.470672943	2.40E−14	1.77E−13	ARHGAP25
ENSG00000105327	0.659101322	2.47E−14	1.82E−13	BBC3
ENSG00000232931	0.879123796	1.35E−07	5.27E−07	LINC00342
ENSG00000116885	0.944805119	2.77E−14	2.04E−13	OSCP1
ENSG00000196542	2.000172114	3.44E−14	2.51E−13	SPTSSB
ENSG00000141469	1.257620277	4.10E−14	2.98E−13	SLC14A1
ENSG00000207954	0.864355996	6.57E−05	0.0001819	MIR138-1
ENSG00000269896	0.863999249	1.98E−06	6.72E−06	AL513477.1
ENSG00000183317	1.598192927	7.07E−14	5.03E−13	EPHA10
ENSG00000064547	1.043247745	7.64E−14	5.43E−13	LPAR2
ENSG00000183454	1.361311685	8.02E−14	5.69E−13	GRIN2A
ENSG00000258545	0.861885379	2.73E−05	8.03E−05	RHOXF1-AS1
ENSG00000171174	0.721579865	1.15E−13	8.07E−13	RBKS
ENSG00000226332	0.827459238	1.96E−10	1.04E−09	AL354836.1
ENSG00000215769	0.819562549	6.81E−12	4.15E−11	ARHGAP27P1-BPTFP1-KPNA2P3
ENSG00000069424	0.926219371	2.49E−13	1.70E−12	KCNAB2
ENSG00000203780	1.268825864	2.79E−13	1.90E−12	FANK1
ENSG00000147642	0.674353182	3.40E−13	2.30E−12	SYBU
ENSG00000261786	0.80298847	9.74E−14	6.87E−13	AC006058.1
ENSG00000224086	0.750479112	5.45E−07	1.98E−06	AC245452.1
ENSG00000251562	0.749923658	6.90E−14	4.92E−13	MALAT1
ENSG00000108821	0.721390767	5.89E−13	3.92E−12	COL1A1
ENSG00000163993	0.950679444	6.17E−13	4.10E−12	S100P
ENSG00000119686	1.315494173	8.81E−13	5.80E−12	FLVCR2
ENSG00000267481	0.748507005	0.00019344	0.000499816	AC011477.2
ENSG00000161677	0.65441487	8.86E−13	5.82E−12	JOSD2
ENSG00000168477	1.714597499	9.18E−13	6.02E−12	TNXB
ENSG00000167535	0.66107174	1.08E−12	7.02E−12	CACNB3
ENSG00000188177	1.190091354	1.32E−12	8.55E−12	ZC3H6
ENSG00000277778	0.730989212	3.18E−07	1.19E−06	PGM5P2
ENSG00000099889	0.874014164	2.18E−12	1.39E−11	ARVCF
ENSG00000257315	0.726622407	2.48E−12	1.57E−11	ZBED6
ENSG00000161395	0.681576338	2.54E−12	1.61E−11	PGAP3
ENSG00000283646	0.715148035	5.47E−13	3.66E−12	LINC02009
ENSG00000274307	0.705188548	0.000339574	0.000836261	AC023449.2
ENSG00000167103	1.332705616	4.92E−12	3.05E−11	PIP5KL1
ENSG00000159423	0.611704098	6.38E−12	3.90E−11	ALDH4A1
ENSG00000278897	0.70098086	0.000273415	0.000687648	AC020951.1
ENSG00000162426	1.314896899	8.38E−12	5.07E−11	SLC45A1
ENSG00000188064	0.633873994	1.16E−11	6.95E−11	WNT7B
ENSG00000187952	0.691227099	0.000538949	0.001287183	HS6ST1P1
ENSG00000108641	0.676095788	1.31E−11	7.82E−11	B9D1
ENSG00000139178	0.828600996	1.34E−11	7.98E−11	C1RL
ENSG00000253669	0.690702691	6.08E−09	2.78E−08	AP003356.1
ENSG00000281881	0.679695644	1.99E−05	5.96E−05	SPRY4-IT1
ENSG00000176371	0.682561022	3.05E−11	1.75E−10	ZSCAN2
ENSG00000066248	0.600563626	3.22E−11	1.85E−10	NGEF
ENSG00000239713	0.790898223	3.30E−11	1.89E−10	APOBEC3G
ENSG00000084710	0.814302414	4.97E−11	2.82E−10	EFR3B
ENSG00000213315	0.674927745	0.001017341	0.002306342	AL122020.1
ENSG00000233895	0.671323329	2.93E−05	8.55E−05	AL121761.1
ENSG00000205593	1.297364282	7.30E−11	4.05E−10	DENND6B
ENSG00000173156	0.660509744	7.44E−11	4.13E−10	RHOD
ENSG00000129757	1.280060934	8.31E−11	4.60E−10	CDKN1C
ENSG00000165929	1.580465307	1.11E−10	6.07E−10	TC2N
ENSG00000111319	0.916384563	1.54E−10	8.30E−10	SCNN1A
ENSG00000226137	0.662532844	2.08E−17	1.92E−16	BAIAP2-AS1
ENSG00000131779	0.645897178	1.88E−10	1.00E−09	PEX11B
ENSG00000149972	1.274126912	1.95E−10	1.04E−09	CNTN5
ENSG00000250072	0.661140207	3.69E−05	0.000106104	AC091940.1
ENSG00000158458	0.771645473	2.17E−10	1.15E−09	NRG2
ENSG00000171443	0.620783725	2.33E−10	1.23E−09	ZNF524
ENSG00000233452	0.657023126	0.001884719	0.004066423	STXBP5-AS1
ENSG00000146966	0.580752824	3.35E−10	1.74E−09	DENND2A
ENSG00000245849	0.649770435	0.001938789	0.004172798	RAD51-AS1
ENSG00000220785	0.643682307	0.000249588	0.000632447	MTMR9LP
ENSG00000160703	0.613565082	3.93E−10	2.02E−09	NLRX1
ENSG00000006756	0.786505348	4.19E−10	2.15E−09	ARSD
ENSG00000106078	1.171724535	4.43E−10	2.27E−09	COBL
ENSG00000169248	0.790213883	5.21E−10	2.65E−09	CXCL11
ENSG00000143127	0.858887046	5.91E−10	2.98E−09	ITGA10
ENSG00000230487	0.635330515	7.29E−05	0.000200601	PSMG3-AS1
ENSG00000169231	0.71986648	6.66E−10	3.36E−09	THBS3
ENSG00000264112	0.630435915	3.88E−13	2.61E−12	AC015813.1
ENSG00000272512	0.629649183	0.00016484	0.000430322	AL645608.8
ENSG00000248008	0.626222703	1.44E−15	1.16E−14	NRAV
ENSG00000165698	1.042720757	1.01E−09	4.98E−09	SPACA9
ENSG00000167065	0.691874401	1.17E−09	5.78E−09	DUSP18
ENSG00000283160	0.62478041	0.003658265	0.007489349	MIR4521
ENSG00000167306	0.684016305	1.74E−09	8.41E−09	MYO5B
ENSG00000187987	1.030707676	1.80E−09	8.67E−09	ZSCAN23
ENSG00000185386	0.750119972	1.89E−09	9.10E−09	MAPK11
ENSG00000281026	0.623674242	0.00174105	0.003777549	N4BP2L2-IT2
ENSG00000167895	1.301863777	1.94E−09	9.32E−09	TMC8
ENSG00000270640	0.621162265	0.003395331	0.006999073	AC104695.3
ENSG00000263155	1.349972416	2.25E−09	1.07E−08	MYZAP
ENSG00000125637	0.809041824	2.30E−09	1.10E−08	PSD4
ENSG00000178531	0.6403658	2.47E−09	1.18E−08	CTXN1
ENSG00000006210	1.386770722	3.03E−09	1.43E−08	CX3CL1
ENSG00000196476	0.703193796	3.10E−09	1.46E−08	C20orf96
ENSG00000114841	0.724964893	3.49E−09	1.63E−08	DNAH1
ENSG00000248690	0.616225449	0.005847093	0.011483426	HAS2-AS1
ENSG00000128849	0.993028568	3.72E−09	1.74E−08	CGNL1
ENSG00000250903	0.616173957	7.24E−15	5.56E−14	GMDS-AS1
ENSG00000139192	0.677957048	4.54E−09	2.10E−08	TAPBPL
ENSG00000273888	0.615241762	0.000253027	0.00064005	FRMD6-AS1
ENSG00000276116	0.614111505	8.00E−05	0.000218844	FUT8-AS1
ENSG00000078114	0.779192836	5.73E−09	2.63E−08	NEBL
ENSG00000223745	0.609457977	6.90E−08	2.79E−07	CCDC18-AS1
ENSG00000276476	0.595189913	3.36E−05	9.71E−05	LINC00540
ENSG00000104332	1.024730115	6.35E−09	2.90E−08	SFRP1
ENSG00000187688	0.74390243	6.40E−09	2.92E−08	TRPV2
ENSG00000168961	1.249308012	7.43E−09	3.37E−08	LGALS9
ENSG00000181444	1.289743152	7.97E−09	3.60E−08	ZNF467
ENSG00000007516	0.7190471	9.06E−09	4.06E−08	BAIAP3
ENSG00000074964	0.723682951	9.90E−09	4.42E−08	ARHGEF10L
ENSG00000153233	0.860394698	1.06E−08	4.73E−08	PTPRR
ENSG00000158125	0.749227047	1.34E−08	5.87E−08	XDH
ENSG00000105982	0.809043212	1.50E−08	6.57E−08	RNF32
ENSG00000130751	0.996208061	2.12E−08	9.07E−08	NPAS1
ENSG00000187583	0.628689467	2.15E−08	9.21E−08	PLEKHN1
ENSG00000129295	0.804174666	3.78E−08	1.58E−07	LRRC6
ENSG00000136167	0.72905745	4.42E−08	1.83E−07	LCP1
ENSG00000109107	0.624763894	4.83E−08	1.99E−07	ALDOC
ENSG00000102796	0.580283788	4.92E−08	2.02E−07	DHRS12
ENSG00000169926	0.738403193	5.42E−08	2.22E−07	KLF13
ENSG00000169583	0.867293251	6.71E−08	2.72E−07	CLIC3
ENSG00000105204	0.693379327	6.79E−08	2.75E−07	DYRK1B
ENSG00000089127	0.795662065	8.09E−08	3.24E−07	OAS1
ENSG00000169220	0.610978587	8.83E−08	3.53E−07	RGS14
ENSG00000160190	0.656350516	9.89E−08	3.94E−07	SLC37A1
ENSG00000156218	0.750817532	1.07E−07	4.25E−07	ADAMTSL3
ENSG00000105854	0.64320919	1.18E−07	4.64E−07	PON2
ENSG00000103034	0.978032576	1.35E−07	5.28E−07	NDRG4
ENSG00000008517	0.607365229	1.35E−07	5.29E−07	IL32
ENSG00000163617	0.881935404	1.36E−07	5.33E−07	CCDC191
ENSG00000184194	0.756086964	1.44E−07	5.61E−07	GPR173
ENSG00000124116	0.704374738	1.47E−07	5.72E−07	WFDC3
ENSG00000197279	0.61212415	1.48E−07	5.74E−07	ZNF165
ENSG00000155265	1.06380425	1.92E−07	7.39E−07	GOLGA7B
ENSG00000196420	0.668634419	2.03E−07	7.76E−07	S100A5
ENSG00000163995	0.914334457	2.21E−07	8.44E−07	ABLIM2
ENSG00000173267	0.875959067	2.32E−07	8.85E−07	SNCG
ENSG00000174951	0.583579922	2.48E−07	9.40E−07	FUT1
ENSG00000166578	0.59325713	2.97E−07	1.12E−06	IQCD
ENSG00000134030	0.721745796	3.68E−07	1.36E−06	CTIF
ENSG00000133640	0.867286671	3.91E−07	1.45E−06	LRRIQ1
ENSG00000262576	0.845341331	4.37E−07	1.60E−06	PCDHGA4
ENSG00000160781	0.937385076	4.56E−07	1.67E−06	PAQR6
ENSG00000050165	0.701579384	5.28E−07	1.92E−06	DKK3
ENSG00000111863	0.721347277	6.34E−07	2.29E−06	ADTRP
ENSG00000160325	0.946677553	6.89E−07	2.48E−06	CACFD1
ENSG00000198846	0.743926359	7.19E−07	2.58E−06	TOX
ENSG00000166262	1.011056155	7.77E−07	2.77E−06	FAM227B
ENSG00000239282	0.740685304	9.03E−07	3.20E−06	CASTOR1
ENSG00000122547	0.589219386	1.04E−06	3.67E−06	EEPD1
ENSG00000115556	0.759404536	1.29E−06	4.50E−06	PLCD4
ENSG00000146021	0.996087722	1.42E−06	4.94E−06	KLHL3
ENSG00000167554	0.801922519	1.58E−06	5.45E−06	ZNF610
ENSG00000159899	0.607690901	1.59E−06	5.47E−06	NPR2
ENSG00000085831	0.88967755	1.86E−06	6.33E−06	TTC39A
ENSG00000167600	0.949538739	1.95E−06	6.64E−06	CYP2S1
ENSG00000135298	0.65393669	2.08E−06	7.03E−06	ADGRB3
ENSG00000185345	1.087332962	2.19E−06	7.38E−06	PRKN
ENSG00000079974	0.704729252	2.33E−06	7.83E−06	RABL2B
ENSG00000213085	0.908492786	2.67E−06	8.89E−06	CFAP45
ENSG00000137261	1.001309402	4.72E−06	1.53E−05	KIAA0319
ENSG00000131620	0.893864552	5.41E−06	1.74E−05	ANO1
ENSG00000005379	0.634150616	5.44E−06	1.75E−05	TSPOAP1
ENSG00000170927	1.061916014	5.77E−06	1.85E−05	PKHD1
ENSG00000183638	0.95696744	5.87E−06	1.88E−05	RP1L1
ENSG00000185634	0.655532658	6.41E−06	2.04E−05	SHC4
ENSG00000178026	0.704425401	7.44E−06	2.35E−05	LRRC75B
ENSG00000007237	0.94018185	7.53E−06	2.38E−05	GAS7
ENSG00000063438	0.850087695	7.99E−06	2.52E−05	AHRR
ENSG00000078081	0.943742842	1.00E−05	3.13E−05	LAMP3
ENSG00000152779	0.893034503	1.16E−05	3.58E−05	SLC16A12
ENSG00000105649	0.629673856	1.17E−05	3.59E−05	RAB3A
ENSG00000101670	0.641918014	1.74E−05	5.23E−05	LIPG
ENSG00000153246	0.640475	2.34E−05	6.95E−05	PLA2R1
ENSG00000197013	0.895543676	2.42E−05	7.15E−05	ZNF429
ENSG00000100027	0.703475726	2.55E−05	7.54E−05	YPEL1
ENSG00000222009	1.003174533	2.78E−05	8.16E−05	BTBD19
ENSG00000077092	0.583904079	2.82E−05	8.25E−05	RARB
ENSG00000138271	0.879042367	2.88E−05	8.41E−05	GPR87
ENSG00000215788	0.630842347	4.98E−05	0.000140178	TNFRSF25
ENSG00000111907	0.610094964	5.19E−05	0.000145846	TPD52L1
ENSG00000148225	0.603765317	5.24E−05	0.000146979	WDR31
ENSG00000265190	0.796463642	5.25E−05	0.00014743	ANXA8
ENSG00000166780	0.609717829	5.44E−05	0.00015227	C16orf45
ENSG00000196917	0.687020342	5.62E−05	0.000157013	HCAR1
ENSG00000243749	0.682870409	6.23E−05	0.00017307	TMEM35B
ENSG00000100100	0.614989243	6.46E−05	0.000179064	PIK3IP1
ENSG00000185261	0.808099048	6.60E−05	0.000182782	KIAA0825
ENSG00000176714	0.719708594	6.65E−05	0.000183929	CCDC121
ENSG00000189350	0.682674219	8.27E−05	0.00022538	TOGARAM2
ENSG00000117586	0.66232365	9.60E−05	0.000259115	TNFSF4
ENSG00000163283	0.702973737	9.69E−05	0.000261336	ALPP
ENSG00000100628	0.840396776	0.000102783	0.000275833	ASB2
ENSG00000204936	0.799723751	0.000132176	0.000349991	CD177
ENSG00000249242	0.674349922	0.00013392	0.000354394	TMEM150C
ENSG00000154589	0.621650835	0.000154203	0.000405048	LY96
ENSG00000137103	0.813157861	0.000158856	0.000416188	TMEM8B
ENSG00000186417	0.845874331	0.000174472	0.000454112	GLDN
ENSG00000168702	0.829015848	0.000183578	0.000476395	LRP1B
ENSG00000177694	0.678072111	0.000201099	0.000518177	NAALADL2
ENSG00000137460	0.621505207	0.000215133	0.000551848	FHDC1
ENSG00000233493	0.606768387	0.000304964	0.000759001	TMEM238
ENSG00000135525	0.592393732	0.000307346	0.00076377	MAP7
ENSG00000177076	0.595537696	0.000354308	0.000869936	ACER2
ENSG00000172456	0.624018639	0.000361318	0.000885657	FGGY
ENSG00000112303	0.791611125	0.000395261	0.00096382	VNN2
ENSG00000174327	0.660320887	0.000409421	0.000994655	SLC16A13
ENSG00000118997	0.801710893	0.000462226	0.001115511	DNAH7
ENSG00000255690	0.769175757	0.000506713	0.001215944	TRIL
ENSG00000010295	0.685112629	0.000525958	0.001259364	IFFO1
ENSG00000145107	0.585678306	0.000567754	0.001349109	TM4SF19
ENSG00000168026	0.683159923	0.000690836	0.001616374	TTC21A
ENSG00000137285	0.737035799	0.000813426	0.00187585	TUBB2B
ENSG00000156510	0.685659195	0.000879493	0.002015241	HKDC1
ENSG00000153237	0.729462631	0.000921302	0.002105764	CCDC148
ENSG00000131849	0.669939869	0.001076571	0.002430196	ZNF132
ENSG00000169550	0.724738148	0.0011291	0.002537865	MUC15
ENSG00000253649	0.611969408	0.001576863	0.003452116	PRSS51
ENSG00000264230	0.647657643	0.001653031	0.003600257	ANXA8L1
ENSG00000183401	0.685190181	0.002421385	0.005126606	CCDC159
ENSG00000166816	0.611266978	0.00242422	0.005130953	LDHD
ENSG00000108932	0.615640297	0.002572756	0.00541913	SLC16A6
ENSG00000106125	0.593529032	0.003116381	0.006461581	MINDY4
ENSG00000183091	0.60347346	0.003149988	0.006521995	NEB
ENSG00000152582	0.655526014	0.004260382	0.008617234	SPEF2

**Table 3:** Genes downregulated in *LOXL2* knockdown

Locus	logFC	*P*-Value	FDR	Gene name
ENSG00000134013	−3.053434126	0	0	LOXL2
ENSG00000122545	−1.493781116	6.6E−272	1.45E−268	SEP-T7
ENSG00000137801	−1.65563595	1.11E−262	2.08E−259	THBS1
ENSG00000112062	−1.367605967	1.43E−252	2.34E−249	MAPK14
ENSG00000146281	−1.644281336	6.67E−244	9.73E−241	PM20D2
ENSG00000105971	−1.358844865	4.29E−189	3.31E−186	CAV2
ENSG00000117500	−1.227263597	2.54E−176	1.67E−173	TMED5
ENSG00000172380	−1.135404374	1.81E−162	1.08E−159	GNG12
ENSG00000152558	−1.10232016	1.28E−160	7.33E−158	TMEM123
ENSG00000100462	−1.134694357	2.31E−160	1.26E−157	PRMT5
ENSG00000213281	−1.098589526	6.65E−160	3.49E−157	NRAS
ENSG00000162521	−1.195180927	7.41E−138	2.95E−135	RBBP4
ENSG00000196396	−1.038088551	1.5E−136	5.64E−134	PTPN1
ENSG00000182400	−1.288279943	9.25E−134	3.2E−131	TRAPPC6B
ENSG00000115339	−1.421874178	1.01E−132	3.31E−130	GALNT3
ENSG00000105810	−1.03878264	1.67E−132	5.35E−130	CDK6
ENSG00000105849	−1.177181646	7.96E−125	2.38E−122	TWISTNB
ENSG00000176853	−0.991116504	4.73E−124	1.38E−121	FAM91A1
ENSG00000113742	−1.271922708	3.08E−120	8.43E−118	CPEB4
ENSG00000156017	−1.215741594	1.39E−117	3.52E−115	CARNMT1
ENSG00000154429	−1.346899288	2.62E−117	6.5E−115	CCSAP
ENSG00000101974	−1.060939839	1.68E−116	4.02E−114	ATP11C
ENSG00000073712	−0.991731657	6.59E−116	1.54E−113	FERMT2
ENSG00000162104	−1.444767007	1.64E−113	3.77E−111	ADCY9
ENSG00000064042	−1.111524916	1.06E−110	2.36E−108	LIMCH1
ENSG00000087086	−0.890490359	4.05E−108	8.57E−106	FTL
ENSG00000106460	−1.091704144	6.11E−108	1.27E−105	TMEM106B
ENSG00000095752	−1.227515654	8.77E−108	1.8E−105	IL11
ENSG00000135521	−1.168276042	5.89E−106	1.12E−103	LTV1
ENSG00000187908	−1.211596715	7.57E−106	1.42E−103	DMBT1
ENSG00000176788	−1.162731668	1.53E−103	2.82E−101	BASP1
ENSG00000104375	−1.261014996	2.57E−102	4.69E−100	STK3
ENSG00000146143	−1.074303191	9.78E−102	1.76E−99	PRIM2
ENSG00000145284	−1.323720519	4.77E−100	8.34E−98	SCD5
ENSG00000113645	−0.916653973	2.11E−99	3.65E−97	WWC1
ENSG00000163161	−1.018873601	3.41E−97	5.59E−95	ERCC3
ENSG00000184007	−0.822555541	1.26E−96	2.02E−94	PTP4A2
ENSG00000143977	−0.987888082	2.7E−93	4.07E−91	SNRPG
ENSG00000141994	−1.367808305	2.61E−92	3.9E−90	DUS3L
ENSG00000172954	−1.050710392	5.52E−92	8.14E−90	LCLAT1
ENSG00000180694	−1.033774023	2.99E−91	4.36E−89	TMEM64
ENSG00000101003	−1.071829328	3.19E−89	4.36E−87	GINS1
ENSG00000123689	−0.87877304	8.1E−88	1.07E−85	G0S2
ENSG00000108561	−0.906168536	7.24E−87	9.51E−85	C1QBP
ENSG00000175348	−1.039373012	2.22E−85	2.75E−83	TMEM9B
ENSG00000011201	−1.309694851	3.72E−85	4.57E−83	ANOS1
ENSG00000097033	−0.780495141	1.21E−84	1.46E−82	SH3GLB1
ENSG00000103495	−0.812954992	2.8E−83	3.28E−81	MAZ
ENSG00000109084	−1.036628155	7.56E−83	8.79E−81	TMEM97
ENSG00000170248	−0.756074633	1.33E−82	1.53E−80	PDCD6IP
ENSG00000154734	−0.993464022	2.81E−80	3.07E−78	ADAMTS1
ENSG00000198959	−0.78388823	3.88E−80	4.17E−78	TGM2
ENSG00000109270	−1.102716006	5.57E−79	5.9E−77	LAMTOR3
ENSG00000171033	−1.511641065	3.88E−78	4.07E−76	PKIA
ENSG00000262919	−1.286604883	1.12E−77	1.16E−75	FAM58A
ENSG00000076003	−0.895743892	1.38E−76	1.39E−74	MCM6
ENSG00000104164	−0.880674477	1.48E−76	1.47E−74	BLOC1S6
ENSG00000112149	−1.349898585	1.17E−75	1.15E−73	CD83
ENSG00000173110	−1.621696267	2.04E−75	1.97E−73	HSPA6
ENSG00000196865	–1.052977006	1.1E−74	1.06E−72	NHLRC2
ENSG00000164209	–0.79794111	1.25E−74	1.19E−72	SLC25A46
ENSG00000092853	–0.745590308	2.04E−73	1.89E−71	CLSPN
ENSG00000163513	–0.719014341	2.71E−73	2.48E−71	TGFBR2
ENSG00000127314	–0.959251127	2.17E−72	1.93E−70	RAP1B
ENSG00000005020	–0.909609789	2.38E−71	2.04E−69	SKAP2
ENSG00000003989	–1.062977014	3.54E−71	3.02E−69	SLC7A2
ENSG00000076248	–0.818890935	7.73E−70	6.43E−68	UNG
ENSG00000110104	–0.886356935	8.21E−70	6.74E−68	CCDC86
ENSG00000106034	-2.258112177	2.58E−69	2.1E−67	CPED1
ENSG00000139793	–0.822257564	3.6E−69	2.92E−67	MBNL2
ENSG00000122870	–1.265770623	1.9E−68	1.5E−66	BICC1
ENSG00000188811	–1.270791643	4.79E−68	3.75E−66	NHLRC3
ENSG00000140262	–0.845128144	4.91E−68	3.81E−66	TCF12
ENSG00000171791	–1.383509833	6.73E−68	5.2E−66	BCL2
ENSG00000085449	–0.807391186	7.7E−68	5.92E−66	WDFY1
ENSG00000163527	–0.680451793	8.38E−68	6.36E−66	STT3B
ENSG00000101856	–0.725495533	9.47E−68	7.15E−66	PGRMC1
ENSG00000198689	–0.855931203	1.38E−67	1.04E−65	SLC9A6
ENSG00000139921	–0.707226796	1.81E−67	1.35E−65	TMX1
ENSG00000146047	–0.927456112	2.17E−67	1.61E−65	HIST1H2BA
ENSG00000171867	–0.693918536	2.33E−67	1.72E−65	PRNP
ENSG00000100625	–0.866022602	5.2E−67	3.79E−65	SIX4
ENSG00000160208	–0.734399871	3.02E−66	2.15E−64	RRP1B
ENSG00000153132	–0.846027423	3.16E−66	2.24E−64	CLGN
ENSG00000151151	–1.049984787	3.44E−66	2.43E−64	IPMK
ENSG00000198948	–1.324799997	4.2E−66	2.95E−64	MFAP3L
ENSG00000213160	–1.153831739	4.97E−66	3.45E−64	KLHL23
ENSG00000168615	–0.756543804	7.94E−66	5.48E−64	ADAM9
ENSG00000198964	–0.81917694	1.48E−65	1.02E−63	SGMS1
ENSG00000111371	–0.655358043	6.25E−65	4.28E−63	SLC38A1
ENSG00000138756	–0.727021119	3.81E−64	2.55E−62	BMP2K
ENSG00000274997	–0.669796522	2.01E−63	1.33E−61	HIST1H2AH
ENSG00000166128	–0.743923809	3.52E−63	2.29E−61	RAB8B
ENSG00000153130	–0.71036132	7.22E−63	4.65E−61	SCOC
ENSG00000058056	–0.791211898	9.57E−63	6.07E−61	USP13
ENSG00000183598	–0.937984455	1.22E−62	7.72E−61	HIST2H3D
ENSG00000157657	–1.331037104	6.49E−62	4.06E−60	ZNF618
ENSG00000109586	–0.806715459	3.03E−61	1.89E−59	GALNT7
ENSG00000144354	–1.284264102	8.96E−61	5.55E−59	CDCA7
ENSG00000164211	–0.821415196	9.8E−61	6.04E−59	STARD4
ENSG00000099901	–0.797049931	1.04E−60	6.37E−59	RANBP1
ENSG00000103342	–0.665272247	1.86E−60	1.13E−58	GSPT1
ENSG00000273703	–0.759808174	1.89E−60	1.14E−58	HIST1H2BM
ENSG00000162613	–0.785667152	3.15E−60	1.88E−58	FUBP1
ENSG00000156802	–0.643909783	3.42E−60	2.03E−58	ATAD2
ENSG00000107854	–0.712659437	8.75E−60	5.13E−58	TNKS2
ENSG00000030304	–1.71173144	1.31E−59	7.55E−58	MUSK
ENSG00000264364	–0.726395847	2.1E−59	1.19E−57	DYNLL2
ENSG00000131016	–0.791953337	2.29E−59	1.29E−57	AKAP12
ENSG00000115364	–0.778545058	2.65E−59	1.48E−57	MRPL19
ENSG00000111666	–0.804368606	8.4E−59	4.64E−57	CHPT1
ENSG00000106771	–0.644642299	1.24E−58	6.77E−57	TMEM245
ENSG00000154553	-2.010549714	1.69E−58	9.16E−57	PDLIM3
ENSG00000180957	–0.842836946	2.11E−58	1.14E−56	PITPNB
ENSG00000164081	–0.810106876	4.81E−58	2.58E−56	TEX264
ENSG00000112118	–0.639768587	5.17E−58	2.75E−56	MCM3
ENSG00000189057	–0.826299148	7.77E−58	4.09E−56	FAM111B
ENSG00000116095	–0.885698839	4.64E−57	2.43E−55	PLEKHA3
ENSG00000113083	–1.085829177	4.8E−57	2.5E−55	LOX
ENSG00000165156	–0.739479342	5.04E−57	2.62E−55	ZHX1
ENSG00000113448	–0.76465591	5.39E−57	2.79E−55	PDE4D
ENSG00000166260	–0.917921133	1.07E−56	5.44E−55	COX11
ENSG00000203668	–0.623298669	1.52E−56	7.69E−55	CHML
ENSG00000105698	–0.737929868	1.71E−56	8.62E−55	USF2
ENSG00000176890	–0.736138571	2.01E−56	1.01E−54	TYMS
ENSG00000164466	–0.68314086	2.1E−56	1.05E−54	SFXN1
ENSG00000197312	–0.84172031	7.43E−56	3.67E−54	DDI2
ENSG00000132581	–0.797529275	1.08E−55	5.31E−54	SDF2
ENSG00000105281	–0.76012066	1.34E−54	6.44E−53	SLC1A5
ENSG00000101773	–0.684417581	1.43E−54	6.8E−53	RBBP8
ENSG00000125166	–0.64746293	2.47E−54	1.17E−52	GOT2
ENSG00000065615	–0.766061917	3.65E−54	1.72E−52	CYB5R4
ENSG00000106366	–0.690600823	4.53E−54	2.12E−52	SERPINE1
ENSG00000138675	–1.055137884	2.97E−53	1.37E−51	FGF5
ENSG00000166801	–0.679971393	4.81E−53	2.18E−51	FAM111A
ENSG00000125430	–1.119040342	1.8E−52	8.13E−51	HS3ST3B1
ENSG00000136986	–0.64205774	3.04E−52	1.37E−50	DERL1
ENSG00000143507	–0.824435143	3.91E−52	1.75E−50	DUSP10
ENSG00000132646	–0.659226998	4.16E−52	1.85E−50	PCNA
ENSG00000092470	–0.776503363	4.36E−52	1.93E−50	WDR76
ENSG00000125870	–0.692582004	6.21E−52	2.72E−50	SNRPB2
ENSG00000130830	–1.001331439	7.01E−52	3.05E−50	MPP1
ENSG00000169193	–1.233765255	1.18E−51	5.09E−50	CCDC126
ENSG00000115159	–0.745203813	1.55E−51	6.63E−50	GPD2
ENSG00000089597	–0.608914143	4.59E−51	1.95E−49	GANAB
ENSG00000138448	–0.679300714	2.25E−50	9.46E−49	ITGAV
ENSG00000261609	–0.838562302	3.25E−50	1.36E−48	GAN
ENSG00000118596	–0.91558522	3.25E−50	1.36E−48	SLC16A7
ENSG00000104388	–0.77698668	8.4E−50	3.46E−48	RAB2A
ENSG00000111725	–0.851677774	1.59E−49	6.47E−48	PRKAB1
ENSG00000070214	–0.695894872	1.61E−49	6.52E−48	SLC44A1
ENSG00000149289	–0.717233099	1.62E−49	6.53E−48	ZC3H12C
ENSG00000196323	–0.700131838	1.75E−49	7.04E−48	ZBTB44
ENSG00000182504	–0.657051624	8.72E−49	3.46E−47	CEP97
ENSG00000082269	–0.721562816	2.1E−48	8.21E−47	FAM135A
ENSG00000055208	–0.688892188	2.44E−48	9.51E−47	TAB2
ENSG00000083720	–0.85708639	3.3E−48	1.28E−46	OXCT1
ENSG00000110031	–0.750834355	3.95E−48	1.53E−46	LPXN
ENSG00000121966	–0.877794416	4.44E−48	1.72E−46	CXCR4
ENSG00000081923	–0.788155108	8.6E−48	3.3E−46	ATP8B1
ENSG00000133026	–0.759331662	3.21E−47	1.21E−45	MYH10
ENSG00000167645	–0.945756835	3.93E−47	1.47E−45	YIF1B
ENSG00000048392	–0.69447929	5.19E−47	1.93E−45	RRM2B
ENSG00000157978	–0.881827291	9.73E−47	3.61E−45	LDLRAP1
ENSG00000169429	–0.814790283	1.09E−46	4.01E−45	CXCL8
ENSG00000253304	–1.283748113	2.32E−46	8.48E−45	TMEM200B
ENSG00000139278	–0.735325809	3.22E−46	1.18E−44	GLIPR1
ENSG00000164070	–0.61500254	3.29E−46	1.2E−44	HSPA4L
ENSG00000116406	–0.582479865	3.37E−46	1.22E−44	EDEM3
ENSG00000205302	–0.632567484	6.01E−46	2.18E−44	SNX2
ENSG00000119917	–0.598753864	1.72E−45	6.12E−44	IFIT3
ENSG00000094804	–0.61234381	2.51E−45	8.93E−44	CDC6
ENSG00000180998	–1.39647951	2.96E−45	1.05E−43	GPR137C
ENSG00000166741	–1.457281718	1.07E−44	3.74E−43	NNMT
ENSG00000113070	–0.759564729	1.28E−44	4.43E−43	HBEGF
ENSG00000135250	–0.766365183	1.37E−44	4.74E−43	SRPK2
ENSG00000106683	–0.770624188	2.57E−44	8.9E−43	LIMK1
ENSG00000214517	–0.683773756	2.59E−44	8.93E−43	PPME1
ENSG00000100479	–0.857727695	2.8E−44	9.64E−43	POLE2
ENSG00000130175	–0.636724289	5.94E−44	2.02E−42	PRKCSH
ENSG00000170185	–0.607338959	1.22E−43	4.08E−42	USP38
ENSG00000123908	–0.609276107	1.75E−43	5.86E−42	37469
ENSG00000064666	–0.604102394	1.93E−43	6.43E−42	CNN2
ENSG00000130024	–0.624632149	4.42E−43	1.45E−41	PHF10
ENSG00000135272	–0.779876332	1.35E−42	4.34E−41	MDFIC
ENSG00000138646	–0.611105347	1.87E−42	5.96E−41	HERC5
ENSG00000178904	–0.821536016	4.05E−42	1.28E−40	DPY19L3
ENSG00000116984	–0.592452679	6.84E−42	2.15E−40	MTR
ENSG00000151233	–0.696734208	1.24E−41	3.89E−40	GXYLT1
ENSG00000049130	–1.363755139	2.4E−41	7.47E−40	KITLG
ENSG00000131943	–0.819671981	4.72E−41	1.45E−39	C19orf12
ENSG00000180730	–0.972346863	6.99E−41	2.14E−39	SHISA2
ENSG00000151239	–0.646933517	1.79E−40	5.44E−39	TWF1
ENSG00000132819	–0.768136766	2.14E−40	6.44E−39	RBM38
ENSG00000163577	–0.691799144	2.24E−40	6.7E−39	EIF5A2
ENSG00000145743	–0.871378383	2.77E−40	8.28E−39	FBXL17
ENSG00000137168	–0.580135569	9.42E−40	2.77E−38	PPIL1
ENSG00000038210	–0.645756993	1.75E−39	5.06E−38	PI4K2B
ENSG00000146263	–0.647666403	1.86E−39	5.35E−38	MMS22L
ENSG00000116717	–0.675446031	1.93E−39	5.56E−38	GADD45A
ENSG00000167842	–0.621928208	1.97E−39	5.64E−38	MIS12
ENSG00000114520	–0.763326165	2.34E−39	6.7E−38	SNX4
ENSG00000154678	–0.944300821	2.5E−39	7.13E−38	PDE1C
ENSG00000164402	–0.637231639	4.65E−39	1.31E−37	39692
ENSG00000162073	–0.831857412	5.7E−39	1.6E−37	PAQR4
ENSG00000144369	–0.757917112	8.73E−39	2.43E−37	FAM171B
ENSG00000123572	–1.320658507	8.85E−39	2.46E−37	NRK
ENSG00000129691	–0.662466725	1.08E−38	2.99E−37	ASH2L
ENSG00000002822	–0.607342874	1.85E−38	5.06E−37	MAD1L1
ENSG00000101412	–0.797853061	2.45E−38	6.64E−37	E2F1
ENSG00000156162	–0.585388505	2.98E−38	8.02E−37	DPY19L4
ENSG00000112144	–0.601617859	3.85E−38	1.03E−36	ICK
ENSG00000125734	–0.622236713	3.88E−38	1.04E−36	GPR108
ENSG00000101544	–0.625762283	3.98E−38	1.06E−36	ADNP2
ENSG00000198860	–0.623117034	4.95E−38	1.31E−36	TSEN15
ENSG00000091986	–0.628100732	5.14E−38	1.36E−36	CCDC80
ENSG00000114982	–0.612077259	9.28E−38	2.42E−36	KANSL3
ENSG00000145604	–0.706553217	1.28E−37	3.32E−36	SKP2
ENSG00000187051	–0.687870702	1.56E−37	4.04E−36	RPS19BP1
ENSG00000163626	–0.701696175	2.3E−37	5.91E−36	COX18
ENSG00000125885	–0.583920903	2.3E−37	5.91E−36	MCM8
ENSG00000157557	–0.662994439	4.57E−37	1.15E−35	ETS2
ENSG00000127564	–0.830827192	4.99E−37	1.26E−35	PKMYT1
ENSG00000172260	–0.882667662	5.77E−37	1.45E−35	NEGR1
ENSG00000198478	–0.913187859	7.59E−37	1.9E−35	SH3BGRL2
ENSG00000152455	–0.651372389	9.5E−37	2.37E−35	SUV39H2
ENSG00000181744	–0.857381192	3.74E−36	9.25E−35	C3orf58
ENSG00000105825	–0.683206742	4.19E−36	1.03E−34	TFPI2
ENSG00000185129	–0.650813837	4.66E−36	1.14E−34	PURA
ENSG00000131153	–0.830497048	5.14E−36	1.26E−34	GINS2
ENSG00000104361	–1.128506294	9.17E−36	2.23E−34	NIPAL2
ENSG00000142552	-2.164888397	1.32E−35	3.17E−34	RCN3
ENSG00000118971	–0.828403671	1.65E−35	3.94E−34	CCND2
ENSG00000162437	–0.911533859	1.86E−35	4.42E−34	RAVER2
ENSG00000072609	–0.725569758	2.69E−35	6.35E−34	CHFR
ENSG00000135750	–1.097089858	2.74E−35	6.47E−34	KCNK1
ENSG00000198108	–1.330709511	3.6E−35	8.47E−34	CHSY3
ENSG00000139438	–1.36818776	5.27E−35	1.23E−33	FAM222A
ENSG00000176170	–1.061371315	1.42E−34	3.25E−33	SPHK1
ENSG00000172159	–0.719708766	1.54E−34	3.53E−33	FRMD3
ENSG00000115380	–0.592899689	2.06E−34	4.68E−33	EFEMP1
ENSG00000109511	–1.823866992	2.43E−34	5.52E−33	ANXA10
ENSG00000198780	–0.894526335	2.96E−34	6.7E−33	FAM169A
ENSG00000132970	–0.851435716	1.4E−33	3.08E−32	WASF3
ENSG00000136111	–0.603181977	1.96E−33	4.27E−32	TBC1D4
ENSG00000225830	–0.709999482	2.48E−33	5.35E−32	ERCC6
ENSG00000109255	–1.175751743	3.49E−33	7.47E−32	NMU
ENSG00000121058	–0.618925976	4.93E−33	1.05E−31	COIL
ENSG00000164508	–1.194336423	7.79E−33	1.64E−31	HIST1H2AA
ENSG00000123570	–1.292915486	7.81E−33	1.65E−31	RAB9B
ENSG00000176834	–0.652497435	8.39E−33	1.76E−31	VSIG10
ENSG00000186416	–0.589099004	8.99E−33	1.89E−31	NKRF
ENSG00000106789	–0.67263713	1.3E−32	2.72E−31	CORO2A
ENSG00000172379	–0.788111039	1.74E−32	3.62E−31	ARNT2
ENSG00000112592	–0.651455796	1.74E−32	3.62E−31	TBP
ENSG00000159259	–0.683175408	3.54E−32	7.29E−31	CHAF1B
ENSG00000226742	–1.146129313	6.12E−32	1.24E−30	HSBP1L1
ENSG00000129173	–0.786345274	7.03E−32	1.43E−30	E2F8
ENSG00000170689	–0.891856708	1.2E−31	2.41E−30	HOXB9
ENSG00000112294	–1.176881508	1.51E−31	2.99E−30	ALDH5A1
ENSG00000174021	–0.597920622	2E−31	3.95E−30	GNG5
ENSG00000164199	–0.796504879	2.27E−31	4.46E−30	ADGRV1
ENSG00000153037	–0.672607947	3.19E−31	6.24E−30	SRP19
ENSG00000170734	–0.586607195	3.55E−31	6.92E−30	POLH
ENSG00000179454	–0.750172183	3.78E−31	7.35E−30	KLHL28
ENSG00000168564	–0.588203808	4.93E−31	9.54E−30	CDKN2AIP
ENSG00000112031	–0.762313636	6.71E−31	1.29E−29	MTRF1L
ENSG00000180773	–0.580924737	8.38E−31	1.61E−29	SLC36A4
ENSG00000164161	–1.167949712	8.5E−31	1.63E−29	HHIP
ENSG00000170085	–1.010340626	8.52E−31	1.63E−29	SIMC1
ENSG00000213047	–0.852741909	9.45E−31	1.8E−29	DENND1B
ENSG00000178695	–0.608721797	1.3E−30	2.45E−29	KCTD12
ENSG00000162694	–0.581927567	1.4E−30	2.63E−29	EXTL2
ENSG00000153993	–1.881252549	2.69E−30	5.02E−29	SEMA3D
ENSG00000121236	–0.974421588	3.3E−30	6.14E−29	TRIM6
ENSG00000157869	–0.655287956	4.29E−30	7.95E−29	RAB28
ENSG00000135966	–0.618380605	6.88E−30	1.26E−28	TGFBRAP1
ENSG00000121005	–1.212412618	8.16E−30	1.49E−28	CRISPLD1
ENSG00000067177	–0.643679511	1.08E−29	1.95E−28	PHKA1
ENSG00000104643	–0.792913093	1.57E−29	2.8E−28	MTMR9
ENSG00000163818	–0.711190839	1.7E−29	3.03E−28	LZTFL1
ENSG00000145545	–0.582387628	2.79E−29	4.91E−28	SRD5A1
ENSG00000143376	–0.70665512	3.54E−29	6.2E−28	SNX27
ENSG00000276043	–0.609555567	5.15E−29	8.95E−28	UHRF1
ENSG00000176406	–1.738298125	1.08E−28	1.85E−27	RIMS2
ENSG00000101871	–0.597281832	1.53E−28	2.6E−27	MID1
ENSG00000197223	–0.808372429	4.86E−28	8.12E−27	C1D
ENSG00000214357	–0.84131965	5.59E−28	9.31E−27	NEURL1B
ENSG00000137462	–1.051657592	7.74E−28	1.28E−26	TLR2
ENSG00000040199	–0.614077873	1.01E−27	1.65E−26	PHLPP2
ENSG00000135185	–0.890686199	1.23E−27	2.01E−26	TMEM243
ENSG00000128591	–0.771115601	1.28E−27	2.1E−26	FLNC
ENSG00000113356	–0.70152701	1.33E−27	2.17E−26	POLR3G
ENSG00000112218	–1.31975116	1.38E−27	2.25E−26	GPR63
ENSG00000166250	–0.584854006	1.41E−27	2.3E−26	CLMP
ENSG00000123892	–0.709596504	2.05E−27	3.31E−26	RAB38
ENSG00000126215	–0.59285666	3.95E−27	6.32E−26	XRCC3
ENSG00000111331	–0.709429851	4.04E−27	6.45E−26	OAS3
ENSG00000179348	–0.959652747	5.77E−27	9.16E−26	GATA2
ENSG00000135045	–0.580419299	7.97E−27	1.25E−25	C9orf40
ENSG00000128923	–0.640712113	9.41E−27	1.48E−25	MINDY2
ENSG00000151150	-1.00381512	1.76E−26	2.72E−25	ANK3
ENSG00000167513	–0.659977018	3.21E−26	4.9E−25	CDT1
ENSG00000084676	–0.598801314	4.32E−26	6.55E−25	NCOA1
ENSG00000174405	–0.594636053	7.09E−26	1.06E−24	LIG4
ENSG00000134056	–0.815574482	1.05E−25	1.57E−24	MRPS36
ENSG00000137965	–0.582015156	1.21E−25	1.79E−24	IFI44
ENSG00000157510	–0.701292502	1.38E−25	2.04E−24	AFAP1L1
ENSG00000173083	–0.723996154	2.26E−25	3.32E−24	HPSE
ENSG00000175197	–0.673342058	5.14E−25	7.46E−24	DDIT3
ENSG00000108960	–0.58542233	6.22E−25	8.99E−24	MMD
ENSG00000100916	–0.624568654	1.56E−24	2.22E−23	BRMS1L
ENSG00000181222	–0.726152084	1.6E−24	2.27E−23	POLR2A
ENSG00000187123	–0.956022878	2.07E−24	2.9E−23	LYPD6
ENSG00000124788	–0.590242443	2.42E−24	3.37E−23	ATXN1
ENSG00000129354	–0.691992358	2.83E−24	3.93E−23	AP1M2
ENSG00000078401	–0.592475007	4E−24	5.52E−23	EDN1
ENSG00000121316	–1.050558857	6.3E−24	8.57E−23	PLBD1
ENSG00000121211	–0.582342226	6.37E−24	8.66E−23	MND1
ENSG00000135776	–0.585970667	1.14E−23	1.52E−22	ABCB10
ENSG00000162599	–0.648028127	1.15E−23	1.53E−22	NFIA
ENSG00000143195	–0.652324164	1.34E−23	1.78E−22	ILDR2
ENSG00000182010	–0.810382882	1.94E−23	2.57E−22	RTKN2
ENSG00000196083	–0.719308824	1.98E−23	2.62E−22	IL1RAP
ENSG00000135698	–0.685340754	2.6E−23	3.41E−22	MPHOSPH6
ENSG00000122435	–0.73861782	4.38E−23	5.68E−22	TRMT13
ENSG00000099256	–0.742089516	5.07E−23	6.54E−22	PRTFDC1
ENSG00000173926	–0.725353141	5.49E−23	7.06E−22	MARCH3
ENSG00000171016	–0.635078785	6.62E−23	8.48E−22	PYGO1
ENSG00000166908	–0.610595678	1.01E−22	1.28E−21	PIP4K2C
ENSG00000254535	–1.198870344	1.2E−22	1.52E−21	PABPC4L
ENSG00000117481	–0.628980367	1.35E−22	1.69E−21	NSUN4
ENSG00000049192	–0.719589951	1.56E−22	1.96E−21	ADAMTS6
ENSG00000133739	–0.688520659	1.78E−22	2.23E−21	LRRCC1
ENSG00000083454	–1.060807972	1.96E−22	2.44E−21	P2RX5
ENSG00000154319	–0.887801464	2.23E−22	2.77E−21	FAM167A
ENSG00000023892	–0.675886174	2.38E−22	2.95E−21	DEF6
ENSG00000138795	–0.843440962	3.04E−22	3.75E−21	LEF1
ENSG00000079156	–0.842535226	3.07E−22	3.78E−21	OSBPL6
ENSG00000170396	–0.805881831	3.81E−22	4.67E−21	ZNF804A
ENSG00000135211	–0.747849147	3.86E−22	4.73E−21	TMEM60
ENSG00000165323	–0.725561816	6.07E−22	7.35E−21	FAT3
ENSG00000152495	–0.751284296	6.71E−22	8.1E−21	CAMK4
ENSG00000124813	–0.681157995	7.41E−22	8.93E−21	RUNX2
ENSG00000109738	–0.716767424	1.08E−21	1.3E−20	GLRB
ENSG00000082438	–1.400231606	1.49E−21	1.77E−20	COBLL1
ENSG00000138463	–0.692070234	1.91E−21	2.25E−20	DIRC2
ENSG00000148680	–0.6672409	1.96E−21	2.31E−20	HTR7
ENSG00000169750	–0.921289251	3.24E−21	3.75E−20	RAC3
ENSG00000114698	–1.130704953	3.84E−21	4.42E−20	PLSCR4
ENSG00000112319	–0.822410931	6.03E−21	6.89E−20	EYA4
ENSG00000115392	–0.58789192	6.16E−21	7.02E−20	FANCL
ENSG00000185818	–1.163457685	6.72E−21	7.63E−20	NAT8L
ENSG00000143942	–0.728072397	6.72E−21	7.63E−20	CHAC2
ENSG00000185862	–0.776759935	1.13E−20	1.27E−19	EVI2B
ENSG00000164619	–1.07660645	1.2E−20	1.35E−19	BMPER
ENSG00000058091	–0.700546181	2.42E−20	2.67E−19	CDK14
ENSG00000175556	–0.6405596	2.9E−20	3.19E−19	LONRF3
ENSG00000176641	–0.725354894	2.93E−20	3.22E−19	RNF152
ENSG00000135414	–0.646867873	6.7E−20	7.23E−19	GDF11
ENSG00000134569	–0.789074753	6.71E−20	7.24E−19	LRP4
ENSG00000080823	–0.602706451	7.6E−20	8.18E−19	MOK
ENSG00000188312	–0.722540228	8.34E−20	8.96E−19	CENPP
ENSG00000163568	–0.962591724	1.46E−19	1.56E−18	AIM2
ENSG00000160392	–0.610014247	2.03E−19	2.14E−18	C19orf47
ENSG00000119042	–0.612708814	3.03E−19	3.17E−18	SATB2
ENSG00000166532	–0.673619231	3.77E−19	3.91E−18	RIMKLB
ENSG00000100483	–0.677631261	8.66E−19	8.8E−18	VCPKMT
ENSG00000080493	–1.327083425	2.48E−18	2.44E−17	SLC4A4
ENSG00000248487	–0.969369054	2.57E−18	2.52E−17	ABHD14A
ENSG00000153956	–1.026853205	3.17E−18	3.09E−17	CACNA2D1
ENSG00000204767	–0.615679238	4.62E−18	4.46E−17	FAM196B
ENSG00000166582	–0.670746514	5.34E−18	5.15E−17	CENPV
ENSG00000121578	–0.646426771	5.55E−18	5.33E−17	B4GALT4
ENSG00000175183	–0.603690194	7.81E−18	7.4E−17	CSRP2
ENSG00000134508	–0.584140679	7.84E−18	7.42E−17	CABLES1
ENSG00000154237	–0.591563665	1.09E−17	1.02E−16	LRRK1
ENSG00000118257	–0.661860339	1.27E−17	1.19E−16	NRP2
ENSG00000136160	–0.728515383	1.31E−17	1.22E−16	EDNRB
ENSG00000172889	–0.78025385	2.36E−17	2.17E−16	EGFL7
ENSG00000170498	–1.543019875	2.8E−17	2.56E−16	KISS1
ENSG00000112394	–1.707112548	4.7E−17	4.24E−16	SLC16A10
ENSG00000151229	–0.746387031	6.14E−17	5.48E−16	SLC2A13
ENSG00000091129	–1.696794072	6.83E−17	6.05E−16	NRCAM
ENSG00000171388	–1.043380952	8.76E−17	7.71E−16	APLN
ENSG00000228300	–0.706210371	9.96E−17	8.73E−16	C19orf24
ENSG00000149970	–0.855419499	1.04E−16	9.09E−16	CNKSR2
ENSG00000162711	–0.776561197	1.06E−16	9.24E−16	NLRP3
ENSG00000156795	–0.622341733	1.18E−16	1.03E−15	WDYHV1
ENSG00000169684	–0.74358858	2.58E−16	2.21E−15	CHRNA5
ENSG00000197147	–0.671838185	2.76E−16	2.36E−15	LRRC8B
ENSG00000206538	–0.84083006	3.21E−16	2.72E−15	VGLL3
ENSG00000133101	–0.714667229	3.28E−16	2.78E−15	CCNA1
ENSG00000107518	–0.601853491	3.48E−16	2.94E−15	ATRNL1
ENSG00000155495	–1.359915587	4.29E−16	3.61E−15	MAGEC1
ENSG00000181754	–0.612040358	5.47E−16	4.56E−15	AMIGO1
ENSG00000168685	–0.629731176	6.4E−16	5.31E−15	IL7R
ENSG00000154274	–0.654221576	6.52E−16	5.41E−15	C4orf19
ENSG00000112425	–0.870586941	6.91E−16	5.72E−15	EPM2A
ENSG00000128610	–1.168896695	6.95E−16	5.75E−15	FEZF1
ENSG00000154914	–0.915019891	7.14E−16	5.9E−15	USP43
ENSG00000082014	–1.08393885	8.58E−16	7.05E−15	SMARCD3
ENSG00000105929	–0.876658986	3.48E−15	2.74E−14	ATP6V0A4
ENSG00000169570	–0.772019802	4.97E−15	3.88E−14	DTWD2
ENSG00000131480	–1.0578589	7.95E−15	6.09E−14	AOC2
ENSG00000127311	–0.757865784	8.99E−15	6.87E−14	HELB
ENSG00000169851	–0.775603189	9.22E−15	7.04E−14	PCDH7
ENSG00000026103	–0.72906724	1.21E−14	9.14E−14	FAS
ENSG00000235194	–0.79485048	3.52E−14	2.56E−13	PPP1R3E
ENSG00000105894	–0.782561566	3.9E−14	2.83E−13	PTN
ENSG00000162545	–1.145665693	4.19E−14	3.04E−13	CAMK2N1
ENSG00000150764	–0.704440404	4.83E−14	3.49E−13	DIXDC1
ENSG00000018236	–1.47880204	4.91E−14	3.53E−13	CNTN1
ENSG00000187231	–0.644113059	5.52E−14	3.97E−13	SESTD1
ENSG00000110852	–0.662960751	6.52E−14	4.66E−13	CLEC2B
ENSG00000170577	–0.939095059	7.26E−14	5.16E−13	SIX2
ENSG00000175893	–0.614669715	1.1E−13	7.72E−13	ZDHHC21
ENSG00000084734	–1.185795317	1.34E−13	9.33E−13	GCKR
ENSG00000166689	–0.674253903	1.62E−13	1.13E−12	PLEKHA7
ENSG00000152669	–0.704229096	1.78E−13	1.23E−12	CCNO
ENSG00000069667	–0.817249087	1.88E−13	1.3E−12	RORA
ENSG00000167941	–1.822789729	2.12E−13	1.46E−12	SOST
ENSG00000152270	–0.627826609	2.26E−13	1.55E−12	PDE3B
ENSG00000152527	–0.65848094	3.22E−13	2.19E−12	PLEKHH2
ENSG00000070759	–0.583708292	4.71E−13	3.16E−12	TESK2
ENSG00000068615	–0.935596963	4.71E−13	3.16E−12	REEP1
ENSG00000163328	–1.081666267	5.61E−13	3.75E−12	GPR155
ENSG00000005249	–0.989574304	7.85E−13	5.19E−12	PRKAR2B
ENSG00000120279	–1.086387629	8.3E−13	5.48E−12	MYCT1
ENSG00000140450	–0.633642782	8.3E−13	5.48E−12	ARRDC4
ENSG00000197415	–0.649632825	1.08E−12	7.02E−12	VEPH1
ENSG00000170417	–0.93939747	1.48E−12	9.5E−12	TMEM182
ENSG00000126803	–1.11469054	1.54E−12	9.92E−12	HSPA2
ENSG00000170899	–0.684484123	1.7E−12	1.09E−11	GSTA4
ENSG00000168077	–1.165454147	2.15E−12	1.37E−11	SCARA3
ENSG00000181418	–1.58931145	2.26E−12	1.44E−11	DDN
ENSG00000154040	–0.938234977	2.4E−12	1.52E−11	CABYR
ENSG00000188060	–1.258637511	2.57E−12	1.62E−11	RAB42
ENSG00000108852	–0.843142689	2.7E−12	1.71E−11	MPP2
ENSG00000166823	–0.986678684	4.18E−12	2.61E−11	MESP1
ENSG00000104154	–0.597478232	4.45E−12	2.77E−11	SLC30A4
ENSG00000106025	–0.593643936	6.72E−12	4.1E−11	TSPAN12
ENSG00000163472	–0.745176085	1.25E−11	7.49E−11	TMEM79
ENSG00000178343	–0.948648227	1.45E−11	8.61E−11	SHISA3
ENSG00000140044	–0.769542054	1.64E−11	9.7E−11	JDP2
ENSG00000166813	–0.590817747	1.72E−11	1.01E−10	KIF7
ENSG00000143382	–1.022866716	1.85E−11	1.09E−10	ADAMTSL4
ENSG00000280670	–0.649067763	2.08E−11	1.22E−10	CCDC163
ENSG00000143320	–0.873020398	2.19E−11	1.28E−10	CRABP2
ENSG00000090020	–0.695130894	2.54E−11	1.47E−10	SLC9A1
ENSG00000116141	–0.722567149	2.54E−11	1.48E−10	MARK1
ENSG00000165655	–0.867909098	2.67E−11	1.54E−10	ZNF503
ENSG00000128694	–0.624482372	2.9E−11	1.67E−10	OSGEPL1
ENSG00000135378	–0.665705024	4.18E−11	2.39E−10	PRRG4
ENSG00000135333	–1.203520059	4.22E−11	2.42E−10	EPHA7
ENSG00000008277	–0.724949048	4.27E−11	2.44E−10	ADAM22
ENSG00000134321	–0.688580763	4.43E−11	2.53E−10	RSAD2
ENSG00000196843	–0.607550863	4.54E−11	2.59E−10	ARID5A
ENSG00000186205	–0.637588673	4.66E−11	2.65E−10	MARC1
ENSG00000171365	–0.672055612	5.69E−11	3.21E−10	CLCN5
ENSG00000128872	–0.584078549	5.78E−11	3.26E−10	TMOD2
ENSG00000221890	–0.782195142	6.9E−11	3.84E−10	NPTXR
ENSG00000234602	–0.995961314	7.14E−11	3.97E−10	MCIDAS
ENSG00000157303	–0.96944952	7.23E−11	4.02E−10	SUSD3
ENSG00000164414	–0.591096299	7.69E−11	4.27E−10	SLC35A1
ENSG00000178401	–0.94668282	7.84E−11	4.35E−10	DNAJC22
ENSG00000139973	–0.875379862	8.35E−11	4.61E−10	SYT16
ENSG00000182118	–0.593515976	8.35E−11	4.61E−10	FAM89A
ENSG00000184254	–0.62334185	8.41E−11	4.64E−10	ALDH1A3
ENSG00000130244	–0.638701689	9.48E−11	5.2E−10	FAM98C
ENSG00000011332	–0.682979301	1.07E−10	5.86E−10	DPF1
ENSG00000151468	–0.953297034	1.3E−10	7.06E−10	CCDC3
ENSG00000162999	–1.755455161	1.6E−10	8.58E−10	DUSP19
ENSG00000116396	–0.896224276	1.67E−10	8.97E−10	KCNC4
ENSG00000105290	–0.98909701	1.99E−10	1.06E−09	APLP1
ENSG00000184838	–0.709702383	2.05E−10	1.09E−09	PRR16
ENSG00000131471	–1.508965698	2.07E−10	1.1E−09	AOC3
ENSG00000141404	–1.039495113	2.15E−10	1.14E−09	GNAL
ENSG00000125384	–1.217017506	2.82E−10	1.47E−09	PTGER2
ENSG00000061918	–0.775981343	3.62E−10	1.87E−09	GUCY1B3
ENSG00000156172	–0.844143968	3.84E−10	1.98E−09	C8orf37
ENSG00000100092	–0.612795457	5.7E−10	2.89E−09	SH3BP1
ENSG00000175175	–0.668290059	6.19E−10	3.13E−09	PPM1E
ENSG00000150893	–0.822911314	7.32E−10	3.67E−09	FREM2
ENSG00000173890	–0.725043577	9.66E−10	4.79E−09	GPR160
ENSG00000129595	–0.756168514	1.32E−09	6.44E−09	EPB41L4A
ENSG00000180739	–1.212317922	1.43E−09	0.000000007	S1PR5
ENSG00000104369	–0.713893389	1.49E−09	7.28E−09	JPH1
ENSG00000163884	–1.001721809	2.22E−09	1.06E−08	KLF15
ENSG00000172543	–1.443971336	2.6E−09	1.23E−08	CTSW
ENSG00000034533	–0.602893943	2.98E−09	1.41E−08	ASTE1
ENSG00000016391	–1.454323229	0.000000003	1.42E−08	CHDH
ENSG00000163412	–1.455095924	3.22E−09	1.51E−08	EIF4E3
ENSG00000130821	–0.69419495	3.28E−09	1.54E−08	SLC6A8
ENSG00000135929	–0.792267569	3.68E−09	1.72E−08	CYP27A1
ENSG00000174370	–1.005785086	4.1E−09	1.91E−08	C11orf45
ENSG00000025423	–0.998326574	4.63E−09	2.14E−08	HSD17B6
ENSG00000111199	–1.268393502	5.71E−09	2.62E−08	TRPV4
ENSG00000155974	–0.67183537	6.04E−09	2.76E−08	GRIP1
ENSG00000147606	–1.027590206	6.16E−09	2.81E−08	SLC26A7
ENSG00000065675	–1.501715725	6.81E−09	3.09E−08	PRKCQ
ENSG00000179841	–0.749380697	7.31E−09	3.31E−08	AKAP5
ENSG00000260822	–0.582080039	0.000000227	0.000000864	AC004656.1
ENSG00000156265	–0.79220517	8.14E−09	3.67E−08	MAP3K7CL
ENSG00000079215	–0.679149885	9.51E−09	4.25E−08	SLC1A3
ENSG00000221944	–0.600202219	9.68E−09	4.32E−08	TIGD1
ENSG00000101680	–0.905321065	1.11E−08	4.94E−08	LAMA1
ENSG00000126785	–0.618262682	1.15E−08	5.08E−08	RHOJ
ENSG00000205628	–0.582080325	0.000251743	0.000637414	LINC01446
ENSG00000223773	–0.583523523	0.008232673	0.015658057	CD99P1
ENSG00000282826	–0.583636435	0.008093028	0.015414788	FRG1CP
ENSG00000241544	–0.586635592	0.001586262	0.0034698	LINC02029
ENSG00000122420	–0.7597314	1.73E−08	7.48E−08	PTGFR
ENSG00000111879	–0.681429858	1.79E−08	7.72E−08	FAM184A
ENSG00000186162	–0.589358402	4.94E−16	4.13E−15	CIDECP
ENSG00000225793	–0.598603858	0.002182824	0.004653786	AL080250.1
ENSG00000164674	–0.834186124	2.35E−08	0.0000001	SYTL3
ENSG00000163710	–0.74510529	2.55E−08	0.000000108	PCOLCE2
ENSG00000138741	–1.124387768	2.62E−08	0.000000111	TRPC3
ENSG00000147003	–0.945694847	2.92E−08	0.000000123	TMEM27
ENSG00000168874	–1.171454266	3.51E−08	0.000000147	ATOH8
ENSG00000100077	–0.645354414	4.29E−08	0.000000178	GRK3
ENSG00000206652	–0.600319045	0.0041797	0.008468391	RNU1-1
ENSG00000056998	–1.159597278	4.96E−08	0.000000204	GYG2
ENSG00000110811	–1.364088188	5.18E−08	0.000000212	P3H3
ENSG00000234155	–0.603589219	0.0000182	0.0000545	AL135903.2
ENSG00000132718	–0.675745243	5.96E−08	0.000000243	SYT11
ENSG00000262202	–0.611008242	0.00012338	0.000327359	AC007952.4
ENSG00000263535	–0.612042556	0.004466662	0.009001187	AC134669.1
ENSG00000260966	–0.619021953	0.000673059	0.001578768	AP001486.2
ENSG00000100321	–1.052508227	0.000000103	0.000000411	SYNGR1
ENSG00000201558	–0.622105346	6.01E−11	3.37E−10	RNVU1-6
ENSG00000139173	–1.036479375	0.000000114	0.00000045	TMEM117
ENSG00000273320	–0.627836339	0.001733515	0.003763066	AC007032.1
ENSG00000175764	–0.612825531	0.000000149	0.000000581	TTLL11
ENSG00000196405	–0.658711721	0.000000158	0.000000615	EVL
ENSG00000169085	–0.59715125	0.000000164	0.000000633	C8orf46
ENSG00000255389	–0.628197788	0.004805773	0.009610814	Z97989.1
ENSG00000272821	–0.628905849	0.00000876	0.0000274	U62317.3
ENSG00000222365	–0.632247461	0.00000032	0.0000012	SNORD12B
ENSG00000245146	–0.63578959	0.002320634	0.004924648	LINC01024
ENSG00000184271	–0.985548652	0.000000242	0.000000921	POU6F1
ENSG00000027075	–0.603546465	0.000000268	0.00000101	PRKCH
ENSG00000249456	–0.640283418	0.002467147	0.00521089	AL731577.2
ENSG00000159263	–0.785381395	0.000000284	0.00000107	SIM2
ENSG00000268001	–0.641308425	0.000000459	0.00000168	CARD8-AS1
ENSG00000272779	–0.642724602	3.69E−15	2.9E−14	AC245060.4
ENSG00000261175	–0.644864636	0.001089512	0.00245603	LINC02188
ENSG00000259959	–0.649511362	0.00000882	0.0000276	AC107068.1
ENSG00000204228	–0.962404554	0.000000335	0.00000125	HSD17B8
ENSG00000114270	–0.589453951	0.00000035	0.0000013	COL7A1
ENSG00000226887	–1.126238941	0.000000395	0.00000146	ERVMER34-1
ENSG00000117425	–0.660152077	0.000000436	0.0000016	PTCH2
ENSG00000250299	–0.650539806	0.000742332	0.001725842	MRPS31P4
ENSG00000055732	–0.706315288	0.000000475	0.00000174	MCOLN3
ENSG00000233382	–0.654372192	0.001896591	0.004089349	NKAPP1
ENSG00000175745	–0.605230114	0.000000521	0.0000019	NR2F1
ENSG00000232093	–0.658713398	0.004308088	0.008706828	DCST1-AS1
ENSG00000165716	–0.84644461	0.000000701	0.00000252	FAM69B
ENSG00000131187	–0.649231899	0.00000073	0.00000261	F12
ENSG00000170775	–0.603129994	0.000000804	0.00000286	GPR37
ENSG00000164749	–0.714196763	0.000000822	0.00000292	HNF4G
ENSG00000256663	–0.661542619	0.00000329	0.0000109	AC112777.1
ENSG00000228742	–0.662633295	0.000530017	0.001268157	AC002384.1
ENSG00000198515	–1.13484411	0.00000108	0.00000381	CNGA1
ENSG00000127324	–0.591605084	0.00000113	0.00000397	TSPAN8
ENSG00000237870	–0.666779213	5.37E−08	0.00000022	AC073130.1
ENSG00000145040	–0.999709672	0.00000138	0.0000048	UCN2
ENSG00000197959	–0.728336114	0.00000158	0.00000543	DNM3
ENSG00000237187	–0.673528302	1.09E−14	8.29E−14	NR2F1-AS1
ENSG00000158050	–0.888282466	0.00000182	0.0000062	DUSP2
ENSG00000272269	–0.674471909	0.000000168	0.000000648	AL138724.1
ENSG00000162733	–0.85420253	0.00000199	0.00000675	DDR2
ENSG00000170458	–0.753322548	0.00000203	0.00000689	CD14
ENSG00000275131	–0.675077093	8.09E−09	3.65E−08	AC241952.1
ENSG00000246228	–0.676059031	3.1E−20	3.4E−19	CASC8
ENSG00000254887	–0.676505955	0.001307319	0.002905128	AC010247.1
ENSG00000230445	–0.683831123	0.0000288	0.0000842	LRRC37A6P
ENSG00000204335	–0.655622518	0.00000381	0.0000125	SP5
ENSG00000121207	–1.100166986	0.00000424	0.0000138	LRAT
ENSG00000151687	–0.657707604	0.00000479	0.0000156	ANKAR
ENSG00000153162	–0.713261029	0.00000516	0.0000166	BMP6
ENSG00000120693	–0.923295877	0.0000056	0.000018	SMAD9
ENSG00000169330	–0.583313432	0.00000629	0.0000201	KIAA1024
ENSG00000250731	–0.68687386	0.000258139	0.000651726	TPM3P6
ENSG00000130675	–0.922679936	0.00000653	0.0000208	MNX1
ENSG00000117318	–0.846439456	0.00000656	0.0000209	ID3
ENSG00000206052	–0.91285047	0.00000696	0.0000221	DOK6
ENSG00000119699	–0.79719179	0.00000704	0.0000223	TGFB3
ENSG00000100867	–0.695196402	0.00000731	0.0000231	DHRS2
ENSG00000244486	–0.991783568	0.00000738	0.0000233	SCARF2
ENSG00000166963	–1.102639672	0.00000789	0.0000249	MAP1A
ENSG00000254615	–0.690954391	9.46E−10	4.7E−09	AC027031.2
ENSG00000163009	–0.691160202	0.000483706	0.0011635	C2orf48
ENSG00000109705	–0.700668566	0.00000911	0.0000284	NKX3-2
ENSG00000178662	–0.707748281	0.00000999	0.0000311	CSRNP3
ENSG00000248121	–0.691464153	0.001506607	0.003312113	SMURF2P1
ENSG00000254893	–0.826566134	0.0000103	0.0000321	AC113404.3
ENSG00000164841	–0.622993759	0.0000104	0.0000323	TMEM74
ENSG00000215210	–0.702924697	0.001207828	0.002698653	RBMXP2
ENSG00000267383	–0.703518554	0.0000101	0.0000314	AC011447.3
ENSG00000277072	–0.70804624	0.000396301	0.000965996	STAG3L2
ENSG00000111012	–1.073265175	0.0000114	0.000035	CYP27B1
ENSG00000147036	–0.759385987	0.0000114	0.000035	LANCL3
ENSG00000186019	–0.7083644	0.000754381	0.001750497	AC021092.1
ENSG00000259673	–0.709890208	6.48E−10	3.27E−09	IQCH-AS1
ENSG00000258429	–0.624426456	0.0000121	0.0000371	PDF
ENSG00000141391	–0.60827287	0.0000122	0.0000373	PRELID3A
ENSG00000211448	–0.972541619	0.0000124	0.0000381	DIO2
ENSG00000115616	–0.665234335	0.0000133	0.0000408	SLC9A2
ENSG00000112599	–0.735375549	0.0000157	0.0000474	GUCA1B
ENSG00000204792	–0.712824082	0.00000285	0.00000946	LINC01291
ENSG00000170629	–0.718917939	7.44E−08	0.0000003	DPY19L2P2
ENSG00000270607	–0.721426273	0.0000854	0.000231989	AC009549.1
ENSG00000166415	–0.915490775	0.0000193	0.0000577	WDR72
ENSG00000188985	–0.721464876	0.000000975	0.00000344	DHFRP1
ENSG00000270696	–0.72171295	5.63E−13	3.76E−12	AC005034.3
ENSG00000261087	–0.722660516	0.000910157	0.00208174	AP003469.4
ENSG00000224167	–0.72658689	1.62E−08	7.06E−08	AL390729.1
ENSG00000115738	–0.875366803	0.0000248	0.0000733	ID2
ENSG00000007968	–0.997190263	0.0000264	0.0000779	E2F2
ENSG00000133687	–0.885164289	0.0000268	0.000079	TMTC1
ENSG00000260442	–0.727912721	0.000664516	0.001559564	ATP2A1-AS1
ENSG00000178184	–0.791109251	0.0000279	0.0000819	PARD6G
ENSG00000276672	–0.733771157	3.09E−18	3.03E−17	AL161891.1
ENSG00000177465	–0.817978872	0.0000291	0.0000851	ACOT4
ENSG00000264350	–0.736318413	0.0000175	0.0000525	AC090897.1
ENSG00000238142	–0.738440741	0.00083095	0.001913575	BX284668.5
ENSG00000143028	–0.730875189	0.0000305	0.0000888	SYPL2
ENSG00000173320	–0.595801591	0.000032	0.0000929	STOX2
ENSG00000260000	–0.738881279	0.0000167	0.0000505	AL133338.1
ENSG00000177822	–0.741978242	0.00003	0.0000875	AC098864.1
ENSG00000156140	–0.669690954	0.0000439	0.000124705	ADAMTS3
ENSG00000241360	–0.647292344	0.0000467	0.000132129	PDXP
ENSG00000149646	–0.661096929	0.0000498	0.000140129	CNBD2
ENSG00000164920	–0.58797654	0.0000546	0.000152834	OSR2
ENSG00000273033	–0.764755287	1.97E−08	8.47E−08	LINC02035
ENSG00000116819	–0.594037737	0.0000587	0.000163798	TFAP2E
ENSG00000274213	–0.767601803	0.000644991	0.001516995	AC015912.3
ENSG00000240929	–0.770831237	1.19E−19	1.28E−18	HIST2H2BB
ENSG00000184005	–0.872567649	0.0000797	0.000218143	ST6GALNAC3
ENSG00000247556	–0.7791386	8.2E−70	6.74E−68	OIP5-AS1
ENSG00000016402	–0.767097162	0.0000806	0.000220457	IL20RA
ENSG00000140465	–0.704109542	0.000082	0.000223701	CYP1A1
ENSG00000240024	–0.786762956	3.5E−29	6.14E−28	LINC00888
ENSG00000128596	–0.690766509	0.0000891	0.000241372	CCDC136
ENSG00000176125	–0.855443147	0.0000949	0.00025624	UFSP1
ENSG00000165730	–0.595042604	0.0000969	0.000261295	STOX1
ENSG00000119915	–0.779823964	0.000105488	0.00028257	ELOVL3
ENSG00000251350	–0.79220264	0.0000027	0.00000897	LINC02475
ENSG00000177181	–0.894622588	0.000124158	0.00032929	RIMKLA
ENSG00000280079	–0.80571522	5.25E−09	2.41E−08	AC011447.7
ENSG00000279059	–0.827433939	0.0000225	0.000067	AC007485.2
ENSG00000184486	–0.606547058	0.000159816	0.000418622	POU3F2
ENSG00000138395	–0.702091541	0.00016143	0.000422511	CDK15
ENSG00000095637	–0.610468574	0.000162344	0.000424481	SORBS1
ENSG00000275185	–0.828054896	0.00000027	0.00000102	AC130324.3
ENSG00000135643	–0.61786301	0.000180308	0.000468496	KCNMB4
ENSG00000205002	–0.777190629	0.000186764	0.000483708	AARD
ENSG00000229368	–0.82873561	0.0000576	0.000160817	AC090587.2
ENSG00000123096	–0.61800128	0.00020928	0.000537779	SSPN
ENSG00000160219	–0.713815817	0.000226571	0.000579039	GAB3
ENSG00000119737	–0.654203266	0.00024644	0.000625436	GPR75
ENSG00000263272	–0.832845527	0.0000107	0.0000332	AC004148.2
ENSG00000204789	–0.836003991	1.67E−10	8.96E−10	ZNF204P
ENSG00000279207	–0.839738829	0.00000227	0.00000765	AC015813.6
ENSG00000278291	–0.844858432	0.000507367	0.001217292	AL161772.1
ENSG00000233117	–0.857807409	2.24E−13	1.54E−12	LINC00702
ENSG00000164778	–0.858184757	0.000294259	0.000735145	EN2
ENSG00000173376	–0.799990294	0.000322836	0.000799238	NDNF
ENSG00000186340	–0.612224671	0.000327986	0.000810917	THBS2
ENSG00000226608	–0.86563425	2.31E−08	9.86E−08	FTLP3
ENSG00000150510	–0.635813794	0.000354515	0.000870281	FAM124A
ENSG00000129167	–0.767846935	0.000362165	0.000887408	TPH1
ENSG00000255224	–0.871481211	0.000139626	0.000368601	AC109322.1
ENSG00000132932	–0.844803117	0.000408508	0.000992987	ATP8A2
ENSG00000179546	–0.62615245	0.000418009	0.001014768	HTR1D
ENSG00000124772	–0.758950016	0.000448513	0.001084408	CPNE5
ENSG00000132016	–0.595302993	0.000460125	0.001110729	C19orf57
ENSG00000227908	–0.87629061	0.0000218	0.0000647	FLJ31104
ENSG00000272462	–0.889371251	0.000000306	0.00000115	U91328.2
ENSG00000232368	–0.891946046	8.72E−10	4.34E−09	FTLP2
ENSG00000274020	–0.902527943	0.000000219	0.000000837	LINC01138
ENSG00000167705	–0.656826415	0.00055993	0.001333171	RILP
ENSG00000163596	–0.716182102	0.000585479	0.001386711	ICA1L
ENSG00000162601	–0.652501753	0.000599957	0.0014187	MYSM1
ENSG00000250132	–0.906972213	0.000126211	0.000334668	AC004803.1
ENSG00000143494	–0.629613078	0.000659815	0.001549361	VASH2
ENSG00000276107	–0.914373714	3.98E−09	1.85E−08	AC037198.2
ENSG00000238266	–0.920917248	2.39E−11	1.39E−10	LINC00707
ENSG00000128203	–0.656798742	0.000682964	0.001599429	ASPHD2
ENSG00000187391	–0.775390286	0.000695998	0.001627632	MAGI2
ENSG00000198157	–0.672184208	0.000719226	0.001676278	HMGN5
ENSG00000266709	–0.92347604	1.49E−15	1.2E−14	AC005224.4
ENSG00000269927	–0.941179574	0.0000116	0.0000356	AC004817.3
ENSG00000224080	–0.957073973	0.00000138	0.00000479	UBE2FP1
ENSG00000171119	–0.732024185	0.000859632	0.001973741	NRTN
ENSG00000182795	–0.713257	0.000893473	0.002045361	C1orf116
ENSG00000235859	–0.981085776	1.34E−11	7.99E−11	AC006978.1
ENSG00000184058	–0.781722778	0.000947799	0.002160685	TBX1
ENSG00000137959	–0.589383242	0.000982869	0.00223288	IFI44L
ENSG00000249485	–0.994236206	0.0000105	0.0000326	RBBP4P1
ENSG00000188848	–0.612794187	0.00106899	0.00241599	BEND4
ENSG00000235180	–1.024177912	1.2E−09	5.92E−09	LINC00601
ENSG00000280123	–1.03782143	2.74E−12	1.73E−11	AC023632.6
ENSG00000176912	–1.038065676	3.47E−10	1.79E−09	TYMSOS
ENSG00000134317	–0.746307481	0.001354336	0.003003002	GRHL1
ENSG00000130208	–0.676215853	0.001374168	0.003039733	APOC1
ENSG00000250462	–1.061344837	4.22E−32	8.64E−31	LRRC37BP1
ENSG00000155465	–0.67282726	0.001515904	0.00333088	SLC7A7
ENSG00000230316	–1.096386598	3.09E−10	1.61E−09	FEZF1-AS1
ENSG00000115318	–0.601269693	0.001614401	0.003523135	LOXL3
ENSG00000125931	–0.665048797	0.001714986	0.003725307	CITED1
ENSG00000261534	–1.102227861	7.9E−10	3.95E−09	AL596244.1
ENSG00000214719	–1.106140664	1.01E−16	8.81E−16	AC005562.1
ENSG00000234456	–1.116449494	4.03E−22	4.92E−21	MAGI2-AS3
ENSG00000092200	–0.685034084	0.001895898	0.004088527	RPGRIP1
ENSG00000216775	–1.169568862	1.42E−39	4.14E−38	AL109918.1
ENSG00000260686	–1.172038199	0.000000482	0.00000176	AC008669.1
ENSG00000226943	–1.177930323	1.53E−08	6.66E−08	ALG1L5P
ENSG00000188185	–1.185262956	0.000001	0.00000354	LINC00265
ENSG00000113389	–0.597567416	0.002456821	0.005192422	NPR3
ENSG00000248890	–1.205696789	6.08E−18	5.82E−17	HHIP-AS1
ENSG00000170270	–0.68387924	0.002558896	0.005392531	GON7
ENSG00000119508	–0.711455187	0.002680663	0.005624765	NR4A3
ENSG00000140905	–0.636524948	0.002992587	0.006224563	GCSH
ENSG00000050730	–0.636431758	0.003037792	0.006309593	TNIP3
ENSG00000170989	–0.64441633	0.003150496	0.006522018	S1PR1
ENSG00000256304	–1.233661403	2.46E−13	1.68E−12	CCDC150P1
ENSG00000237424	–1.291854855	1.19E−11	7.12E−11	FOXD2-AS1
ENSG00000227359	–1.292403358	0.000000113	0.000000447	AC017074.1
ENSG00000230615	–1.294100524	2.92E−32	6.03E−31	AL139220.2
ENSG00000244300	–1.311706541	6.67E−08	0.000000271	GATA2-AS1
ENSG00000183762	–0.599097723	0.004686398	0.009396411	KREMEN1
ENSG00000168405	–1.328267416	4.82E−09	2.23E−08	CMAHP
ENSG00000144834	–0.620458505	0.004968065	0.00989472	TAGLN3
ENSG00000279519	–1.3382021	3.67E−12	2.3E−11	AC007382.1
ENSG00000105371	–0.591348772	0.00761156	0.014575965	ICAM4
ENSG00000128536	–0.58264386	0.007717241	0.014763271	CDHR3
ENSG00000226806	–1.402676685	0.000000047	0.000000194	AC011893.1
ENSG00000257732	–1.507011758	2.49E−14	1.83E−13	AC089983.1

To test whether the effects on chromatin in *LOXL2* KD cells directly activated ATM-dependent DDR signaling, we forced chromatin condensation in these cells by expressing the linker histone H1 or the H3K9 methyltransferase SUV-39H1 (as green fluorescent protein (GFP)-labeled proteins) and counted the number of γ-H2AX foci in GFP-positive cells. Notably, overexpression of either H1^GFP^ or SUV-39^GFP^ in *LOXL2* KD cells reduced the number of γ-H2AX foci as compared with *LOXL2* KD cells that expressed GFP alone (+MOCK^GFP^) (Fig. 4f, g). This suggested that lack of LOXL2 and reduced H3K4ox levels affected the regulation of chromatin condensation (leading to decondensation) and activated DDR, even in the absence of DNA damage.

### LOXL2 reduction enhances chemosensitivity of TNBC cells

**Fig. 5:**
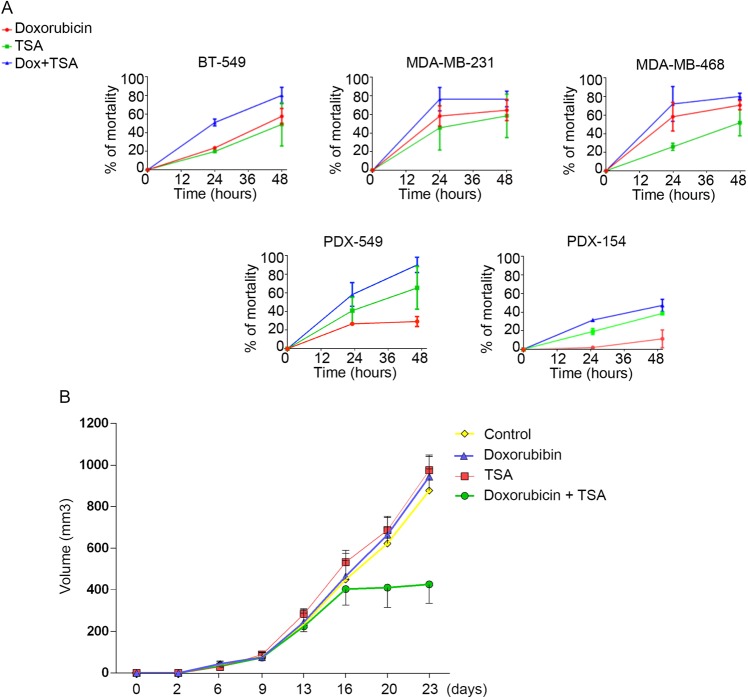
LOXL2 suppresses sensitivity in TNBC cells. **a** Cell viability was measured at different timepoints using an MTT assay of cultured different breast cancer cells and PDXs. The effects of doxorubicin, TSA, and doxorubicin plus TSA were analyzed. **b** Dissociated cells from PDX-549 were orthotopically implanted into NOD/SCID mice and injected intraperitoneally twice weekly with TSA (0.25 mg/kg), doxorubicin (2 mg/kg mouse weight), or doxorubicin plus TSA. Tumor volumes were measured twice a week and are given as averages. Results are given as averages of six independent tumors ± SEM

We found that reducing H3K4ox levels via LOXL2 depletion led to chromatin decondensation, triggered DDR activation, and induced cell cycle arrest, suggesting that LOXL2 inhibition could be interesting as a breast cancer treatment. As no LOXL2-specific inhibitors are currently available, we therefore tested whether reducing the levels of functional LOXL2 would increase the sensitivity of breast cancer cells to chemotherapy. For this, we treated three different TNBC cell lines with doxorubicin (a topoisomerase inhibitor that generates DSBs) either alone or together with trichostatin A (TSA) (a general HDCA inhibitor [37] that increases chromatin accessibility [38]). Indeed, we observed that in all cases, treatment of doxorubicin together with TSA increased the percentage of cell death—indicative of an increased cell sensitivity to chemotherapy (Fig. 5a). Similar results were obtained with two TNBC_PDXs (PDX-549 and PDX-154) (Fig. 5a). To determine if these results were reproducible in vivo, PDX-549 cells were first grown ex vivo, and then 10^6^ cells were subcutaneously implanted in nude mice. After tumor formation, mice were treated with TSA, doxorubicin, doxorubicin plus TSA, or (as a control) vehicle for 25 days. In agreement with our previous results, we observed that tumor growth was substantially impaired with the combined TSA/doxorubicin treatment (Fig. 5b).

## Discussion

In this work, we found that several TNBC cell lines and PDXs express high levels of LOXL2 as compared with those of the luminal breast cancer subtype. With the use of a newly generated antibody, we were able to detect the H3K4ox modification produced by LOXL2 [3] and we observed how this histone H3 modification was higher in all TNBC cell lines and PDXs as compared with luminal subtypes. This H3 modification is enriched in heterochromatin and is required to maintain the condensed heterochromatin state. It is possible that during tumor progression, some cancer cells undergo the EMT program and start to express LOXL2. The transcription factor Snail1, together with LOXL2, would participate in downregulating the *CDH1* gene and other heterochromatin transcripts, giving rise to transformation of cancer epithelial cells into mesenchymal cells (through EMT) [6, 39–41]. Recently, two different groups have suggested that EMT is dispensable for lung and pancreas metastasis but contributes significantly to the formation of recurrent metastasis after chemotherapy [42, 43]. This finding is in agreement with our results, in which we observed that the most aggressive and resistant subtype of breast cancer (TNBC) has a mesenchymal phenotype and is enriched for H3K4ox in its heterochromatin. We observed that reducing H3K4ox levels perturbed the balance between condensed and decondensed chromatin and activated the DDR. Similar phenotypes have been previously observed after depletion of SUV-39, PRDM2, or HP1, which are required for heterochromatin maintenance and cell survival after DNA damage [23, 24, 44, 45]. H3K4ox levels have been correlated to the repression of *CDH1* and major satellites (heterochromatin transcripts) during the EMT program [3, 4, 6]. *CDH1* is upregulated in TNBC cells when *LOXL2* is knocked down and the levels of H3K4ox are decreased; however, the H3K4ox modification does not seem to be involved in repression of heterochromatin transcripts, as we did not observe increased levels of heterochromatin transcripts in *LOXL2* KD conditions. Notably, downregulation of heterochromatin transcripts during EMT occur at a specific timepoint during chromatin reorganization, after which the levels of transcription recovered despite the presence of H3K4ox. Intriguingly, although upstream DDR signaling is sustained when chromatin is maintained in a condensed state [22, 46, 47], we found that forcing chromatin decondensation was sufficient to activate DDR signaling in the absence of any detectable DNA lesions. It seems that an abnormal, unbalanced ratio between condensation and decondensation leads to persistent DDR signaling. As LOXL2 has multiple substrates besides histones [5], we cannot discard an additional putative role for LOXL2 in the direct regulation of the DDR machinery. However, we observed that the DDR induced in the absence of LOXL2 can be inhibited by forcing chromatin to condense using either of two different approaches, suggesting that the main molecular mechanism is the regulation per se of the chromatin compaction state.

While a correlation between chromatin compaction and DDR has been previously reported [22, 46, 47], we demonstrated here that H3 oxidation by LOXL2 is another molecular mechanism that maintains compaction. Notably, *LOXL2* has been widely linked to cancer and the acquisition of cellular malignancy, as it is overexpressed in many tumors [15, 48–51] and has an important role in tumor formation [52].

Induction of chromatin compaction has been suggested as a potential therapeutic tool in gliomas, in which chromatin compaction limits DDR [46] and promotes damage resistance [53]. However, this does not appear to apply to TNBC cells. In support of this, we have now shown that several TNBC PDX tumors treated with TSA, a histone deacetylase inhibitor that generates an open chromatin state, enhanced tumor killing with doxorubicin in these cells. These observations suggest a rationale for studying combinations of “chromatin opening drugs,” including inhibitors of histones deacetylases, EZH2, or LSD1, as a strategy for overcoming resistance to treatment in TNBC.

## Materials and methods

### Cell lines, transfections, and infections

Most of the cell lines used in this work (the TNBC lines MDA-MB-468 (ATCC HTB-132), CAL-51 (CSC-C0382), HS-578T (ATCC HTB-126), and MDA-MB-231 (ATCC HTB-26), the luminal A T-47D (ATCC HTB-133), MCF-7 (ATCC HTB-22D), and luminal B BT-474 (ATCC HTB-20)) as well as cells isolated from PDXs, were maintained in Dulbecco’s modified Eagle’s medium (Biowest; L0106-500); the TNBC line BT-549 (ATCC HTB 122) was maintained in Roswell Park Memorial Institute medium (Biowest; L0501-500). All cell cultures were supplemented with 10% fetal bovine serum (Gibco; 10270106), 2 mM l-glutamine (Biowest; X0550-100), and 1% penicillin-streptomycin (Gibco; 15140122) at 37 °C in 5% CO_2_. All cell lines were regularly tested for the absence of mycoplasma using standard polymerase chain reaction (PCR). For LOXL2-Flag overexpression assays, MCF-7 cells were seeded for 24 h and transfected with 10 µg pcDNA3-hLOXL2-Flag vector using polyethylenimine polymer (Polysciences Inc; 23966-1). Lentiviral particles were produced in HEK293T cells (ATCC CRL-3216) for lentiviral infections to produce *LOXL2* KD cells. Cells were grown to 70% confluency and then transfected by dropwise addition of NaCl (150 mM), a DNA mixture of pLKO-short hairpin control (shCT)/shLOXL2 (7.5 µg), pCMV-VSVG (1.5 µg), pMDLg/pRRE (4.5 µg), and pRSV-Rev (1.5 µg), and polyethylenimine polymer (1 mg/ml) (Polysciences Inc), which had been preincubated for 15 min at room temperature. Transfection medium was replaced with fresh medium after 24 h (day 1). The supernatant from the transfection medium at days 2 and 3 was filtered with 0.45 μm filter unit (Merck Millipore) and stored at 4 °C (both were mixed after day 2). The supernatant mixture was concentrated using Lenti-X Concentrator product (Clontech) and centrifuged at 1500 × *g* for 45 min at 4 °C. The pellet was resuspended in 1 ml fresh medium, aliquoted (100 μl), and stored at –20 °C. Aliquots were used to infect MDA-MB-231 cells [6]. For retroviral infections, HEK293 gag–pol cells were used to produce retroviral particles. Cells were transfected as for HEK293T cells with a mixture of NaCl (150 mM), DNA (2.5 µg of pCMV-VSV-G and 7.5 µg of pMSCV, pMSCV-LOXL2 wt-FLAG or pMSCV-LOXL2 mutFLAG IRES–GFP vectors) and polyethylenimine polymer (1 mg/ml) (Polysciences Inc; 23966-1) that had been preincubated for 15 min at room temperature; transfection medium was replaced with fresh medium at 24 and 48 h and processed as explained above. Viral particles were concentrated using Retro-X Concentrator product (Clontech; 631456) and then used to infect MDA-MB-231 cells. HEK293T cells were used to produce lentiviral particles for histone H1(H1)-GFP expression. Cells were first grown to 70% confluency and transfected with a mixture of NaCl (150 mM), a DNA mixture of FUGW-H1-empty vector/FUGW-H1-GFP (7.5 µg), pCMV-VSVG (1.5 µg), pMDLg/pRRE (4.5 µg), and pRSV-Rev (1.5 µg), and polyethylenimine polymer (1 mg/ml) (Polysciences Inc; 23966-1), which had been preincubated for 15 min at room temperature and processed as explained above. Viral particles were concentrated using Lenti-X Concentrator product (Clontech; 631232) and used to infect MDA-MB-231 cells. For SUV-39H1-GFP rescue experiments, MDA-MB-231 cells infected with either shCT or shLOXL2 were selected for 48 h with puromycin (Puro) (2.5 μg/ml). After selection, cells were seeded for 24 h and transfected with CGA-pCAGGS-SUV39H1-EGFP-IRES-Puro vector (2.5 µg) using TransIT-X2 Dynamic Delivery System (Mirus Bio LLC; MIR600Q).

### Antibodies and other reagents

The following antibodies were used: anti-FLAG (F7425, Sigma), anti-LOXL2 (NP1-32954, Novus), anti-H3K4me3 (07-473, EMD Millipore), anti-H3K9me3 (07–442, Millipore), anti-phospho-histone H2AX (S139) clone JBW301 (05–636, EMD Millipore), anti-GFP (ab6556, Abcam), anti-53BP1 (NB100–904, Novus Biol.), anti-phospho-CHK1 (S317) (A300-163A, Bethyl), anti-CHK2 clone 7 (05–649, EMD Millipore), anti-cleaved caspase 3 (Asp175) (9661, Cell Signaling), anti-KAP-1 (ab10484, Abcam), anti-Phospho-KAP-1 (S824) (A300-767A, Bethyl), anti-tubulin (T9026, Sigma), anti-phospho-H3 (S10) (06–570, Millipore), and anti-histone H3 (ab1791, Abcam). The following chemical reagents were used: doxorubicin hydrochloride (Sigma; D1515), cordycepin (Sigma; C3394), and the ATM inhibitor KU55933 (Sigma; SML1109). An anti-H3K4ox antibody was generated from a synthetic peptide in which fluorenylmethoxycarbonyl protecting group (Fmoc)-6-hydroxynorleucine (BAA1117, Iris Biotech) rather than Fmoc-lysine(Boc) was incorporated at position 4. The peptide was purified (>95%) by reversed-phase high-performance liquid chromatography and its identity was confirmed by mass spectrometry, after which it was coupled to keyhole limpet hemocyanin (KLH) for antibody production. Antibody information can be found in the Supplementary Table 1.

### Cell extracts, histone isolation, and PDX extraction

To obtain nuclear fractions of LOXL2-Flag–transfected MCF-7 cells and HEK293T cells, cells were first lysed in soft-lysis buffer (50 mM Tris, 2 mM EDTA, 0.1% NP-40, and 10% glycerol, supplemented with protease and phosphatase inhibitors) for 5 min on ice. Samples were centrifuged at 900 × *g* for 15 min, and the supernatant was discarded. The nuclear pellet was lysed in high-salt lysis buffer (20 mM HEPES pH 7.4, 350 mM NaCl, 1 mM MgCl_2_, 0.5% Triton X-100, and 10% glycerol, supplemented with protease and phosphatase inhibitors) for 30 min at 4 °C, and samples were centrifuged at 16,000 × *g* for 10 min supernatant NaCl concentration was reduced to 300 mM NaCl by adding balance buffer (20 mM HEPES pH 7.4, 1 mM MgCl_2_, and 10 mM KCl). Cells extracts from the different breast cancer cell lines were obtained with SDS lysis buffer (2% SDS, 50 mM Tris-HCl, and 10% glycerol). Histones were isolated from the different breast cancer cell lines using acid precipitation. For this, cell monolayers were first scraped in 1 ml of lysis buffer (10 mM Tris pH 6.5, 50 mM sodium bisulfite, 10 mM MgCl_2_, 1% Triton X-100, 8.6% sucrose, and 10 mM sodium butyrate). Working at 4 °C, pellets were then purified by three rounds of centrifugation (16,000 × *g* for 15 s) and resuspension by vortexing, in lysis buffer for the first two rounds and washing buffer (10 mM Tris pH 7.4, 13 mM EDTA) for the final round; supernatant fractions were discarded. The obtained pellets containing chromatin were resuspended in 0.4 N sulfuric acid, the mixture was incubated at 4 °C for 1 h and then centrifuged at 16,000 × *g* for 10 min, and the histone-containing supernatants were kept. Samples were incubated with acetone (1:9) to block the acid overnight at –20 °C and then centrifuged at 16,000 × *g* for 10 min histone pellets were air dried for 5 min and then resuspended in water for analysis. Cell extracts of PDX samples were obtained by disrupting tissue using a pellet pestle (Sigma; Z359947) with lysis buffer (50 mM Tris-HCl pH 8, 10 mM EDTA, 1% SDS, and 1 mM DTT). Proteins were separated by SDS-polyacrylamide gel electrophoresis gel and analyzed with the indicated antibodies.

### Recombinant LOXL2 purification

LOXL2-encoding baculovirus were amplified, and LOXL2-Flag recombinant proteins (wt and mutant) were produced in Sf9 cells according to standard procedures [3]. Cell lysis was performed as previously described [54]. Cell extracts were incubated with Flag M2 beads for 4 h at 4 °C and then washed 4× times with 20 mM HEPES pH 7.4, 1 mM MgCl_2_, 300 mM NaCl, 10 mM KCl, 10% glycerol, and 0.2% Triton X-100. Recombinant proteins were eluted with the Flag peptide (1 µg/µl) for 1 h at 4 °C.

### Oxidation reaction

Nucleosomes (2 µg) were incubated with recombinant LOXL2 purified protein in oxidation buffer, 20 mM HEPES pH 7.4, 100 mM NaCl, 1 mM MgCl_2_, and 1 mM DTT. Reactions were carried out for the indicated times at 37 °C and then analyzed by SDS/PAGE and western blotting. To detect the aldehyde group, biotin hydrazide (BTH) (5 mM) was added after the oxidation reaction for 2 h at 25 °C. Finally, biotinylated histones were immunoprecipitated with streptavidin beads, and oxidized H3 was detected by western blot.

### Dot blot assay

For dot blot assays, 1 µg of each peptide (in 10 µl of sample) was applied under low vacuum to a prewetted nitrocellulose membrane (Amersham Protran 0.45 nitrocellulose, GE Healthcare) using a dot blot apparatus (HYBRI-DOT Manifold; Life Technologies). After blocking the entire blot in 15 mL of 5% nonfat dry milk and 0.1% Tween-20 Tris-buffered saline for 1 h at room temperature, the blot was probed with the indicated antibodies.

### ChIP experiments

For ChIP experiments, cells were first crosslinked in 1% formaldehyde for 10 min at 37 °C. Crosslinking was stopped by adding glycine to a final concentration of 0.125 M for 2 min at room temperature. Cell monolayers were scraped in cold soft-lysis buffer (50 mM Tris pH 8.0, 10 mM EDTA, 0.1% NP-40, and 10% glycerol), and incubated 20 min on ice. Nuclei pellets were lysed with SDS lysis buffer (1% SDS, 10 mM EDTA, 50 mM Tris pH 8.0), and extracts were sonicated to generate 200–1500-bp DNA fragments. For immunoprecipitation, supernatants were diluted 1:10 with dilution buffer, and samples were incubated with rotation overnight at 4 °C with primary antibody or irrelevant immunoglobulin G. Samples were then treated with elution buffer (100 mM Na_2_CO_3_ and 1% SDS) for 1 h at 37 °C and incubated at 65 °C overnight after addition of NaCl to a final concentration of 200 mM, to reverse formaldehyde crosslinking. After proteinase K treatment for 1 h at 55 °C, DNA was purified with MinElute PCR purification kit (Qiagen; 28006) and eluted in Milli-Q water. Genomic regions (GRs) were detected by quantitative staining with PCR SYBR Green (Quantabio; 95073), and the ChIP results were quantified relative to the input amount and the amount of H3 immunoprecipitated in each condition.

Peaks of H3K4ox were called from sequence reads detected through ChIP-seq using the MACS2 tool [26]. The chromatin state files for HepG2 and HMEC cells were computed by the ENCODE project using the ChromHMM tool [27] from https://genome.ucsc.edu/cgi-bin/hgFileUi?db=hg19&g=wgEncodeBroadHmm. Statistical overrepresentation of H3K4ox peaks detected by ChIP-seq was assessed from the two cell lines across several chromatin states: heterochromatin, repressed, insulator, strong enhancer (sum of states 4 and 5), poised/weak enhancer (sum of states 6 and 7), promoter (sum of states 1 and 2), and poised promoter. The contingency table of the Fisher's test carried out for this contained the number of nucleotides within peaks, chromatin states, intersections thereof, and the remaining portion of the genome (computed as the difference from the effective genome size for ChIP-seq peaks calling). The same procedure was applied to detect H3K4ox peak overrepresentation in lamin-associated domains of chromatin, obtained from [55]. ChIP-PCR used the following GR number and peak positions: GR#1: chr 14 (79003315–79007307); GR#2: chr 5 (151967551–151971984); GR#3: chr 1 (230657019–230658589); GR#4: chr 2 (41741709–41745780); GR#5: chr 17 (53951879–53955135); GR#6: chr 4 (62611008–62615505); GR#7: chr 6 (67550237–67554488); GR#8: chr 5 (66894215–66899021); and GR#9: chr 13 (89334968–89336774). The sequences of the primers used can be found in Supplementary Table 2.

### ATAC-seq and ATAC-qPCR experiments

ATAC experiments were performed as described previously [28]. Cells were harvested and treated with transposase Tn5 (Nextera DNA Library Preparation Kit, Illumina; FC-121-1030). DNA was purified using MinElute PCR purification kit (Qiagen), samples were amplified by PCR using NEBNext High-Fidelity 2× PCR Master Mix (New England Biolabs; M5041), and DNA was again purified with the MinElute PCR purification kit. Reads produced by ATAC sequencing of two control (Control) replicates and two LOXL2 KD sequencing replicates (LOXL2 KD) were aligned to the hg19 build of the reference human genome using Bowtie 2 [56] with default parameters for pair-end sequencing. ATAC peaks were then called by combining aligned reads of both replicates of the control and the KD using MACS2. To allow for false discovery rate (FDR) threshold selection further downstream in the analysis, no FDR restrictions were imposed on the ATAC peak calling. For ATAC-qPCR experiments, the final elution product of the ATAC protocol was diluted 1:50. Incorporation of the transposase Tn5 to GRs was detected by quantitative staining with PCR SYBR Green (Quantabio; 95073). ATAC-qPCR results for the selected GRs were normalized to the same unaffected GR (*HPRT* promoter) for each condition. The sequences of the primers used can be found in Supplementary Table 2.

### RNA-seq analysis

Reads produced by RNA-seq of the same replicates as described above of two controls (Control) and two *LOXL2* KDs were aligned to the hg19 build of the reference human transcriptome using TopHat2 [57] with default parameters for pair-end sequencing. Aligned reads were then analyzed using a standard Cufflinks pipeline [58] to detect differentially expressed genes between the two conditions (*LOXL2* KD and Control).

### Differential expression analysis of transposable elements (TEs)

The trimmed RNA-seq reads from the two controls and the two LOXL2 samples were processed with the TEtools program [59] using the hg19 annotation of TEs. The obtained count table was then processed with NOIseq [60] to perform a differential expression analysis of LOXL2 against the control. Expression was normalized using the TMM method, whereby TEs with a probability higher than 0.95 of being differentially expressed were considered as statistically significant.

### Replicate correlation

The read count (coverage) at each position of the hg19 human reference genome was computed for each replicate of the H3K4ox ChIP-seq and the control and *LOXL2* KD ATAC sequencing, using the BEDtools genomecov capability [61]. Genomic positions with zero-read counts were filtered out. Replicate files of each experiment were merged to produce a single file aligned by genomic position, and the corresponding Pearson’s correlation coefficient of read counts was computed. For the graphical representation of the correlation, 100,000 genomic positions were randomly selected.

### Analysis of ATAC peaks that overlap with H3K4ox peaks

All significant (*P* < 10^–5^) ATAC peaks (*LOXL2* KD versus control) and H3K4ox peaks were first intersected with the BEDtools *intersect* program [61]. Based on this intersection, ATAC peaks were classified as *overlapping* (if they intersected an H3K4ox peak) or *orphan* (if not). Only intersections involving more than 95% of the sequence of ATAC peaks were considered. Control and *LOXL2* KD read counts over all genomic positions (see above) were intersected with both overlapping and orphan peaks. Read counts over genomic positions of control and experiment replicates were averaged. To carry out the heatmap representation, peak sequences (overlapping or orphan) were aligned by their summits. For linear representation, the average experimental read counts at each downstream and upstream position were summed for both the experimental and the control counts. Position-wise sums were then divided by the read count sum value obtained for the summit of control read counts, thus making all sums relative to the maximum control value.

### Integrated analysis of H3K4ox and ATAC peaks, and differentially expressed genes

The differentially expressed genes detected through the RNA-seq analysis of control and *LOXL2* KD cells were selected if they were in close vicinity (either up- or downstream) to overlapping ATAC peaks. Two different distance thresholds (0.5 and 1 Mb) were used to detect close differentially expressed genes.

### Inhibition of RNA synthesis, ATM kinase, and cell cycle analysis

MDA-MB-231-infected cells selected for 48 h with puromycin (2.5 μg/ml) were seeded and then maintained under puromycin selection.

For inhibition of RNA synthesis, cells were treated at 48 h with 200 µM cordycepin (Sigma; C3394) and fixed with 100% cold ethanol at designated timepoints.

For inhibition of the ATM kinase, cells were incubated at the 24-h timepoint with KU55933 (Sigma; SML1109) for a further 24 h at two different concentrations (either 5 or 10 µM), or for 2 h with doxorubicin (1 µM) as a positive control.

For cell cycle analysis, cells at 48 h after puromycin selection were first synchronized through the double thymidine block protocol. Specifically, cells were seeded to 50% confluency, incubated for 14 h with complete growth medium (supplemented with 2 mM thymidine), washed 2× with phosphate-buffered saline (PBS), and released by a 9-h incubation with complete medium growth. Cells were washed again 2× with PBS, incubated 14-h with 2-mM thymidine, and released with complete growth medium. Cells were then harvested at the designated timepoints and fixed with 100% cold ethanol. After two days, fixed cells were stained with propidium iodide (PI) and analyzed by flow cytometry using BD FACSCalibur (Becton Dickinson). Results were analyzed using BD CellQuest Pro software.

### Non-replicative cell experiment

MDA-MB-231 cells were seeded in coverslips and maintained during all the experiment in Dulbecco’s modified Eagle’s medium (Biowest; L0106-500) with 0.5% fetal bovine serum (Gibco; 10270106) at 37 °C in 5% CO_2_. After 24 h, cells were infected with lentiviral particles for *LOXL2* KD. After 96 h under selection, cells were fixed with paraformaldehyde 4% with PBS.

### Comet assay

MDA-MB-231-infected cells under puromycin selection (see above) were seeded at the 48-h timepoint and grown another 48 h more (still with puromycin at 2.5 µg/ml). DNA strand breaks were measured at the single-cell level for MDA-MB-231 control or *LOXL2* KD cells using an alkaline comet assay with the CometAssay kit (Trevigen, Gaithersburg, MD) following the manufacturer's instructions. Briefly, 5000 cells in 50 μl PBS were combined with 500 μl molten LMAgarose (at 37 °C), and 50 μl of this was immediately transferred to a CometSlide. After a 10-min incubation at 4 °C, slides were immersed in 4 °C lysis solution and incubated overnight at 4 °C. Slides were then immersed in freshly prepared alkaline solution (200 mM NaOH, 1 mM EDTA pH > 13) for 1 h at 4 °C in the dark. For electrophoresis, slides were placed in an electrophoresis slide tray containing alkaline solution and incubated with voltage (21 V) for 30 min. After that, slides were immersed twice in H_2_O for 5 min and then once in 70% ethanol for 5 min. Samples were dried at 37 °C and stained with SYBR Green staining solution (SYBR Green I, Invitrogen) using a 1:10000 dilution in TE (10 mM Tris-HCl pH 7.5, 1 mM EDTA) for 30 min Finally, slides were dried at 37 °C, and cells were imaged using an Olympus BX61 microscope.

### Rescue experiments

MDA-MB-231-infected cells under puromycin selection (see above) were seeded at the 48-h timepoint, incubated under puromycin selection for an addition 24 h, and then transfected with the SUV-39H1-EGFP vector or reinfected with retroviral particles for LOXL2-FLAG or lentiviral particles for H1 expression. After 24 h, cells were fixed for immunofluorescence.

### Immunofluorescence, image acquisition, and analysis

Cells were fixed with 4% paraformaldehid for 15 min at room temperature, blocked for 1 h with 1% PBS-bovine serum albumin, incubated at room temperature for 2 h with primary antibody, washed 3× with PBS, and then incubated for 1 h at room temperature with the secondary antibody. Cells were washed again 3× with PBS, incubated for 5 min with 4′,6-diamidino-2-phenylindole (DAPI) (0.25 mg/ml) for cell nuclei staining, and then mounted with fluoromount. Fluorescence images corresponding to DAPI, GFP, γ-H2AX and 53BP1 were acquired in a Leica TCS SPE microscope using a Leica DFC300 FX camera and the Leica IM50 software.

### Metaphase spreads

For metaphase spread preparations, cells were treated with colcemid (0.1 ug/ml) for 4 h. Cells were trypsinized, hypotonically swollen in 0.075 M KCl for 15 min at 37 °C, and then fixed (75% MeOH and 25% acetic acid, ice cold). Metaphase preparations were spread on glass slides, stained with 10% Giemsa stain (Sigma), and mounted in DPX mounting medium (PanReac). Images were taken using a Leica DM6000 microscope (Leica, Wetzlar, Germany) and analyzed using Fiji Software (https://fiji.sc/).

### Cellular viability experiment

Breast cancer cell lines and PDX cells were seeded in 96-well plates. Cellular viability was analyzed using Thiazolyl Blue Tetrazolium Bromide (MTT) (Sigma; M5655) at different timepoints. The concentrations used were: 0.1 µM doxorubicin for all samples; 250-nM TSA for the breast cancer cell lines; and 500-nM TSA for the PDXs cells. Absorbance was detected at 565 nm on Infinite® 200 PRO Series Multimode Reader (Tecan Group Ltd.) and analyzed with i-control™ Microplate Reader Software (Tecan Group Ltd.)

### Cancer PDXs and treatments

Samples from patients with breast cancer were obtained from the operating room and transferred to the pathology department, where breast cancer samples were collected, transferred to the animal facility, and implanted into mice, to generate PDXs. All samples were implanted within 60 to 90 min after surgical removal. All patients willingly signed an informed consent, and the study was approved by the Ethics Committee of the Vall d'Hebron Hospital.

For in vivo experiments, a tumor from an established PDX was dissociated into single cells by enzymatic digestion (collagenase at 300 U/ml and hyaluronidase at 200 U/ml) during 1 h at 37 °C on a rotating wheel. The solution of digested tumor was treated with 0.025% trypsin and filtered sequentially using 100 and 40 µm strainers. Isolated cells were plated in culture dishes with DMEM-F12 supplemented with FBS, glutamine, and penicillin/streptomycin. Once the culture was established, 10^6^ cells were injected into the number four fat pad of 6-week old NOD.CB17PrkdcSCID/J (NOD/SCID) female mice (Charles River) with Matrigel. For this, mice were anesthetized and shaved, the fourth and fifth sets of nipples were localized, and an inverted Y incision was made from the midline point between the fourth set of nipples, ending between the fourth and fifth sets to expose the fourth and fifth fat pads on one side. After the injection, animals were sutured, and analgesics injected. Animals were kept in a clean cage with drinking water supplemented with 1 µM 17-β-estradiol. Tumor xenografts were measured with callipers every 3 days, and tumor volume was determined using the formula: (length × width^2^) × (pi/6). At the end of the experiment, animals were anesthetized with a 1.5% isoflorane–air mixture and were killed by cervical dislocation.

Treatments were administered intraperitoneally twice weekly. One week after cell injection, mice were randomized and treated with TSA (0.25 mg/kg), doxorubicin (2 mg/kg mouse weight), or doxorubicin plus TSA. The control group was injected with sterile PBS.

Mice were maintained and treated in accordance with institutional guidelines of Vall d'Hebron University Hospital Care and Use Committee.

## Supplementary information

Supplementary information

Supplementary Figure 1

Supplementary Figure 2

Supplementary Figure 3

Supplementary Table 1

Supplementary Table 2

## Data Availability

GSE96064.
