# Supplementary material for: LOXL2-mediated H3K4 oxidation reduces chromatin accessibility in triple-negative breast cancer cells
Source: Oncogene. 2019 Aug 28;39(1):79–121. doi: 10.1038/s41388-019-0969-1 (PMC6937214; doi:10.1038/s41388-019-0969-1)
Supplement: Supplementary file 1 — Supplementary information [file 41388_2019_969_MOESM1_ESM.docx]

LOXL2-mediated H3K4 oxidation reduces chromatin accessibility in triple-negative breast cancer cells

J.P. Cebrià-Costa^1^, L. Pascual-Reguant^1^, A. Gonzalez-Perez^2^, G. Serra-Bardenys^1^, J. Querol^1^, M. Cosín^1^, G. Verde^3^, R.A. Cigliano^4^, W. Sanseverino^4^, S. Segura-Bayona^2^, A. Iturbide^5^, D. Andreu^6^, P. Nuciforo^1^, C. Bernado-Morales^1,7^, V. Rodilla^1^, J. Arribas^1,7,8,9^, J. Yelamos^10^, A. Garcia de Herreros^6,10^, T.H. Stracker,^2^ S. Peiró^1*^

^1^Vall d’Hebron Institute of Oncology (VHIO), 08035 Barcelona, Spain. ^2^Institute for Research in Biomedicine (IRB Barcelona), Barcelona Institute of Science and Technology, 08028 Barcelona, Spain.  ^3^Faculty of Medicine and Health Sciences, Universitat Internacional de Catalunya, Barcelona, Spain. ^4^Sequentia Biotech SL, Comte d'Urgell, 240, Barcelona, Spain. ^5^Institute of Epigenetics and Stem Cells, Helmoholtz Zentrum München, D-81377 München, Germany. ^6^Departament de Ciències Experimentals i de la Salut, Universitat Pompeu Fabra, Barcelona, Spain. ^7^Centro de Investigación Biomédica en Red en Oncología (CIBERONC), 08035 Barcelona, Spain. ^8^Institució Catalana de Recerca I Estudis Avançats (ICREA), Barcelona, Spain. ^9^Departament de Bioquímica y Biología Molecular, Universitat Autónoma de Barcelona, Bellaterra, Spain. ^10^Programa de Recerca en Càncer, Institut Hospital del Mar d’Investigacions Mèdiques (IMIM), Barcelona, Spain.

*Corresponding author. Email: speiro@vhio.net; Vall d’Hebron Institute of Oncology, c/ Natzaret 115-117, 08035 Barcelona, Spain; phone: 34-932 543 450 (8683)

**Figure legends**

**Supplementary Figure 1**. Western blot with the indicated antibodies in 293 cells infected with GFP (MOCKGFP+), LOXL2-IRES-GFP (LOXL2GFP+), or LOXL2mut-IRES-GFP (LOXL2mutGFP+).

**Supplementary Figure 2**. Percentage of the indicated chromosome alterations per total number of cells in Control and LOXL2 KD conditions in two independent experiments, with a representative image of each condition. **P*< 0.05.

**Supplementary Figure 3.** (**A**) Caspase-3 activation analyzed by Western blot with the indicated antibodies. Tubulin was used as a loading control. Intervening lanes were removed as indicated. (**B**) Heat map and gene ontology (GO) analysis of differentially expressed genes by RNA-seq in control and *LOXL2* knockdown samples.

**Supplementary Table 1.** List of the antibodies and working dilutions used in the manuscript.

**Supplementary Table 2.** List of the primers used in the manuscript.
