# Supplementary figures and images for: LOXL2-mediated H3K4 oxidation reduces chromatin accessibility in triple-negative breast cancer cells
Source: Oncogene. 2019 Aug 28;39(1):79–121. doi: 10.1038/s41388-019-0969-1 (PMC6937214; doi:10.1038/s41388-019-0969-1)

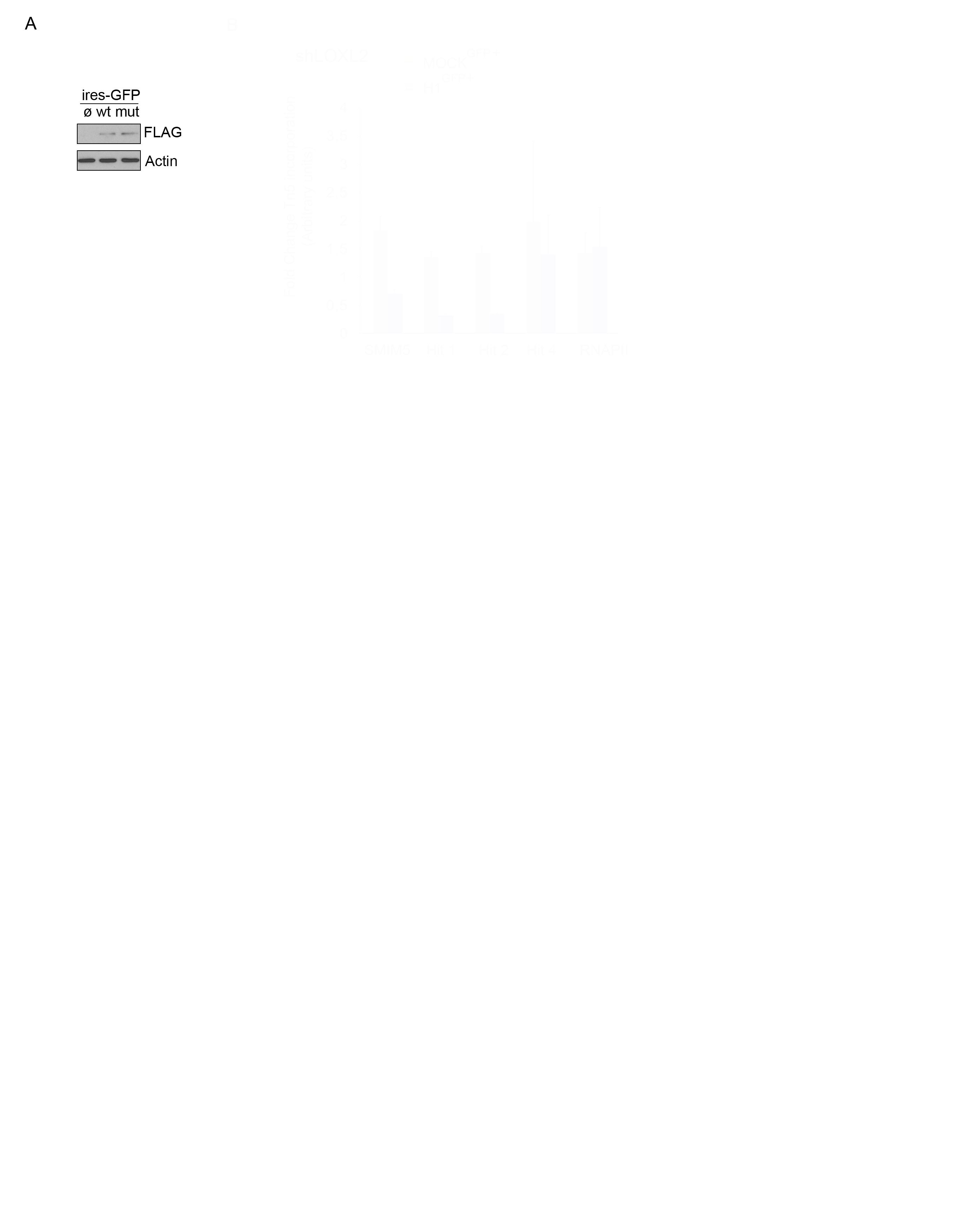

Supplement: Supplementary file 2 — Supplementary Figure 1 [file 41388_2019_969_MOESM2_ESM.jpg]

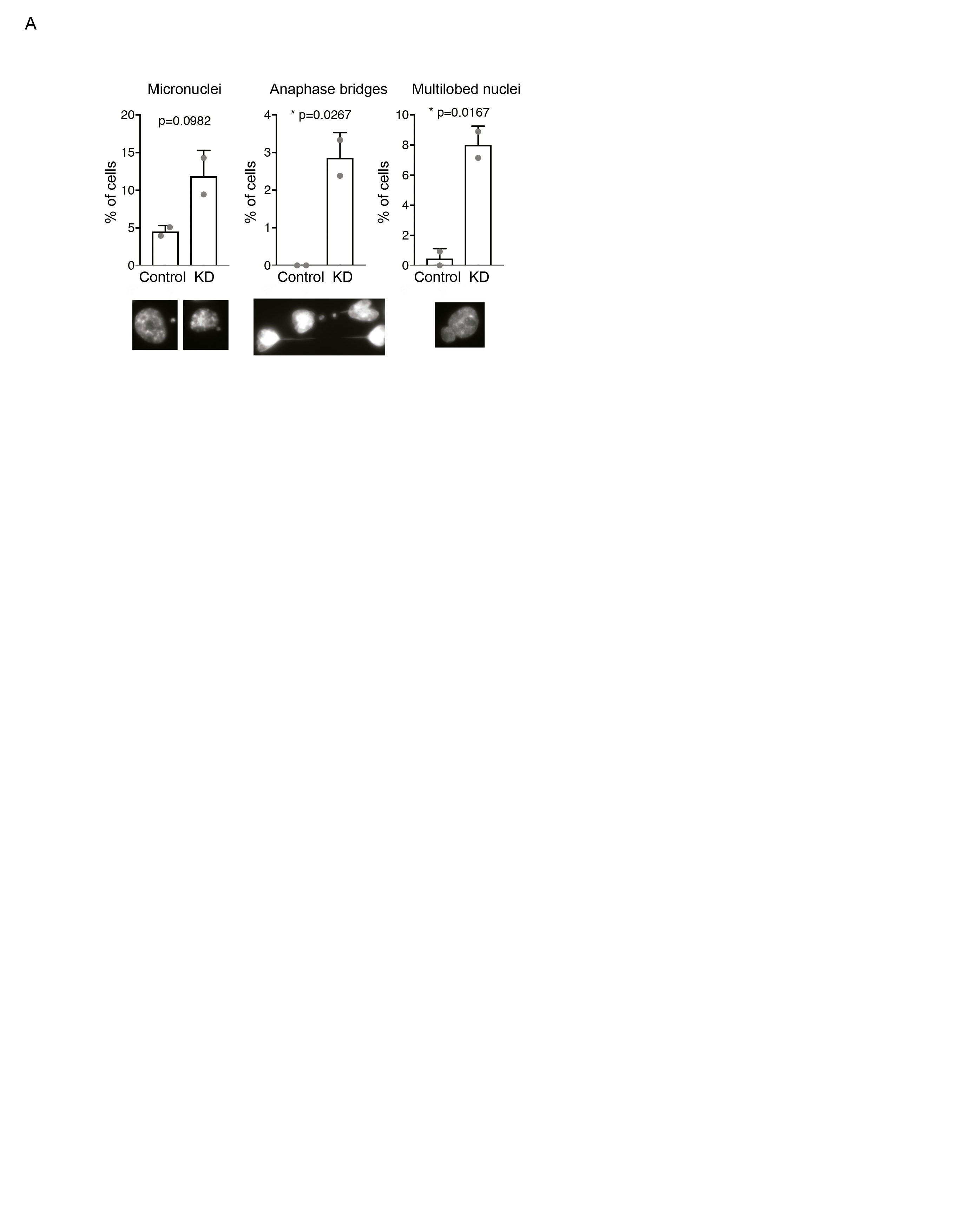

Supplement: Supplementary file 3 — Supplementary Figure 2 [file 41388_2019_969_MOESM3_ESM.jpg]

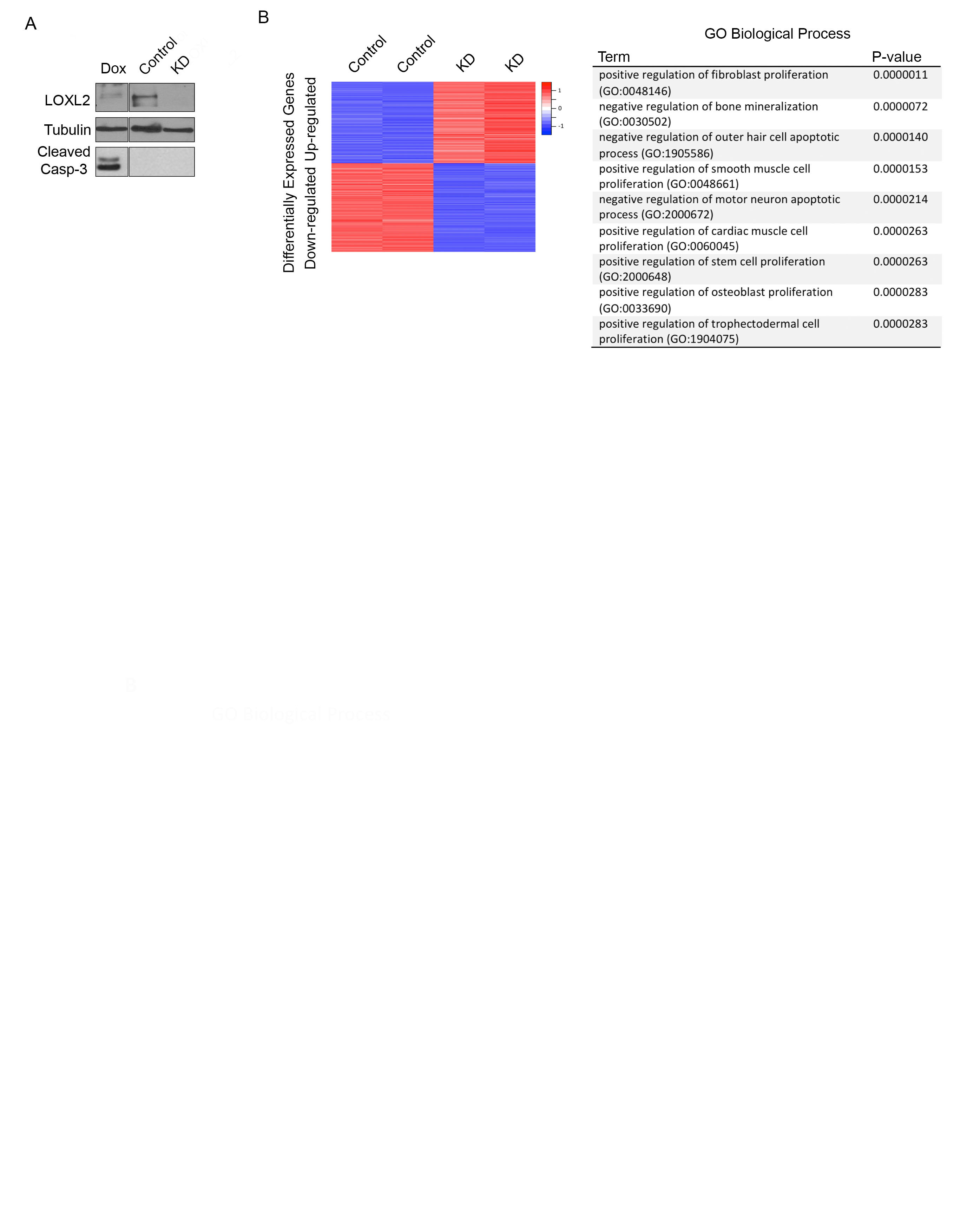

Supplement: Supplementary file 4 — Supplementary Figure 3 [file 41388_2019_969_MOESM4_ESM.jpg]
