# Supplementary material for: LOXL2-mediated H3K4 oxidation reduces chromatin accessibility in triple-negative breast cancer cells
Source: Oncogene. 2019 Aug 28;39(1):79–121. doi: 10.1038/s41388-019-0969-1 (PMC6937214; doi:10.1038/s41388-019-0969-1)
Supplement: Supplementary file 5 — Supplementary Table 1 [file 41388_2019_969_MOESM5_ESM.docx]

| **Protein** | **Species** | **Provider** | **Reference** | **Dilution** |
| --- | --- | --- | --- | --- |
| **Flag** | rabbit | Sigma | F7425 | WB 1:5000 |
| **LOXL2** | rabbit | Novus | NP1-32954 | WB 1:1000 |
| **H3K4me3** | rabbit | Millipore | 07-473 | WB 1:2000 |
| **H3K9me3** | rabbit | Millipore | 07-442 | WB 1:2000 |
| **Phospho-histone H2AX (S139)** | mouse | Millipore | 05-636 | IF 1:200 |
| **GFP** | rabbit | Abcam | ab6556 | IF 1:2000 |
| **H3** | rabbit | Abcam | Ab1791 | WB 1:10000 / ChIP 5 mg |
| **53BP1** | rabbit | Novus | NB100-904 | IF 1:1000 |
| **CHK2 clone 7** | mouse | Millipore | 05-649 | WB 1:1000 |
| **Phospho-CHK1 (S317)** | rabbit | Bethyl Labs | A300-163A | WB 1:1000 |
| **KAP1** | rabbit | Abcam | ab10484 | WB 1:1000 |
| **Phospho-KAP1 (S824)** | rabbit | Bethyl Labs | A300-767A | WB 1:1000 |
| **cleaved caspase 3 (Asp175)** | rabbit | Cell Signaling Tech. | 9661 | WB 1:1000 |
| **pH3 (S10)** | rabbit | Millipore | 06-570 | WB 1:1000 |
| **Tubulin** | mouse | Sigma | T9026 | WB 1:50000 |
| **H3K4ox** | rabbit | generated in-house |  | WB 1:10000 / ChIP 5 mg |
