# Supplementary material for: LOXL2-mediated H3K4 oxidation reduces chromatin accessibility in triple-negative breast cancer cells
Source: Oncogene. 2019 Aug 28;39(1):79–121. doi: 10.1038/s41388-019-0969-1 (PMC6937214; doi:10.1038/s41388-019-0969-1)
Supplement: Supplementary file 6 — Supplementary Table 2 [file 41388_2019_969_MOESM6_ESM.docx]

| **Primer** | **Direction** |  | **Sequence (5´–3´)** |
| --- | --- | --- | --- |
| **CDH1** | Forward |  | AACCCTCAGCCAATCAGCGG |
| **CDH1** | Reverse |  | GTTCCGACGCCACTGAGAGG |
| **SMIM 5** | Forward |  | CAAGGGAAATTGTCCAGACTTC |
| **SMIM 5** | Reverse |  | AGTAGCTGGGACTACAGGCG |
| **GR1** | Forward |  | ATAAGCTTTTTGATGTGCTGCTG |
| **GR1** | Reverse |  | GGAGCTGCTAGCATTCCTTCTAA |
| **GR2** | Forward |  | TAACTCATTTATGAGGCCAACGTC |
| **GR2** | Reverse |  | CTTGTGCATATTGAACCAGCCT |
| **GR3** | Forward |  | GCATTTGGGTTGGAGTCTATCA |
| **GR3** | Reverse |  | ACCATTGTAGAAGATAGTGTGGCG |
| **GR4** | Forward |  | AGCTCTGTAAGAACTAAGATTGGGCT |
| **GR4** | Reverse |  | TTCTATCTTCACGGTTCTCCAAGA |
| **HPRT** | Forward |  | ATTCACGCGATGACTGGA |
| **HPRT** | Reverse |  | AGGCTCACTAGGTAGCCGTG |
| **POLR2A** | Forward |  | CTGAGTCCGGATGAACTGGT |
| **POLR2A** | Reverse |  | ACCCATAAGCAGCGAGAAAG |
